# Targeted DNA demethylation of the *Arabidopsis* genome using the human TET1 catalytic domain

**DOI:** 10.1073/pnas.1716945115

**Published:** 2018-02-14

**Authors:** Javier Gallego-Bartolomé, Jason Gardiner, Wanlu Liu, Ashot Papikian, Basudev Ghoshal, Hsuan Yu Kuo, Jenny Miao-Chi Zhao, David J. Segal, Steven E. Jacobsen

**Affiliations:** ^a^Department of Molecular, Cell, and Developmental Biology, University of California, Los Angeles, CA 90095;; ^b^Molecular Biology Institute, University of California, Los Angeles, CA 90095;; ^c^Department of Human Genetics, David Geffen School of Medicine, University of California, Los Angeles, CA 90095;; ^d^Genome Center, Medical Investigation of Neurodevelopmental Disorders Institute, and Department of Biochemistry and Molecular Medicine, University of California, Davis, CA 95616;; ^e^Howard Hughes Medical Institute, University of California, Los Angeles, CA 90095

**Keywords:** *Arabidopsis*, targeted demethylation, TET1, CRISPR/dCas9 SunTag, artificial zinc finger

## Abstract

DNA methylation is an important epigenetic modification involved in gene regulation and transposable element silencing. Changes in DNA methylation can be heritable and, thus, can lead to the formation of stable epialleles. A well-characterized example of a stable epiallele in plants is *fwa*, which consists of the loss of DNA cytosine methylation (5mC) in the promoter of the *FLOWERING WAGENINGEN* (*FWA*) gene, causing up-regulation of *FWA* and a heritable late-flowering phenotype. Here we demonstrate that a fusion between the catalytic domain of the human demethylase TEN-ELEVEN TRANSLOCATION1 (TET1cd) and an artificial zinc finger (ZF) designed to target the *FWA* promoter can cause highly efficient targeted demethylation, *FWA* up-regulation, and a heritable late-flowering phenotype. Additional ZF–TET1cd fusions designed to target methylated regions of the *CACTA1* transposon also caused targeted demethylation and changes in expression. Finally, we have developed a CRISPR/dCas9-based targeted demethylation system using the TET1cd and a modified SunTag system. Similar to the ZF–TET1cd fusions, the SunTag–TET1cd system is able to target demethylation and activate gene expression when directed to the *FWA* or *CACTA1* loci. Our study provides tools for targeted removal of 5mC at specific loci in the genome with high specificity and minimal off-target effects. These tools provide the opportunity to develop new epialleles for traits of interest, and to reactivate expression of previously silenced genes, transgenes, or transposons.

## Significance

DNA methylation is an epigenetic modification involved in gene silencing. Studies of this modification usually rely on the use of mutants or chemicals that affect methylation maintenance. Those approaches cause global changes in methylation and make difficult the study of the impact of methylation on gene expression or chromatin at specific loci. In this study, we develop tools to target DNA demethylation in plants. We report efficient on-target demethylation and minimal effects on global methylation patterns, and show that in one case, targeted demethylation is heritable. These tools can be used to approach basic questions about DNA methylation biology, as well as to develop new biotechnology strategies to modify gene expression and create new plant trait epialleles.

DNA methylation is involved in silencing genes and transposable elements (TE). In contrast to many organisms where methylation is largely erased and reestablished in each generation (1), changes in DNA methylation patterns in plants can be transmitted through sexual generations to establish stable epigenetic alleles (2–6). For example, complete loss of 5mC in the promoter of the *FLOWERING WAGENINGEN* (*FWA*) gene causes stable *fwa* epialleles that have been found in flowering-time mutant screens (7) and in strong DNA methylation mutants (2, 4, 8). This loss of 5mC at the *FWA* promoter activates *FWA* expression that is responsible for the late-flowering phenotype observed in *fwa* epialleles (4). DNA methylation in plants occurs in different cytosine contexts -CG, CHG, and CHH- (where H is A, T, or C) and is controlled by different DNA methyltransferases (DNMTs). METHYLTRANSFERASE 1 (MET1), a homolog of DNA methyltransferase 1 (DNMT1), is responsible for the maintenance of symmetric methylation in the CG context (9). CHROMOMETHYLASE 3 (CMT3) and CHROMOMETHYLASE 2 (CMT2) are responsible for the maintenance of CHG and CHH methylation, respectively, at pericentromeric regions and long TEs (10–13). Finally, DOMAINS REARRANGED METHYLTRANSFERASE 2 (DRM2), a homolog of DNA methyltransferase 3 (DNMT3), is involved in the maintenance of CHH at borders of long TEs in pericentromeric heterochromatin as well as small TEs in euchromatin (10, 13–16), and represents the last step of the de novo methylation pathway in plants, called RNA-directed DNA methylation (RdDM) (17). Plants also have an active DNA demethylation system driven by REPRESSOR OF SILENCING 1 (ROS1) and three other related glycosylase/lyase enzymes (18–20). These enzymes recognize DNA methylcytosines and initiate DNA demethylation through a base excision-repair process (21).

Thus far, studies aiming to understand the effect of DNA methylation on gene expression have relied on the use of mutants defective in genes involved in the DNA methylation machinery, or chemicals to inhibit methylation maintenance, such as 5-azacytidine or zebularine (8, 22–24). Both approaches, genetic and chemical, have the disadvantage of affecting DNA methylation at a genome-wide scale, making it difficult to study the impact of DNA methylation on gene expression and chromatin at specific loci. Therefore, it is important to create tools in plants that allow the manipulation of DNA methylation in a more locus-specific manner.

A previous study in *Arabidopsis* has shown that a fusion of the RdDM component SU(VAR)3-9 HOMOLOG 9 (SUVH9) to an artificial zinc finger designed to target the *FWA* promoter (ZF108) is able to target methylation to the *FWA* promoter, silencing *FWA* expression and rescuing the late-flowering phenotype of the *fwa-4* epiallele (25). Unfortunately, no equivalent tool has been developed in plants for targeted DNA demethylation.

In animals, controlled removal of 5mC by TEN-ELEVEN TRANSLOCATION1 (TET1) has been achieved through targeting the human TET1 catalytic domain (TET1cd) to specific regions of the genome by fusing it to DNA binding domains such as ZFs, TAL effectors, or CRISPR/dCas9 (26–34).

TET1 causes demethylation of DNA through oxidation of 5mC to 5-hydroxymethylcytosine (5hmC), 5-formylcytosine (5fC), and 5-carboxylcytosine (5caC) (35). This is followed by either the passive removal of methylation through the failure of DNA methylation maintenance after DNA replication or the active removal of DNA methylation by glycosylase-mediated base excision repair (36). While plants do not contain TET enzymes, a previous study has shown that overexpression of the human TET3 catalytic domain in *Arabidopsis* can cause changes in DNA methylation levels at rDNA loci (37). However, both hypermethylation and hypomethylation were observed in this study, making the results difficult to interpret, and only effects at rDNA loci were examined. This finding suggests that while TET enzymes may potentially be used in plants to manipulate DNA methylation, improved strategies are needed to use TET enzymes to manipulate 5mC in a locus-specific manner.

In this work, we describe the development of different tools to target locus-specific DNA demethylation in *Arabidopsis*. We have fused human TET1cd to artificial ZFs designed to target two different loci in the *Arabidopsis* genome. We have also adapted the CRISPR/dCas9 SunTag system to target DNA demethylation in plants (26, 38). Using both targeting platforms—ZF or SunTag—we observe precise DNA demethylation and associated changes in gene expression over the targeted regions, with only small effects on genome-wide methylation or gene expression. The development of tools for targeted demethylation in plants creates exciting avenues for the study of locus-specific effects of DNA methylation on gene expression and the chromatin landscape. These tools should also allow for the generation of new epialleles, and the manipulation of TE expression levels to create insertional mutations and study genome evolution.

## Results and Discussion

### Expression of ZF108–TET1cd Causes Late Flowering and *FWA* Activation.

**Fig. 1.**
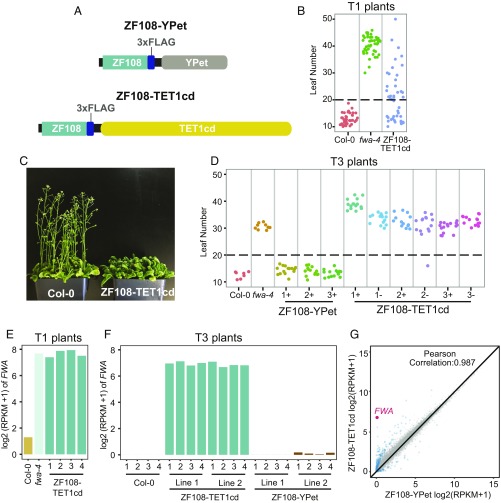
ZF108–TET1cd expression causes heritable late flowering and *FWA* up-regulation. (*A*) Schematic representation of the ZF108–YPet (*Upper*) and the ZF108–TET1cd fusions (*Lower*). (*B*) Flowering time of Col-0, *fwa-4*, and ZF108–TET1cd T1 plants. (*C*) Col-0 plants and a representative ZF108–TET1cd T3 line grown side by side to illustrate the differences in flowering time. (*D*) Flowering time of Col-0, *fwa-4*, three independent lines containing ZF108–YPet and three independent lines of ZF108–TET1cd. For each independent ZF108–TET1cd line, two different T3 populations were scored, one containing the ZF108–TET1cd transgene (+) and one that had the transgene segregated away in the T2 generation (−). For *B* and *D*, individual plants are depicted as colored dots. Leaf number corresponds to the total number of rosette and caulinar leaves after flowering. All plants above the dashed line are considered late flowering. (*E*) Bar graph showing *FWA* expression of one plant of Col-0, *fwa-4*, and four representative late-flowering T1 plants expressing ZF108–TET1cd. (*F*) Bar graph showing *FWA* expression of four biological replicates of Col-0 plants and two representative T3 lines expressing ZF108–TET1cd and ZF108–YPet. (*G*) Scatterplot comparing gene expression of ZF108–TET1cd lines and ZF108–YPet lines. Values were calculated using four biological replicates of two independent lines for ZF108–TET1cd and ZF108–YPet. Gray dots indicate nondifferentially expressed genes. Blue dots indicate differentially expressed genes. A fourfold change and false-discovery rate less than 0.05 was used as a cutoff. *FWA* expression is highlighted in red.

In animals, targeted removal of 5mC has been achieved by using the human TET1cd (26–34). To test if TET1cd can be used in plants for targeted demethylation, we fused human TET1cd to ZF108 and expressed the fusion under the control of the constitutive *UBIQUITIN 10* (*UBQ10*) promoter from *Arabidopsis* (Fig. 1*A*). ZF108 was previously shown to target DNA methylation to the promoter of the *FWA* gene when fused to the RdDM component SUVH9 (25). The *FWA* promoter is normally methylated in wild-type Col-0 plants, causing silencing of *FWA*. Demethylation of the promoter in *met1* mutants or *fwa-4* epialleles is heritable over generations, triggers the ectopic expression of *FWA*, and causes a late-flowering phenotype (4). Therefore, this methylation-dependent visual phenotype can be exploited as a readout for successful targeted demethylation. We screened T1 plants expressing ZF108–TET1cd in the Col-0 background and found 25 of 57 that displayed a late-flowering phenotype, suggesting *FWA* activation (Fig. 1*B*).

We then studied the stability of the late-flowering phenotype over generations by analyzing the flowering time of T3 lines that either retained the ZF108–TET1cd transgene (T3^+^) or had the transgene segregated away in the T2 generation (T3^−^). Both T3^+^ and T3^−^ lines retained a late-flowering phenotype, consistent with a loss of methylation at the *FWA* promoter (Fig. 1*C*). Importantly, control plants expressing a fusion of ZF108 to the fluorescent protein YPet (ZF108–YPet) (39) did not show any effect on flowering time, suggesting that the late-flowering phenotype observed is not simply a consequence of ZF108 binding to the *FWA* promoter (Fig. 1*D*).

To test if the late-flowering phenotype observed was due to *FWA* up-regulation, we performed RNA-seq of Col-0, *fwa-4*, and four representative late-flowering T1 plants expressing ZF108–TET1cd (Fig. 1*E*), as well as four biological replicates of Col-0, and two representative T3 lines expressing ZF108–TET1cd or ZF108–YPet (Fig. 1*F*). *FWA* expression was dramatically increased in ZF108–TET1cd compared with Col-0 and ZF108–YPet and had a similar expression level as *fwa-4*, indicating that the late-flowering phenotype observed was due to *FWA* overexpression (Fig. 1 *E* and *F*). A genome-wide gene-expression analysis showed very few additional changes and revealed *FWA* as the most up-regulated gene in the ZF108–TET1cd lines compared with ZF108–YPet control lines (Fig. 1*G*). These results suggest successful removal of methylation at the *FWA* promoter and, importantly, very few off-target effects due to ZF108–TET1cd expression.

### Targeted Demethylation at the *FWA* Promoter Is Specific and Heritable.

**Fig. 2.**
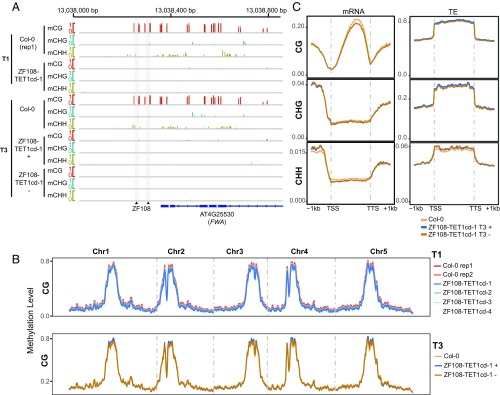
Targeted demethylation at the *FWA* promoter is specific and heritable. (*A*) Screenshot of CG, CHG, and CHH methylation levels over the *FWA* promoter in Col-0 and a representative ZF108–TET1cd T1 line (*Upper*). Screenshot of CG, CHG, and CHH methylation levels over the *FWA* promoter in Col-0 and a representative ZF108–TET1cd T3 line for which WGBS was done in a plant containing the ZF108–TET1cd construct [ZF108-TET1cd-1 (+)] and in a plant that had segregated away the transgene already in the T2 generation [ZF108–TET1cd-1 (−)] (*Lower*). Gray vertical lines indicate the ZF108 binding sites in the *FWA* promoter. 5′ proximal representation of the *FWA* transcribed region is depicted in blue with filled squares indicating the UTRs and diamond lines indicating introns. (*B*) Genome-wide distribution of CG methylation in two Col-0 plants and four representative T1 ZF108–TET1cd plants (*Upper*) as well as one Col-0 plant and one T3 plant containing the ZF108–TET1cd-1 (+) and a T3 plant that had segregated away the transgene already in the T2 generation [ZF108-TET1cd-1 (−)] (*Lower*). (*C*) Metaplot of CG, CHG, and CHH methylation levels over protein coding genes and TEs in Col-0, ZF108–TET1cd-1 (+) and ZF108–TET1cd-1 (−) T3 plants. Methylation level is depicted on the *y* axis of all graphs.

We then analyzed methylation levels at the *FWA* promoter by McrBC digestion in different ZF108–TET1cd late-flowering T1 plants. All lines showed a large reduction in DNA methylation, similar to that observed in *fwa-4* plants (Fig. S1). To confirm these results, we performed whole-genome bisulfite sequencing (WGBS) of Col-0, four representative T1 ZF108–TET1cd plants, as well as two representative T3 ZF108–TET1cd lines, including one T3^+^ and one T3^−^. We observed complete demethylation over the *FWA* promoter in all four representative T1 lines, resembling the methylation pattern seen in *fwa-4* (Fig. 2*A* and Fig. S2*A*). Additionally, both T3^+^ and T3^−^ lines showed complete demethylation of the *FWA* promoter (Fig. 2*A* and Fig. S2*A*), indicating that the targeted DNA demethylation is heritable, even in the absence of the transgene. Interestingly, loss of methylation spanned the entire methylated region of the *FWA* promoter—∼500 base pairs (bp)—including cytosines a few hundred base pairs away from the ZF108 binding sites. To assess the specificity of TET1cd-mediated demethylation, we looked at methylation levels in a larger region flanking the *FWA* gene (Fig. S2*B*), as well as analyzed genome-wide methylation levels (Fig. 2 *B* and *C* and Fig. S3). We found that genome-wide CG, CHG, and CHH methylation levels were very similar to the wild-type Col-0 control, indicating that targeted demethylation using ZF108–TET1cd was very specific. These results are consistent with the RNA-seq results presented in Fig. 1*G* that showed very few changes in genome-wide expression patterns in plants expressing ZF108–TET1cd compared with controls.

### Targeted Demethylation at the *CACTA1* Promoter Using ZFCACTA1–TET1cd Fusions.

**Fig. 3.**
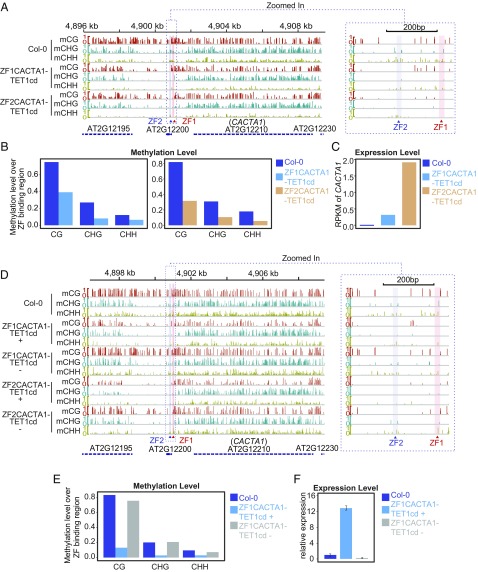
Targeted demethylation of *CACTA1* using ZF–TET1cd fusions. (*A*) Screenshot showing CG, CHG, and CHH methylation over the *CACTA1* region in Col-0, and one T1 plant each for ZF1CACTA1–TET1cd and ZF2CACTA1–TET1cd. (*B*) Bar graphs depicting the methylation levels in the region comprising 200 bp upstream and downstream of the ZF1CACTA1 or ZF2CACTA1 binding sites for Col-0 and one representative T1 plant of ZF1CACTA1–TET1cd and ZF2CACTA1–TET1cd. (*C*) Bar graph showing *CACTA1* expression in Col-0 and one representative T1 plant of ZF1CACTA1–TET1cd and ZF2CACTA1–TET1cd. RPKM values are indicated. (*D*) Screenshot showing CG, CHG, and CHH methylation over the *CACTA1* region in Col-0, and one T2 plant containing the transgene (+) and one that had segregated it away (−) for ZF1CACTA1–TET1cd and ZF2CACTA1–TET1cd lines. In *A* and *D*, a red vertical line indicates the ZF1 binding site and a purple vertical line indicates the ZF2 binding site in the promoter region of *CACTA1*. A zoom in of the targeted region is shown (*Right*). Methylation level is shown for WGBS. (*E*) Bar graph depicting the methylation levels in the region comprising 200 bp upstream and downstream of the ZF1CACTA1 binding site for Col-0 and one T2 plant containing the ZF1CACTA1–TET1cd transgene (+) or one that had segregated it away (−). (*F*) Bar graph showing relative expression by qRT-PCR of *CACTA1* over *IPP2* in Col-0, one T2 plant containing the ZF1CACTA1–TET1cd transgene (+) or one that had it segregated away (−). Mean values ± SD (*n* = 2, technical replicates).

To test the ability of the ZF–TET1cd fusions to target demethylation at a heterochromatic locus, we fused TET1cd to two ZFs (ZF1CACTA1 and ZF2CACTA1) designed to target the promoter region of *CACTA1*, a TE that resides in an area of the genome with a very high level of DNA methylation and H3K9me2 (40, 41). Five and nine independent T1 plants containing ZF1CACTA1–TET1cd and ZF2CACTA1–TET1cd, respectively, were screened for demethylation at the *CACTA1* promoter by McrBC. The ZF1CACTA1–TET1cd and ZF2CACTA1–TET1cd T1 lines showing the greatest demethylation were selected for further analysis by WGBS (Fig. 3 *A* and *B*). Compared with Col-0, ZF1CACTA1–TET1cd and ZF2CACTA1–TET1cd plants showed a loss of methylation in all three sequence contexts that extended up to 2 kb upstream of the ZF binding sites (Fig. 3 *A* and *B*). To assess the specificity of ZF1CACTA1–TET1cd and ZF2CACTA1–TET1cd targeted demethylation, we analyzed genome-wide methylation levels (Fig. S4*A*) and methylation over all protein-coding genes or TEs (Fig. S4*B*). We found that methylation across the entire genome was slightly reduced compared with the Col-0 control in both the ZF1CACTA1–TET1cd and ZF2CACTA1–TET1cd lines, indicating a partial nonspecific global demethylation.

Next, we performed RNA-seq to test if targeted demethylation had an impact on *CACTA1* expression. In both lines tested, a significant increase in *CACTA1* transcript levels was observed (Fig. 3*C*), indicating that targeted demethylation at this region is sufficient to reactivate *CACTA1* expression.

To test heritability of targeted demethylation in these lines, we performed WGBS on T2 plants containing the transgene (+) or T2 plants that had segregated it away (−). Plants that had lost the ZFCACTA1–TET1cd transgenes showed reestablishment of methylation to levels similar to Col-0 control (Fig. 3 *D* and *E*). This is in contrast to *FWA*, where methylation loss was stable in the absence of the transgene, and is likely a consequence of the incomplete removal of DNA methylation at the *CACTA1* region that is then able to attract the methylation machinery through self-reinforcing mechanisms (25). To study if this recovery of methylation in the absence of the transgene translates to the resilencing of *CACTA1*, we analyzed the expression of *CACTA1* in ZF1CACTA1–TET1cd (+) and (−) plants. Consistent with the methylation levels observed, *CACTA1* expression was detected in the presence of the transgene, while its expression was silenced to wild-type levels in the absence of ZF1CACTA1–TET1cd (Fig. 3*F*).

Interestingly, T2 plants containing the transgenes showed an increase in global demethylation compared with T1 plants (Fig. S4 *A* and *C*), indicating that the continuous presence of the transgene over generations can increase genome-wide effects. Moreover, consistent with the recovery of methylation in the absence of the transgene observed within the *CACTA1* region, global methylation returned to wild-type levels when the transgene was lost (Fig. S4 *C* and *D*).

### Targeted Demethylation at the *FWA* Promoter Using SunTag–TET1cd.

**Fig. 4.**
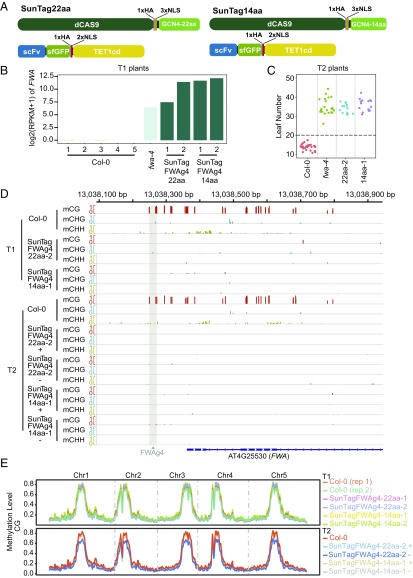
Targeted demethylation at the *FWA* promoter using SunTag-TET1cd. (*A*) Schematic representation of the SunTag-22aa (*Left*) and SunTag-14aa (*Right*) systems. (*B*) Bar graph showing *FWA* expression in five Col-0, one *fwa-4*, and two T1 plants each for SunTagFWAg4-22aa and SunTagFWAg4-14aa. (*C*) Flowering time of Col-0, *fwa-4*, and one representative SunTagFWAg4-22aa and SunTagFWAg4-14aa T2 line. (*D*) Screenshot of CG, CHG, and CHH methylation levels over the *FWA* promoter in Col-0, one representative SunTagFWAg4-22aa and SunTagFWAg4-14aa T1 line, and one representative T2 plant of the same lines containing the transgene (+) or that had segregated it away (−). A gray vertical line indicates the FWAgRNA-4 binding site (FWAg4) in the *FWA* promoter. 5′ proximal representation of the *FWA* transcribed region is depicted in blue with filled squares indicating the UTRs and diamond lines indicating introns. (*E*) Genome-wide CG methylation levels in two Col-0 plants, two T1 lines each for SunTagFWAg4-22aa and SunTagFWAg4-14aa (*Upper*), as well as one Col-0, one T2 plant containing the transgene (+) or one that had segregated it away (−) for representative SunTagFWAg4-22aa and SunTagFWAg4-14aa lines (*Lower*). Methylation level is depicted on the *y* axis.

While ZFs can efficiently target demethylation to specific loci in the genome, the design of new ZFs can be laborious and expensive. We therefore developed a plant-optimized CRISPR/dCas9-based SunTag–TET1cd system similar to one previously used to target demethylation in animals and shown to be more effective than direct fusions of TET1cd to dCas9 (26). In this system, dCas9 is fused to a C-terminal tail containing a variable number of tandem copies of peptide epitopes (GCN4). In a separate module, a single-chain variable fragment (scFv) antibody that recognizes the peptide epitopes is fused to a superfolder-GFP (sfGFP) followed by an effector protein (38) (Fig. 4*A*). We adapted the SunTag–TET1cd system for use in *Arabidopsis* by expressing both the dCas9 and the scFv modules under the control of the constitutive *UBQ10* promoter. We created two versions of the epitope tail fused to dCas9, one containing a 22-aa linker separating each epitope similar to the one used in Morita et al. (26), and one containing a 14-aa linker separating each epitope (Fig. 4*A*). To preserve the components used in previous successful SunTag constructs, we cloned TET1cd downstream of the scFv-sfGFP module, and added two SV40-type nuclear localization signals (NLSs) to allow plant nuclear localization. We utilized a single gRNA (FWAg4) driven by the U6 promoter designed to target the ZF108 binding sequence in the *FWA* promoter. Two of nine Col-0 transgenic plants containing SunTag–FWAg4–22aa-TET1cd (SunTagFWAg4-22aa) and two of three Col-0 transgenic plants containing SunTag–FWAg4–14aa-TET1cd (SunTagFWAg4-14aa) showed a late-flowering phenotype. Consistent with this phenotype, RNA-seq on two SunTagFWAg4-22aa and SunTagFWAg4-14aa T1 late-flowering plants showed dramatic *FWA* overexpression similar to that of *fwa-4* (Fig. 4*B*). In addition, quantification of the flowering time of a representative T2 line expressing SunTagFWAg4-22aa and one expressing SunTagFWAg4-14aa confirmed a late-flowering phenotype similar to *fwa-4* plants (Fig. 4*C*).

We then performed WGBS on two SunTagFWAg4-22aa and SunTagFWAg4-14aa T1 lines and T2 progeny that had the transgene (+) or had segregated it away (−) (Fig. 4*D* and Fig. S5). In all cases, we observed efficient demethylation at the *FWA* promoter that was stable in the absence of the transgenes, suggesting that both SunTagFWAg4-22aa and SunTagFWAg4-14aa are able to target heritable demethylation at the *FWA* promoter. To study potential off-target effects, we examined methylation levels in a wider region surrounding *FWA* (Fig. S5*B*), and also analyzed genome-wide methylation (Fig. 4*E* and Fig. S6). Methylation levels over regions flanking *FWA* did not show significant changes compared with Col-0 (Fig. S5*B*). Similarly, genome-wide DNA methylation levels were similar between the SunTagFWAg4-22aa and SunTagFWAg4-14aa plants and Col-0 control (Fig. 4*E* and Fig. S6).

### Targeted Demethylation at the *CACTA1* Promoter Using SunTag–TET1cd.

**Fig. 5.**
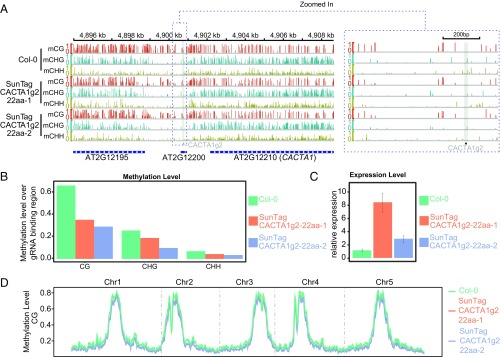
Targeted demethylation of *CACTA1* using SunTag–TET1cd. (*A*) Screenshot of CG, CHG, and CHH methylation levels over the *CACTA1* region in Col-0 and two representative SunTagCACTA1g2-22aa T1 lines. A gray vertical line indicates the gRNA binding site in the promoter region of *CACTA1*. A zoom in of the targeted region is shown (*Right*). (*B*) Bar graphs depicting the methylation levels in the region comprising 200 bp upstream and downstream of the gRNA binding sites for Col-0 and two representative SunTagCACTA1g2-22aa T1 plants. (*C*) Bar graph showing relative expression by qRT-PCR of *CACTA1* over *IPP2* in Col-0 and two representative SunTagCACTA1g2-22aa T1 plants. Mean values ± SD (*n* = 2, technical replicates). (*D*) Genome-wide CG methylation levels in Col-0 and two representative SunTagCACTA1g2-22aa T1 plants. Methylation level is depicted on the *y* axis.

To test the ability of the SunTag22aa–TET1cd fusion to target demethylation at a heterochromatic locus, we utilized a single gRNA (CACTA1g2) driven by the U6 promoter designed to target the same region that we targeted with the ZFCACTA1–TET1cd fusions (Fig. 3). Six T1 plants containing SunTagCACTA1g2-22aa-TET1cd (SunTagCACTA1g2-22aa) were screened for demethylation at the *CACTA1* promoter by McrBC. The two plants showing the greatest demethylation were selected for further analysis by WGBS (Fig. 5*A*). Consistent with the results obtained with ZFCACTA1, SunTagCACTA1g2-22aa plants showed a loss of methylation in all three sequence contexts that extended up to 2 kb upstream of the gRNA binding site (Fig. 5 *A* and *B*), causing the up-regulation of *CACTA1* expression (Fig. 5*C*). Moreover, genome-wide methylation analysis indicated no observable differences between wild-type Col-0 and the SunTagCACTA1g2-22aa lines (Fig. 5*D* and Fig. S7). Overall, these results confirm that the SunTag approach is effective for targeting demethylation in plants without a major effect on global methylation levels. We also tested the impact of expressing our SunTag–TET1cd systems in wild-type Col-0 plants in the absence of a gRNA that directs the construct to a specific location. Flowering time of T1 plants expressing these constructs was unaffected (Fig. S8*A*). Also, methylation levels at the *FWA* promoter or *CACTA1* region were similar to Col-0 (Fig. S8 *B* and *C*), and global methylation levels did not show any significant differences compared with a Col-0 control (Fig. S8*D*).

## Conclusion

In this work, we present two independent methods for targeting DNA demethylation in *Arabidopsis*. We first fused the human TET1cd to an artificial ZF protein, ZF108, designed to target the *FWA* promoter. Col-0 plants expressing this construct showed highly specific demethylation and reactivation of *FWA* with virtually no genome-wide effects on DNA methylation or gene expression. In *Arabidopsis* plants grown under long-day conditions (16-h light/8-h dark), flowering is established around 10–12 days after germination (42). The fact that T1 plants expressing ZF108–TET1cd showed a late-flowering phenotype indicates that demethylation of the *FWA* promoter occurred during the early stages of development of the T1 plants. Surprisingly, the targeted demethylation at *FWA* comprised a large region, almost 500 bp surrounding the ZF108 binding site. This could be due to direct access of ZF108–TET1cd to these cytosines. Another possibility is that loss of methylation in the distal regions from the ZF108 binding site is a secondary effect of *FWA* reactivation or 3D chromatin conformation that would place distal regions in proximity to the targeted region where ZF108–TET1cd is bound.

We also generated new ZFs to target the promoter of the *CACTA1* TE whose expression is also controlled by DNA methylation (40). Importantly, this locus is located in pericentromeric heterochromatin, which is associated with long stretches of chromatin that are highly enriched in DNA methylation and H3K9 methylation (41), and may represent a more challenging environment for targeted approaches. Two different ZFs targeting TET1cd to the *CACTA1* promoter triggered loss of methylation up to 2 kb away from the ZF binding sites. These data, together with the data obtained for *FWA,* indicate that ZF fusions to TET1cd can cause demethylation hundreds of base pairs away from the targeted sequence.

Contrary to the heritable loss of methylation in the *FWA* promoter, targeted demethylation at *CACTA1* disappeared when the ZFCACTA1 transgenes were segregated away, showing that unlike *FWA*, methylation was quickly reestablished. The most likely explanation for this is that, contrary to the complete demethylation of the entire *FWA* methylated region, the incomplete demethylation of *CACTA1* leaves enough residual methylation to attract the RdDM machinery, probably via recruitment of RNA polymerase V (Pol V) by the methyl DNA binding proteins SUVH2 and SUVH9 (25). In addition, the MET1 CG methyltransferase would likely perpetuate and potentially amplify any remaining methylated CG sites. In this scenario, heritable demethylation might be more efficiently achieved by targeting the TET1cd to multiple adjacent locations to achieve a more complete demethylation. Alternatively, *CACTA1* remethylation may occur because other methylated regions in the genome with sequences homologous to *CACTA1* may be able to efficiently target remethylation *in trans* via siRNAs. Additional targeting experiments will be needed to determine the frequency with which targeted demethylation can be heritable.

While targeted demethylation using ZF108–TET1cd was very specific and showed negligible changes in genome-wide methylation compared with Col-0, lines expressing ZF1CACTA1–TET1cd, or ZF2CACTA1–TET1cd showed a varying amount of genome-wide hypomethylation. This variability highlights the importance to be selective with different ZFs, protein fusions, expression levels, and insertion events when using TET1cd to avoid genome-wide effects.

We also created a plant-optimized version of the SunTag–TET1cd system and showed that it can be successfully implemented in plants for targeted DNA demethylation at the *FWA* and *CACTA1* loci. Similar to the results obtained using ZFs, we observed very high on-target demethylation and gene activation, with small effects on genome-wide methylation levels. Resembling the ZF–TET1cd fusions, the demethylation extended well beyond the targeted region reaching a region of ∼2 kb in the case of SunTagCACTA1g2 lines. Morita et al. (26) reported that SunTag–TET1cd could also demethylate more than 200 bp in mammalian cells. In this case, it is reasonable to think that the TET1cd may be able to directly access long stretches of DNA considering the extension of the long epitope tail and the simultaneous recruitment of many molecules of TET1cd.

In summary, our results show highly efficient targeted demethylation in plants by using artificial ZFs or SunTag fused to TET1cd with limited off-target effects. As a result of their efficiency and specificity, they provide an ideal way to study the role of DNA methylation at specific loci and circumvent the need to use DNA methylation mutants or chemicals that reduce methylation. Moreover, these tools may allow for the creation of new stable epialleles with traits of interest by activating genes normally silenced by DNA methylation. Other potential uses are for the reactivation of specific classes of transposons or the reactivation of previously silenced transgenes.

## Methods

### Plant Material and Growth Conditions.

All of the plants used in this study were in the Columbia-0 ecotype (Col-0) and were grown under long-day conditions. The *fwa-4* epiallele was selected from a *met1* segregating population (25). Transgenic plants were obtained by agrobacterium-mediated floral dipping (43). Plants were selected on 1/2 MS medium + Glufosinate 50 μg/mL (Goldbio), 1/2 MS medium + Hygromycin B 25 μg/mL (Invitrogen), or sprayed with Glufosinate (1:2,000 Finale in water). Flowering time was scored by counting the total number of rossette and caulinar leaves.

### ZF Design and Cloning.

#### Cloning of pUBQ10::ZF108_3xFlag_TET1cd.

For this purpose, a modified pMDC123 plasmid (44) was created, containing 1,990 bp of the promoter region of the *Arabidopsis UBQ10* gene upstream of a cassette containing the biotin ligase recognition peptide (BLRP) followed by the ZF108, previously described in Johnson et al. (25), and a 3xFlag tag. Both *UBQ10* promoter and BLRP_ZF108_3xFlag are upstream of the gateway cassette (Invitrogen) present in the original pMDC123 plasmid. The catalytic domain of the TET1 protein (TET1cd) was amplified from the plasmid pJFA334E9, a gift from Keith Joung, Harvard Medical School, Boston (Addgene plasmid #49237) (27), and cloned into the pENTR/D plasmid (Invitrogen) and then delivered into the modified pMDC123 by an LR reaction (Invitrogen), creating an in-frame fusion of the TET1cd cDNA with the upstream BLRP_ZF108_3xFlag cassette (Fig. 1*A*). Similarly, YPet was amplified from a YPet containing plasmid and cloned into the pENTR/D plasmid and then delivered to the modified pMDC123 by an LR reaction. Sequences of the modified pUBQ10::ZF108_3xFlag_TET1cd as well as pUBQ10::ZF108_3xFlag_YPet plasmids are provided in Dataset S1.

#### Cloning of pUBQ10::ZFCACTA1_3xFlag_TET1cd.

Two ZFs were designed to bind 18-bp sequences from the *CACTA1* promoter (42): ZF1CACTA1 (GTAGAGGGAAGTGAATAG) and ZF2CACTA1 (GTTGAGGAAAATGAGCTA). Amino acid sequences were obtained in silico using Codelt (zinc.genomecenter.ucdavis.edu:8080/Plone/codeit), selecting linker type “normal.” The resulting amino acid sequence was plant codon optimized and synthesized by IDT. A modified pMDC123 plasmid (44) was created, containing 1,990 bp of the promoter region of *Arabidopsis UBQ10* gene upstream of a cassette containing a unique HpaI restriction site, a 3xFlag tag, and the gateway cassette (Invitrogen) present in the original pMDC123 plasmid. The TET1cd was delivered from the pENTR/D_TET1cd plasmid described above into the modified pMDC123 by an LR reaction (Invitrogen), creating an in-frame fusion of the TET1cd cDNA with the upstream 3xFlag cassette. The different ZFCACTA1 were cloned in the unique HpaI restriction site in the modified pMDC123_3xFlag_TET1cd plasmid by In-Fusion (Takara). Sequences of the resulting plasmids: pUBQ10::ZF1CACTA1_3xFlag_TET1cd and pUBQ10::ZF2CACTA1_3xFlag_TET1cd are provided in Dataset S1. In an effort to make these reagents available for the academic community, the following plasmids are available through Addgene using the corresponding Addgene plasmid identification number: pUBQ10::ZF108_3xFlag_TET1CD (106432); pUBQ10::ZF1CACTA1_3xFlag_TET1cd (106433); pUBQ10::ZF2CACTA1_3xFlag_TET1cd (106434); pUBQ10::ZF108_3xFlag_YPet (106441).

### SunTag Design and Cloning.

Nucleic acid sequences of SunTag-22aa-TET1cd and SunTag-14aa-TET1cd were either PCR-amplified from Addgene plasmid #60903 and #60904, gifts from Ron Vale, University of California, San Francisco (38), or synthesized using GenScript services. The SunTag constructs were adapted from Tanenbaum et al. (38) to create a dCas9-based demethylation system in plants. dCas9+epitope tail (GCN4 × 10), scFv antibody, and the gRNA were cloned into a binary pMOA backbone vector (45) using In-Fusion (Takara). Expression of dCas9+epitope tail and scFv was controlled by the *UBQ10* promoter, and the gRNA was expressed using the U6 promoter. gRNA protospacer sequences are: FWAg4 (5′-acggaaagatgtatgggctt-3′) and CACTA1g2 (5′-gtcctcattgatagcagtag-3′).

The epitope tails fused to dCas9 consisted of 10 copies of the GCN4 peptide and either a 14-aa linker or a 22-aa linker separated each epitope. An extra SV40-type NLS was added to the dCas9+epitope sequence. Due to a lack of an effective NLS on the scFv–TET1cd fusion, two SV40-type NLSs were added for nuclear import of the antibody. These were preceded by 1xHA tag. Sequences of SunTagFWAg4-22aa-TET1cd, SunTagFWAg4-14aa-TET1cd, SunTagCACTA1g2-22aa-TET1cd, SunTagng22aa, and SunTagng14aa are provided in Dataset S1. In an effort to make these reagents available for the academic community, the following plasmids are available through Addgene using the corresponding Addgene plasmid identification number: SunTagFWAg4-22aa-TET1cd (106435); SunTagFWAg4-14aa-TET1cd (106436); SunTagCACTA1g2-22aa-TET1cd (106437); SunTagng22aa (106438); SunTagng14aa (106439).

### Quantitative Real-Time PCR.

RNA was extracted using Direct-zol RNA Miniprep kit (Zymo). For quantitative real-time PCR (qRT-PCR) involving ZF1CACTA1-TET1cd T2 plants, 600 ng of total RNA was used to prepare cDNA using the SuperScript III First-Strand Synthesis SuperMix (Invitrogen). For qRT-PCR involving SunTagCACTA1g2-22aa plants, 250 ng of total RNA was used to prepare cDNA using the SuperScript III First-Strand Synthesis SuperMix (Invitrogen). qRT-PCR of the *CACTA1* transcripts was done using the oligos (5′-agtgtttcaatcaaggcgtttc-3′) and (5′-cacccaatggaacaaagtgaac-3′). Values were normalized to the expression of the housekeeping gene *ISOPENTENYL PYROPHOSPHATE:DIMETHYLALLYL PYROPHOSPHATE ISOMERASE 2* (*IPP2*) using oligos (5′-gtatgagttgcttctccagcaaag-3′) and (5′-gaggatggctgcaacaagtgt-3′).

### McrBC–qRT-PCR.

CTAB-extracted DNA (1 µg) was digested using the McrBC restriction enzyme for 4 h at 37 °C. As a nondigested control, 1 µg of DNA was incubated in digestion buffer without McrBC enzyme for 4 h at 37 °C. qRT-PCR of the *FWA* promoter was done using the oligos (5′-*ttgggtttagtgtttacttg*-3′) and (5′-*gaatgttgaatgggataaggta*-3′). A control region methylated in Col-0 and unmethylated in *fwa-4* was amplified using the oligos (5′-*tgcaatttgtctgcttgctaatg*-3′) and (5′-*tcatttataatggacgatgcc*-3′). The ratio between the digested and nondigested samples was calculated.

### RNA-seq Analysis.

RNA was extracted using Direct-zol RNA Miniprep kit (Zymo). For RNAseq involving ZF108–TET1cd and ZF108–YPet plants, 75 ng of total RNA was used to prepare libraries using the Neoprep stranded mRNA-seq kit (Illumina). For RNA-seq involving ZF1CACTA1–TET1cd, ZF2CACTA1–TET1cd, SunTagFWAg4-14aa, and SunTagFWAg4-22aa plants, 1 μg of total RNA was used to prepare libraries using the TruSeq Stranded mRNA-seq kit (Illumina). Reads were first aligned to the TAIR10 gene annotation using Tophat (46) by allowing up to two mismatches and only keeping reads that mapped to one location. When reads did not map to the annotated genes, the reads were mapped to the TAIR10 genome. Number of reads mapping to genes were calculated by HTseq (47) with default parameters. Expression levels were determined by RPKM (reads per kilobase of exons per million aligned reads) using customized R scripts.

### WGBS Analysis.

DNA was extracted using a CTAB-based method and 100 ng were used to make libraries using the Nugen Ultralow Methyl-seq kit (Ovation). Raw sequencing reads were aligned to the TAIR10 genome using BSMAP (48) by allowing up to two mismatches and only retaining reads that mapped to one location. Methylation ratios are calculated by #C/(#C+#T) for all CG, CHG, and CHH sites. Reads with three consecutive methylated CHH sites were discarded since they are likely to be unconverted reads as described before (49).

### Metaplot of WGBS Data.

Metaplots of WGBS data were made using custom Perl and R scripts. Regions of interest were broken into 50 bins while flanking 1-kb regions were each broken into 25 bins. CG, CHG, and CHH methylation levels in each bin were then determined. Metaplots were then generated with R.

## Supplementary Material

Supplementary File

Supplementary File
