# Supplementary material for: Targeted DNA demethylation of the Arabidopsis genome using the human TET1 catalytic domain
Source: Proc Natl Acad Sci U S A. 2018 Feb 14;115(9):E2125–34. doi: 10.1073/pnas.1716945115 (PMC5834696; doi:10.1073/pnas.1716945115)
Supplement: Supplementary File [file pnas.201716945SI.pdf]

Supporting Information

Gallego-Bartolomé et al. 10.1073/pnas.1716945115

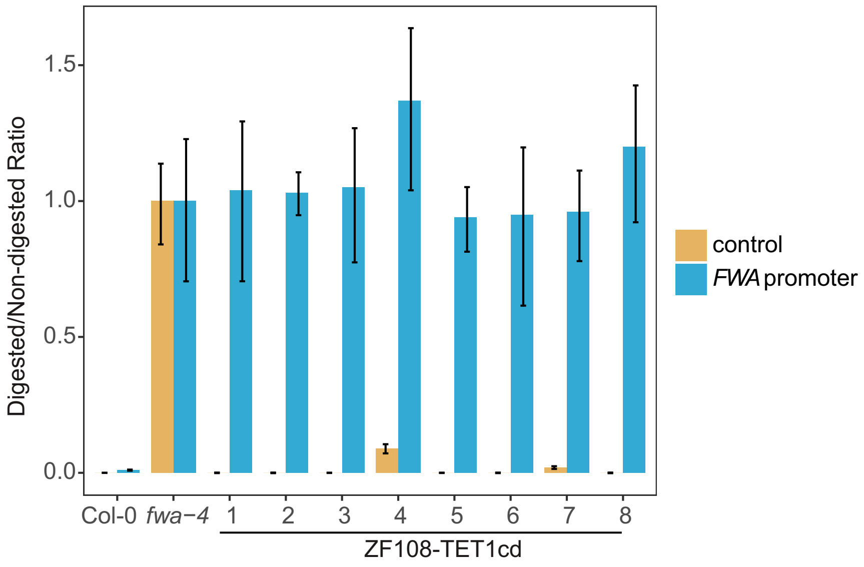

Fig. S1. McrBC–qRT-PCR indicates loss of methylation at *FWA* promoter in ZF108–TET1cd T1 plants. McrBC–qRT-PCR analysis of methylation in eight independent ZF108–TET1cd T1 plants, Col-0, and *fwa-4* controls. Mean values ± SD ($n = 3$, technical replicates). Oligos were designed to amplify the *FWA* promoter or a control region used to differentiate ZF108–TET1cd T1 plants in the Col-0 background from *fwa-4*.

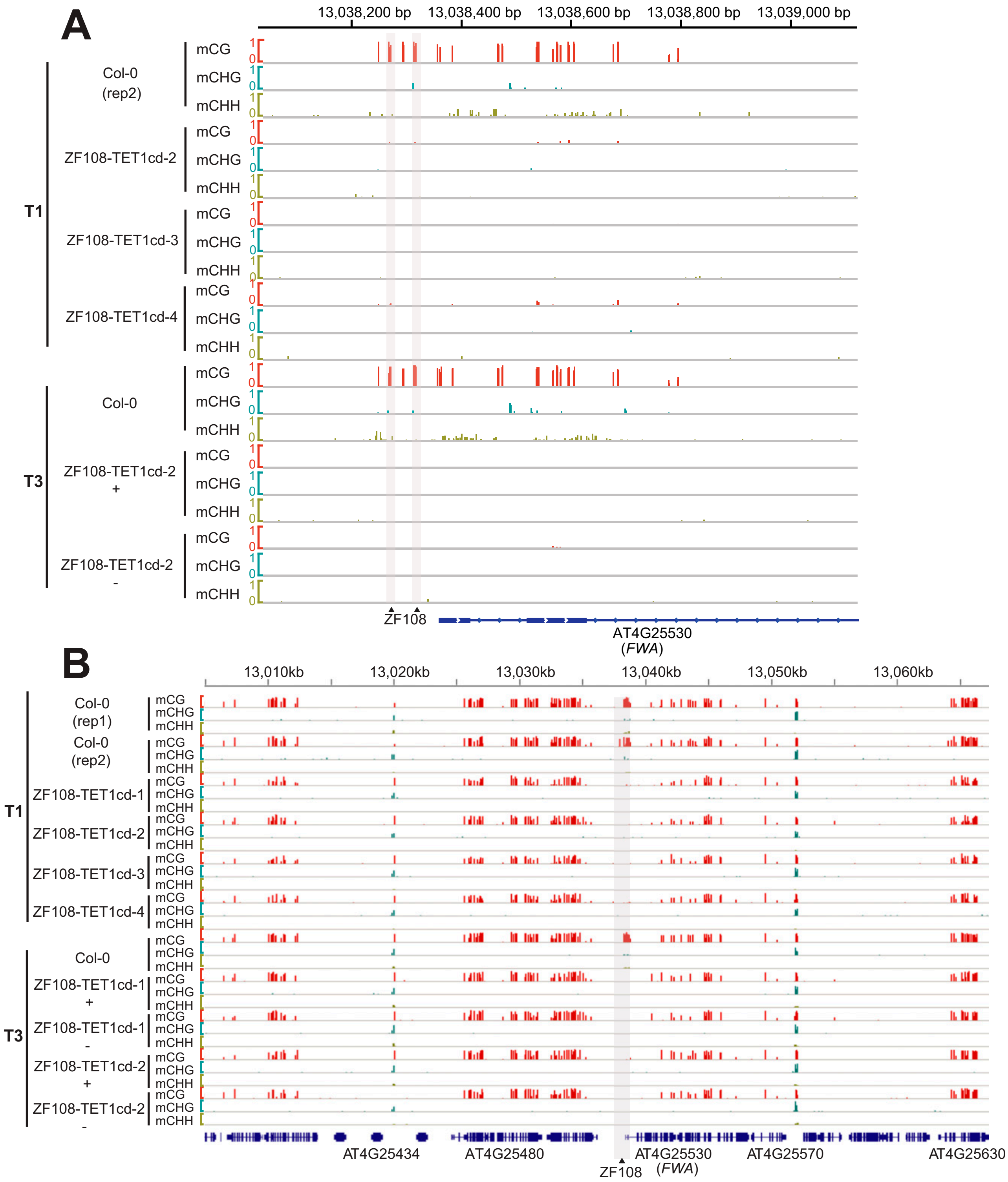

Fig. S2. ZF108–TET1cd specifically demethylates the *FWA* promoter. (*A*) Screenshot of CG, CHG, and CHH methylation levels over the *FWA* promoter in a second Col-0 control, three additional ZF108–TET1cd T1 plants and an additional ZF108–TET1cd-2 T3 line for which WGBS was done in a plant containing the ZF108-TET1cd construct [ZF108–TET1cd-2 (+)] and in a plant that had the transgene segregated away in the T2 generation [ZF108–TET1cd-2 (−)]. The gray vertical lines indicate the ZF108 binding sites in the *FWA* promoter. 5′ proximal representation of the *FWA* transcribed region is depicted in blue with filled squares indicating the UTRs and diamond lines indicating introns. (*B*) A zoomed out screenshot of CG, CHG, and CHH methylation levels over *FWA* and the surrounding regions in Col-0 controls, ZF108–TET1cd T1 plants and ZF108–TET1cd T3 plants [with (+) or without (−) the transgene]. For *A* and *B*, the gray vertical line indicates the ZF108 binding sites in the *FWA* promoter. Methylation level is depicted on the *y* axis. Red, blue and yellow brackets represent methylation ranges from 0 to 1, similar to *A*.

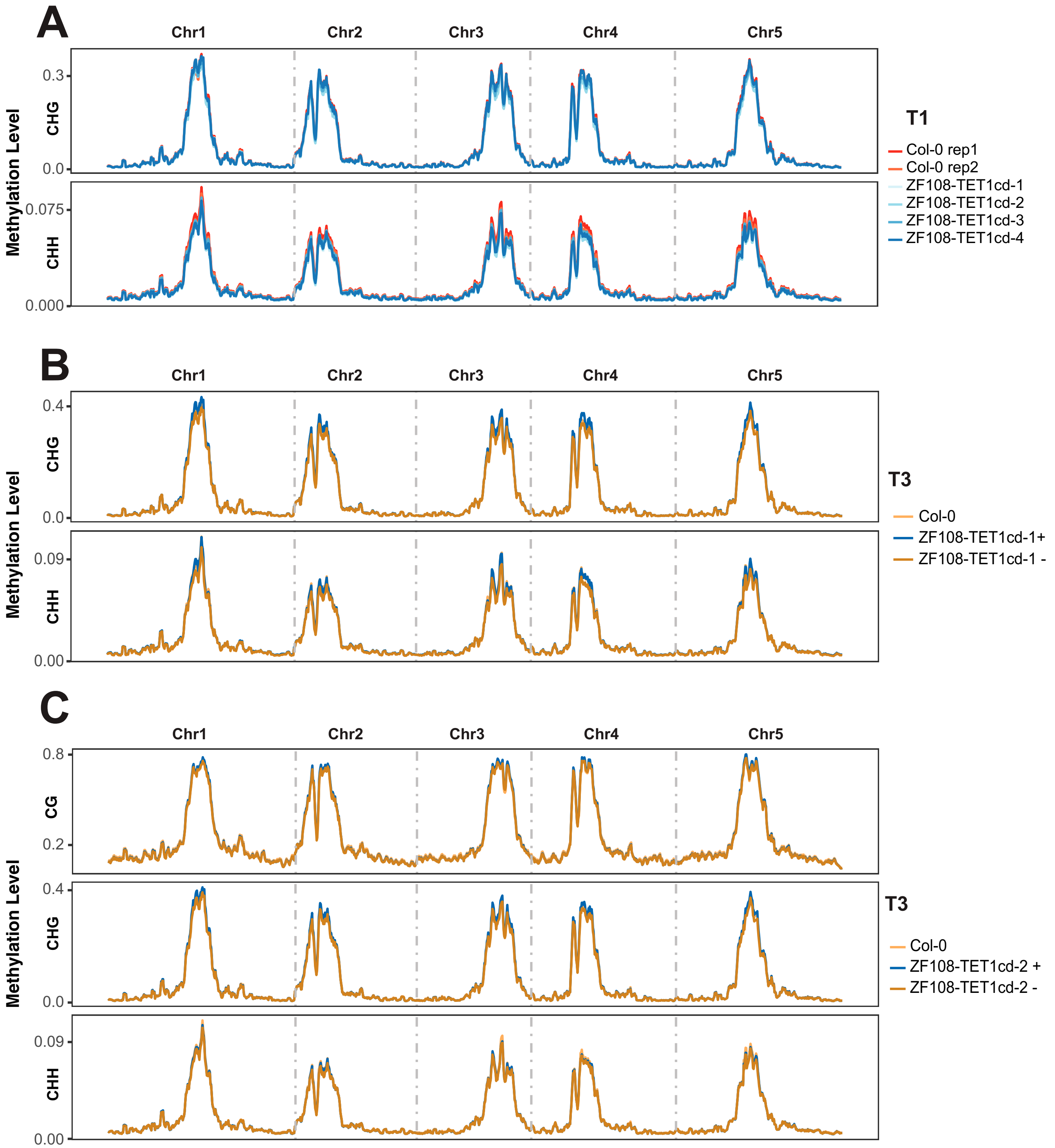

Fig. S3. ZF108–TET1cd specifically demethylates the *FWA* promoter. (*A*) Genome-wide distribution of CHG and CHH methylation in two Col-0 and four representative ZF108–TET1cd T1 plants. (*B*) Genome-wide distribution of CHG and CHH methylation in Col-0, and a representative ZF108–TET1cd-1 T3 line for which WGBS was done in a plant containing the ZF108–TET1cd transgene [ZF108–TET1cd-1 (+)] and in a plant that had the transgene segregated away in the T2 generation [ZF108–TET1cd-1 (–)]. (*C*) Genome-wide distribution of CG, CHG, and CHH methylation in Col-0, ZF108–TET1cd-2 (+), and ZF108–TET1cd-2 (–) T3 plants. Methylation level is depicted on the *y* axis of all graphs.

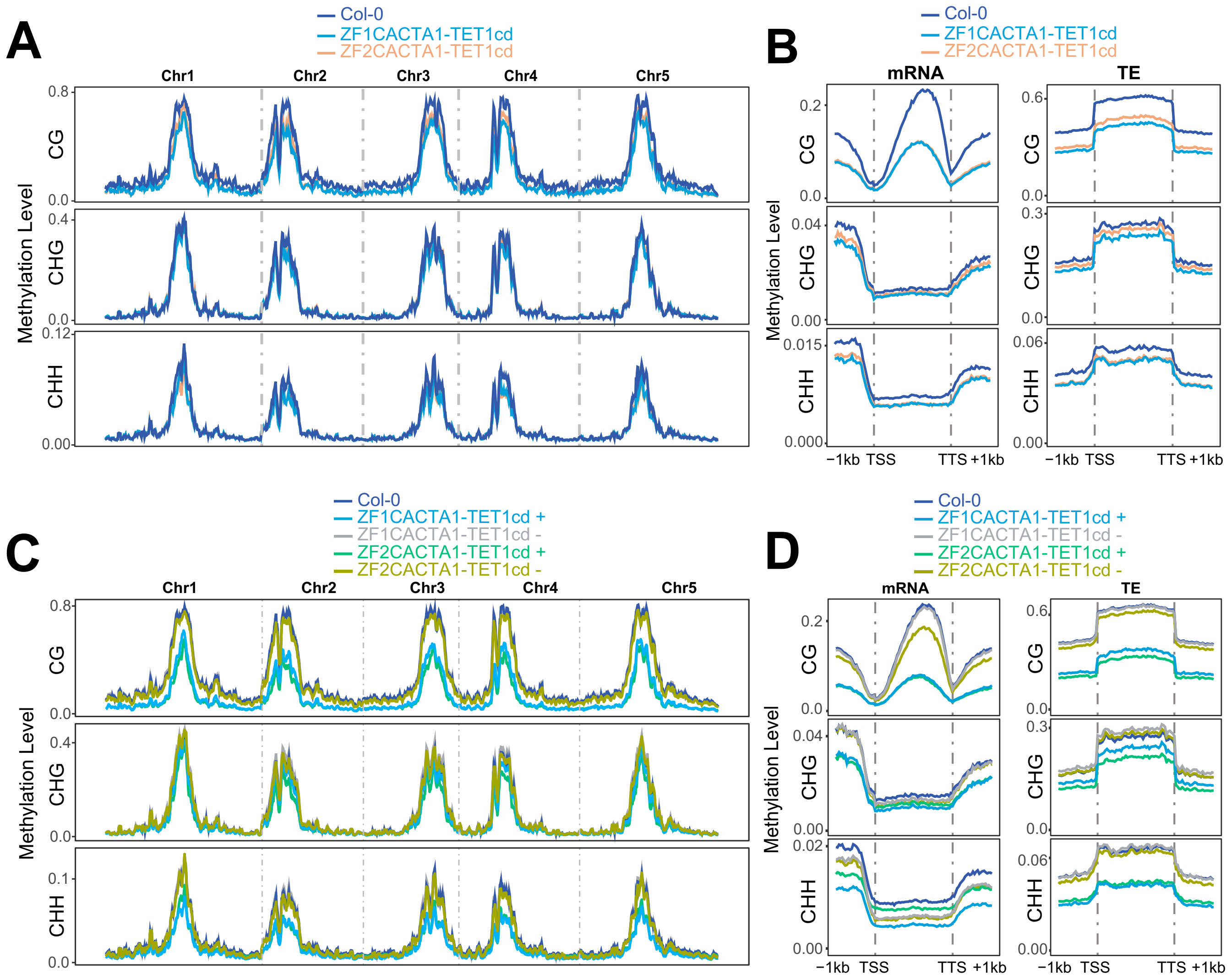

Fig. S4. ZF–TET1cd fusions targeting *CACTA1* show variable levels of nonspecific loss of methylation. (*A*) Genome-wide distribution of CG, CHG, and CHH methylation in Col-0 and one representative ZF1CACTA1–TET1cd and ZF2CACTA1–TET1cd T1 plant. (*B*) Metaplot showing CG, CHG, and CHH methylation levels over all protein coding genes and TEs in Col-0 and one representative ZF1CACTA1–TET1cd and ZF2CACTA1–TET1cd T1 plant. (*C*) Genome-wide distribution of CG, CHG, and CHH methylation in Col-0 and one T2 plant containing the transgene (+) and one that had segregated it away (–) for one representative ZF1CACTA1–TET1cd and ZF2CACTA1–TET1cd line. (*D*) Metaplot showing CG, CHG, and CHH methylation levels over all protein coding genes and TEs in Col-0 and one T2 plant containing the transgene (+) or one that had segregated it away (–) for one representative ZF1CACTA1–TET1cd and ZF2CACTA1–TET1cd line. Methylation level is depicted on the *y* axis of all graphs.

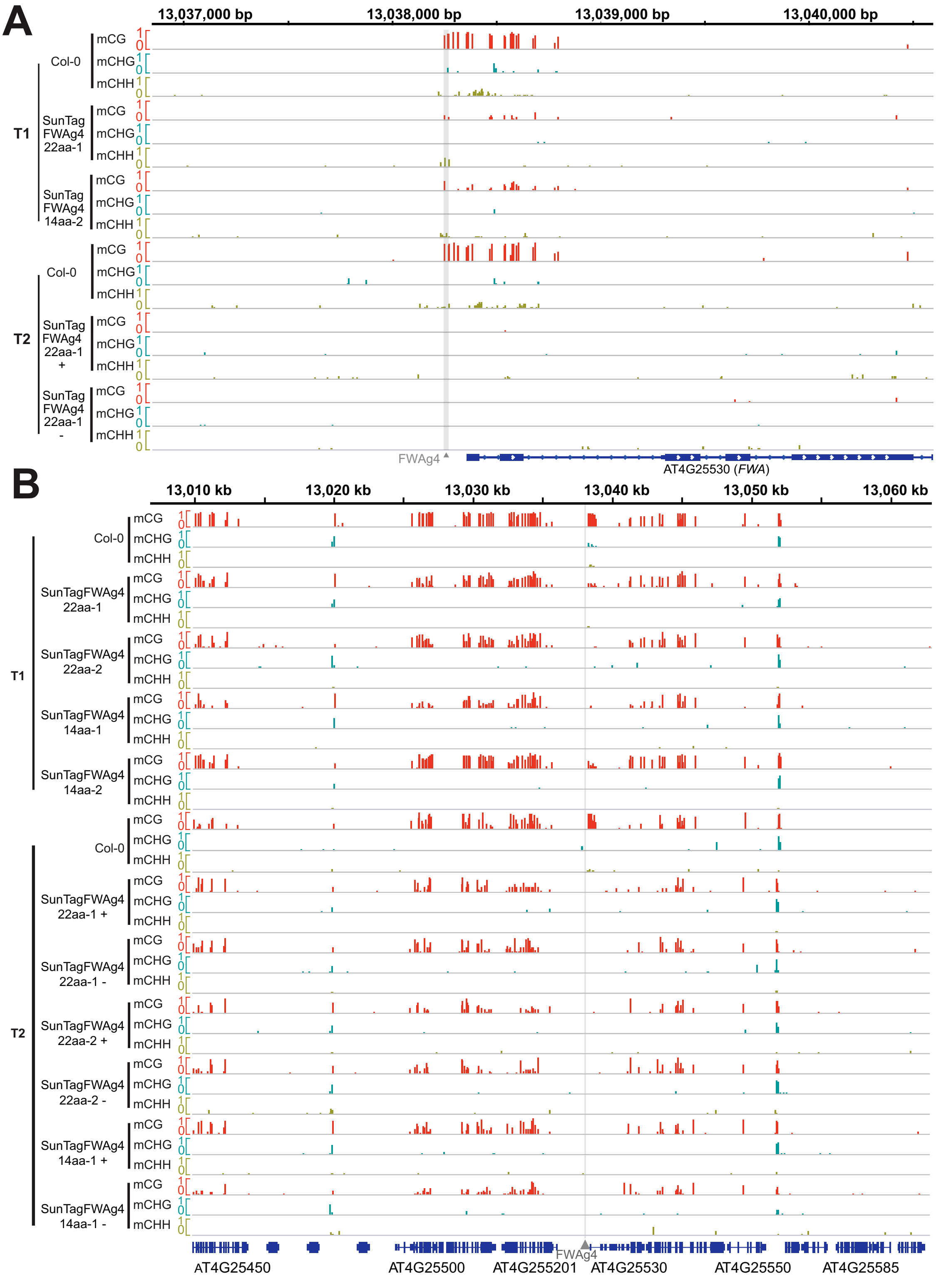

Fig. S5. SunTag-TET1cd specifically demethylates the *FWA* promoter. (*A*) Screenshot of CG, CHG, and CHH methylation levels over the *FWA* promoter in Col-0, a second representative SunTagFWAg4-22aa and SunTagFWAg4-14aa T1 line (results for the other representative line is shown in Fig. 4*D*), and one T2 plant containing the transgene (+) and one that had segregated it away (–) for a second representative SunTagFWAg4-22aa line (results for the other representative line is shown in Fig. 4*D*). (*B*) Zoomed out screenshot of CG, CHG, and CHH methylation levels over the *FWA* promoter and the surrounding regions in Col-0, two representative T1 plants each for SunTagFWAg4-22aa and SunTagFWAg4-14aa, as well as Col-0 and one T2 plant containing the transgene (+) or one that had segregated it away (–) for two representative SunTagFWAg4-22aa lines and one representative SunTagFWAg4-14aa line. For *A* and *B*, the gray vertical line indicates the FWAgRNA-4 binding site (FWAg4) in the *FWA* promoter.

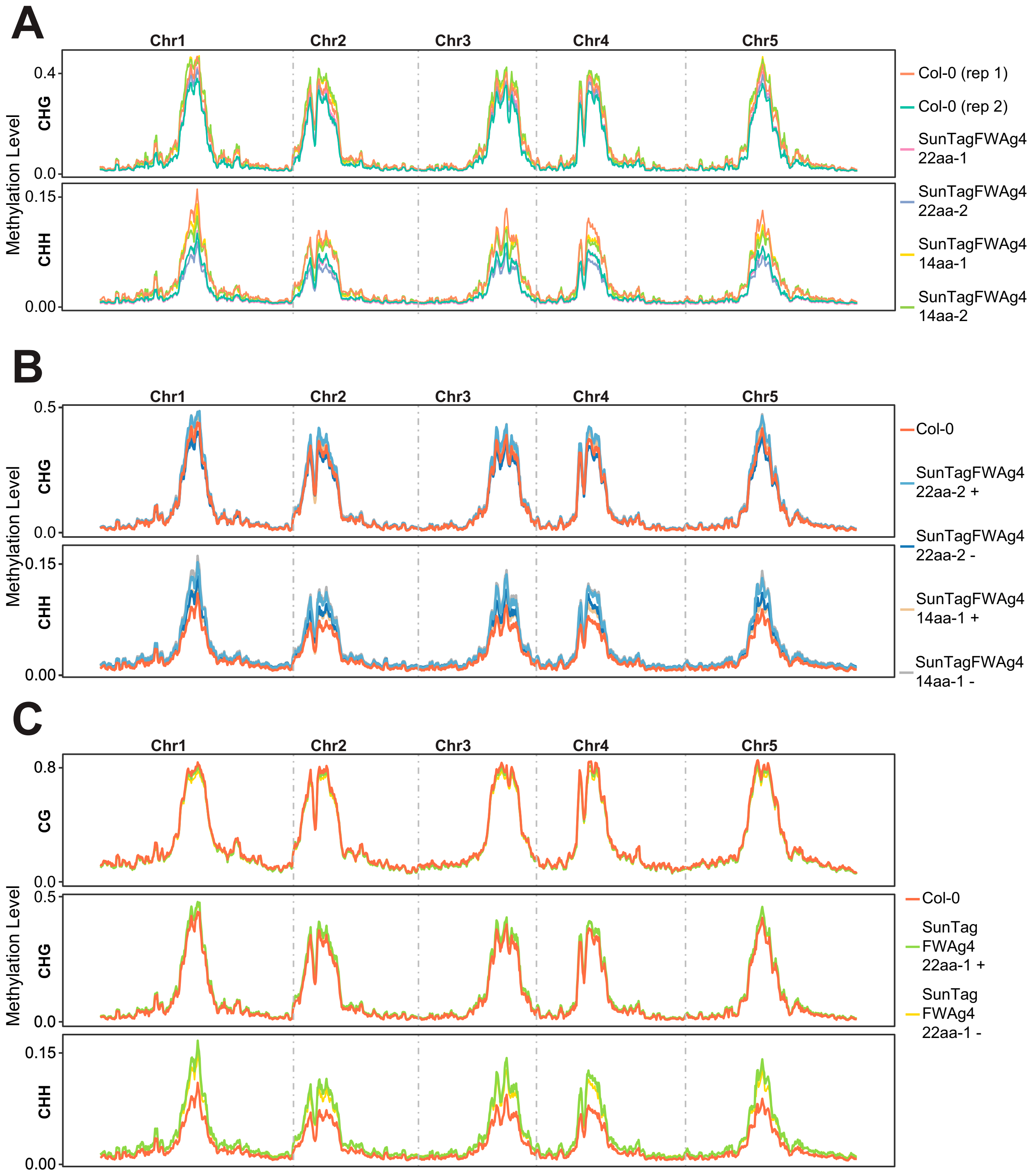

Fig. S6. *FWA*-targeted SunTag-TET1cd does not affect global DNA methylation levels. (*A*) Genome-wide distribution of CHG and CHH methylation in two independent Col-0, and two representative T1 lines each for SunTagFWAg4-22aa and SunTagFWAg4-14aa. (*B*) Genome-wide distribution of CHG and CHH methylation in Col-0, one T2 plant containing the transgene (+) and one that had segregated it away (–) for the SunTagFWAg4-22aa and SunTagFWAg4-14aa lines shown in Fig. 4*D*. (*C*) Genome-wide distribution of CG, CHG and CHH methylation in Col-0, one T2 plant containing the transgene (+) or one that had segregated it away (–) for a second representative line expressing SunTagFWAg4-22aa (SunTagFWAg4-22aa-1).

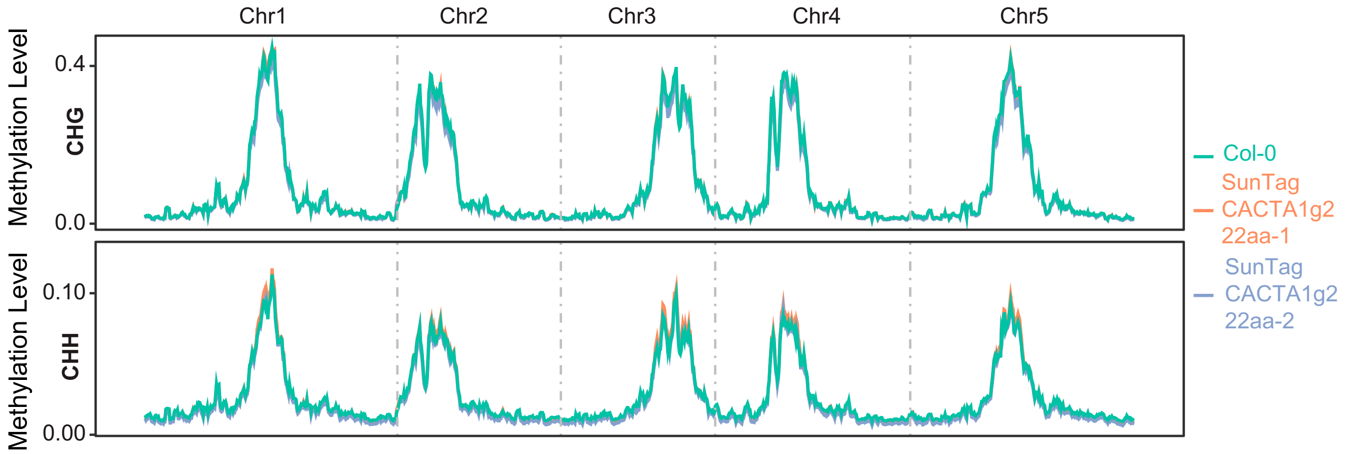

Fig. S7. *CACTA1*-targeted SunTag-TET1cd does not affect global DNA methylation levels. Genome-wide distribution of CHG and CHH methylation in Col-0 and two representative SunTagCACTA1g2-22aa T1 lines.

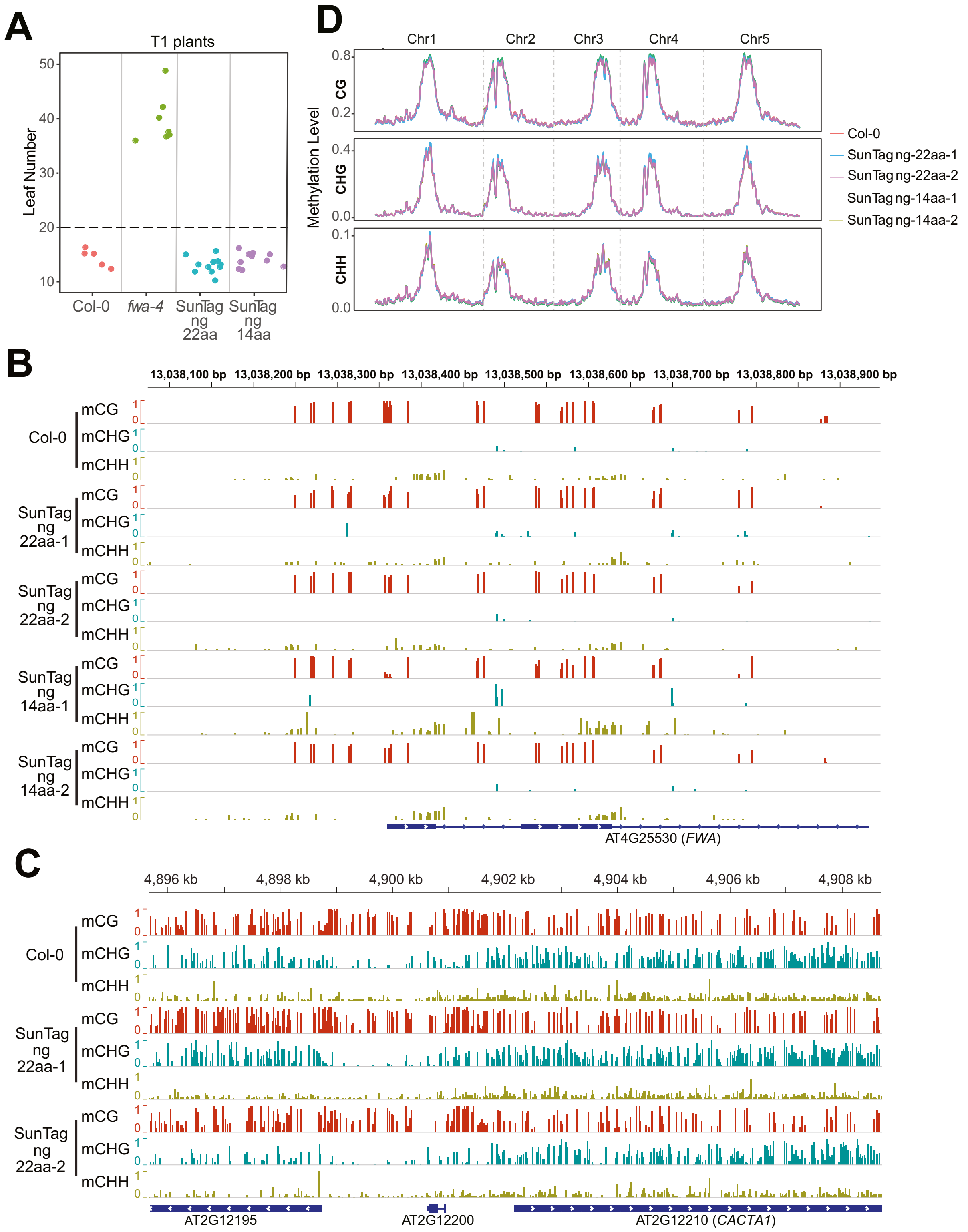

Fig. S8. SunTag-TET1cd with no gRNA (SunTagng) does not affect global DNA methylation levels. (*A*) Flowering time of Col-0, *fwa-4* controls, and SunTagng-22aa and SunTagng-14aa T1 plants. (*B*) Screenshot of CG, CHG, and CHH methylation levels over the *FWA* promoter in Col-0 and two representative T1 lines each for SunTagng-22aa and SunTagng-14aa. (*C*) Screenshot of CG, CHG, and CHH methylation levels over the *CACTA1* region in Col-0 and two representative T1 lines for SunTagng-22aa. (*D*) Genome-wide distribution of CG, CHG, and CHH methylation in Col-0 and two representative T1 lines each for SunTagng-22aa and SunTagng-14aa.

Dataset S1. Nucleotide sequences of the different ZF–TET1cd and SunTag–TET1cd fusions used in this work

Dataset S1
