# Supplementary material for: Targeted DNA demethylation of the Arabidopsis genome using the human TET1 catalytic domain
Source: Proc Natl Acad Sci U S A. 2018 Feb 14;115(9):E2125–34. doi: 10.1073/pnas.1716945115 (PMC5834696; doi:10.1073/pnas.1716945115)
Supplement: Supplementary File [file pnas.1716945115.sd01.pdf]

pUBQ10::ZF108_3xFlag_TET1cd

pUBQ10
BLRP
ZF108
3xFlag
TET1cd
OCS term

AGTCTAGCTCAACAGAGCTTTTAACCCAAATTGGTACAATAGAATACAACTTTAGAT
CATAATTCTCAAAAGAAAGAGATTCCTTAGCTATTCTATCTGCCACTCCATTTCCTTC
TCGGCTTGTATGCACAAGCATAAAATCCTCAAACTTGCTAAGTAGATACTTTATGTC
TTGGATAATTGGATTGAGACTTGACAAGCATAACTTTCATGTAACCAAAGACACAAG
TTGCTGAGAATCCACCTCAAAAATGATCTTCCTATAATTGAATCGGGATAATGACAG
CACAGCCCATCTAAGAGCCTCCACTTCTACTTCCAGCACGCTTCTTACTTTTACCAC
AGCTCTTGCACCTAACCATAACACCTTCCCTGTATGATCGCGAAGCACCCACCCTA
AGCCACATTTTAATCCTTCTGTTGGCCATGCCCCATCAAAGTTGCACTTAACCCAAG
ATTGTGGTGGAGCTTCCCATGTTTCTCGTCTGTCCCGACGGTGTTGTGGTTGGTGC
TTTCCTTACATTCTGAGCCTCTTTCCTTCTAATCCACTCATCTGCATCTTCTTGTGTC
CTTACTAATACCTCATTGGTTCCAAATTCCCTCCCTTTAAGCACCAGCTCGTTTCTG
TTCTTCCACAGCCTCCCAAGTATCCAAGGGACTAAAGCCTCCACATTCTTCAGATC
AGGATATTCTTGTTTAAGATGTTGAACTCTATGGAGGTTTGTATGAACTGATGATCT
AGGACCGGATAAGTTCCCTTCTTCATAGCGAACTTATTCAAAGAATGTTTTGTGTAT
CATTCTTGTTACATTGTTATTAATGAAAAAATATTATTGGTCATTGGACTGAACACGA
GTGTTAAATATGGACCAGGCCCCAAATAAGATCCATTGATATATGAATTAAATAACA
AGAATAAATCGAGTCACCAAACCACTTGCCTTTTTTAACGAGACTTGTTCACCAACT
TGATACAAAAGTCATTATCCTATGCAAATCAATAATCATACAAAAATATCCAATAACA
CTAAAAAATTAAAAGAAATGGATAATTTCACAATATGTTATACGATAAAGAAGTTACT
TTTCCAAGAAATTCACTGATTTTATAAGCCCACTTGCATTAGATAAATGGCAAAAAA
AAACAAAAAGGAAAAGAAATAAAGCACGAAGAATTCTAGAAAATACGAAATACGCTT
CAATGCAGTGGGACCCACGGTTCAATTATTGCCAATTTTCAGCTCCACCGTATATTT
AAAAAATAAAACGATAATGCTAAAAAAATATAAATCGTAACGATCGTTAAATCTCAAC
GGCTGGATCTTATGACGACCGTTAGAAATTGTGGTTGTCGACGAGTCAGTAATAAA
CGGCGTCAAAGTGGTTGCAGCCGGCACACACGAGTCGTGTTTATCAACTCAAAGC
ACAAATACTTTTCCTCAACCTAAAAATAAGGCAATTAGCCAAAAACAACTTTGCGTG
TAAACAACGCTCAATACACGTGTCATTTTATTATTAGCTATTGCTTCACCGCCTTAG
CTTTCTCGTGACCTAGTCGTCCTCGTCTTTTCTTCTTCTTCTTCTATAAAACAATACC
CAAAGAGCTCTTCTTCTTCACAATTCAGATTTCAATTTCTCAAAATCTTAAAAACTTT
CTCTCAATTCTCTCTACCGTGATCAAGGTAAATTTCTGTGTTCCTTATTCTCTCAAAA
TCTTCGATTTTGTTTTCGTTCGATCCCAATTTCGTATATGTTCTTTGGTTTAGATTCT
GTTAATCTTAGATCGAAGACGATTTTCTGGGTTTGATCGTTAGATATCATCTTAATTC
TCGATTAGGGTTTCATAGATATCATCCGATTTGTTCAAATAATTTGAGTTTTGTCGAA
TAATTACTCTTCGATTTGTGATTTCTATCTAGATCTGGTGTTAGTTTCTAGTTTGTGC
GATCGAATTTGTCGATTAATCTGAGTTTTTCTGATTAACAGACGCGTCCATGGTTGG
GTACCAACAAATGGCTGGTGGACTTAACGATATCTTCGAAGCTCAGAAGATTGAAT
GGCATGAGGATACTGGTGGATCTAGCATCCCCGGACTTGAGGTTCTTTTCCAAGG
ACCTCTCGAGCCTTATAAATGCCCTGAGTGTGGAAAATCTTTCTCTCAATCTGGAG

ATCTTAGGAGGCACCAAAGGACCCATACCGGTGAAAAACCTTATAAATGCCCTGAG
TGTGGAAAATCATTCAGTAGGAGTGATCATCTTACCACCCATCAGAGGACCCATAC
TGGTGAAAAACCTTATAAATGCCCTGAGTGTGGAAAATCATTCTCACAATCTTCATC
TCTTGTTAGGCATCAGAGGACTCATACCGGAGAAAAACCTTATAAATGCCCTGAGT
GTGGAAAATCATTCTCAACATCTGGAAACCTTGTTAGGCACCAAAGGACCCATACT
GGTGAAAAACCTTATAAATGCCCTGAGTGTGGAAAATCATTCTCTCAGAGAGCAAA
CCTTAGGGCTCATCAAAGGACACACACTGGAGAAAAACCTTATAAATGCCCTGAGT
GTGGAAAATCTTTCTCTAGGAGTGATAAGCTCACCGAGCATCAAAGGACTCACACC
GGACTCGAGGACTACAAAGACGATGACGACAAAGACTACAAAGACGATGACGACA
AAGACTACAAAGACGATGACGACAAAAAGCTTGATATCGCCATGGGGGGCGCGCC
AAGCTATCAAACAAGTTTGTACAAAAAAGCAGGCTCCGCGGCCGCCCCCTTCACCA
TGGGATCCCTGCCCACCTGCAGCTGTCTTGATCGAGTTATACAAAAAGACAAAGGC
CCATATTATACACACCTTGGGGCAGGACCAAGTGTTGCTGCTGTCAGGGAAATCAT
GGAGAATAGGTATGGTCAAAAAGGAAACGCAATAAGGATAGAAATAGTAGTGTACA
CCGGTAAAGAAGGGAAAAGCTCTCATGGGTGTCCAATTGCTAAGTGGGTTTTAAGA
AGAAGCAGTGATGAAGAAAAAGTTCTTTGTTTGGTCCGGCAGCGTACAGGCCACC
ACTGTCCAACTGCTGTGATGGTGGTGCTCATCATGGTGTGGGATGGCATCCCTCTT
CCAATGGCCGACCGGCTATACACAGAGCTCACAGAGAATCTAAAGTCATACAATGG
GCACCCTACCGACAGAAGATGCACCCTCAATGAAAATCGTACCTGTACATGTCAAG
GAATTGATCCAGAGACTTGTGGAGCTTCATTCTCTTTTGGCTGTTCATGGAGTATGT
ACTTTAATGGCTGTAAGTTTGGTAGAAGCCCAAGCCCCAGAAGATTTAGAATTGAT
CCAAGCTCTCCCTTACATGAAAAAAACCTTGAAGATAACTTACAGAGTTTGGCTACA
CGATTAGCTCCAATTTATAAGCAGTATGCTCCAGTAGCTTACCAAAATCAGGTGGA
ATATGAAAATGTTGCCCGAGAATGTCGGCTTGGCAGCAAGGAAGGTCGACCCTTC
TCTGGGGTCACTGCTTGCCTGGACTTCTGTGCTCATCCCCACAGGGACATTCACAA
CATGAATAATGGAAGCACTGTGGTTTGTACCTTAACTCGAGAAGATAACCGCTCTTT
GGGTGTTATTCCTCAAGATGAGCAGCTCCATGTGCTACCTCTTTATAAGCTTTCAGA
CACAGATGAGTTTGGCTCCAAGGAAGGAATGGAAGCCAAGATCAAATCTGGGGCC
ATCGAGGTCCTGGCACCCCGCCGCAAAAAAAGAACGTGTTTCACTCAGCCTGTTC
CCCGTTCTGGAAAGAAGAGGGCTGCGATGATGACAGAGGTTCTTGCACATAAGAT
AAGGGCAGTGGAAAAGAAACCTATTCCCCGAATCAAGCGGAAGAATAACTCAACAA
CAACAAACAACAGTAAGCCTTCGTCACTGCCAACCTTAGGGAGTAACACTGAGACC
GTGCAACCTGAAGTAAAAAGTGAAACCGAACCCCATTTTATCTTAAAAAGTTCAGAC
AACACTAAAACTTATTCGCTGATGCCATCCGCTCCTCACCCAGTGAAAGAGGCATC
TCCAGGCTTCTCCTGGTCCCCGAAGACTGCTTCAGCCACACCAGCTCCACTGAAG
AATGACGCAACAGCCTCATGCGGGTTTTCAGAAAGAAGCAGCACTCCCCACTGTA
CGATGCCTTCGGGAAGACTCAGTGGTGCCAATGCTGCAGCTGCTGATGGCCCTGG
CATTTCACAGCTTGGCGAAGTGGCTCCTCTCCCCACCCTGTCTGCTCCTGTGATGG
AGCCCCTCATTAATTCTGAGCCTTCCACTGGTGTGACTGAGCCGCTAACGCCTCAT
CAGCCAAACCACCAGCCCTCCTTCCTCACCTCTCCTCAAGACCTTGCCTCTTCTCC
AATGGAAGAAGATGAGCAGCATTCTGAAGCAGATGAGCCTCCATCAGACGAACCC
CTATCTGATGACCCCCTGTCACCTGCTGAGGAGAAATTGCCCCACATTGATGAGTA
TTGGTCAGACAGTGAGCACATCTTTTTGGATGCAAATATTGGTGGGGTGGCCATCG
CACCTGCTCACGGCTCGGTTTTGATTGAGTGTGCCCGGCGAGAGCTGCACGCTAC
CACTCCTGTTGAGCACCCCAACCGTAATCATCCAACCCGCCTCTCCCTTGTCTTTT
ACCAGCACAAAAACCTAAATAAGCCCCAACATGGTTTTGAACTAAACAAGATTAAGT

TTGAGGCTAAAGAAGCTAAGAATAAGAAAATGAAGGCCTCAGAGCAAAAAGACCAG
GCAGCTAATGAAGGTCCAGAACAGTCCTCTGAAGTAAATGAATTGAACCAAATTCC
TTCTCATAAAGCATTAACATTAACCCATGACAATGTTGTCACCGTGTCCCCTTATGC
TCTCACACACGTTGCGGGGCCCTATAACCATTGGGTCTGAAAGGGTGGGCGCGCC
GACCCAGCTTTCTTGTACAAAGTGGTTCGATAATTCCTTAATTAACTAGTTCTAGAG
CGGCCGCCACCGCGGTGGAGCTCCTAGGTGAGTCTAGAGAGTTAATTAAGACCCG
GGACTAGTCCCTAGAGTCCTGCTTTAATGAGATATGCGAGACGCCTATGATCGCAT
GATATTTGCTTTCAATTCTGTTGTGCACGTTGTAAAAAACCTGAGCATGTGTAGCTC
AGATCCTTACCGCCGGTTTCGGTTCATTCTAATGAATATATCACCCGTTACTATCGT
ATTTTTATGAATAATATTCTCCGTTCAATTTACTGATTGTACCCTACTACTTATATGTA
CAATATTAAAATGAAAACAATATATTGTGCTGAATAGGTTTATAGCGACATCTATGAT
AGAGCGCCACAATAACAAACAATTGCGTTTTATTATTACAAATCCAATTTTAAAAAAA
GCGGCAGAACCGGTCAAACCTAAAAGACTGATTACATAAATCTTATTCAAATTTCAA
AAGTGCCCCAGGGGCTAGTATCTACGACACACCGAGCGGCGAACTAATAACGCTC
ACTGAAGGGAACTCCGGTTCCCCGCCGGCGCGCATGGGTGAGATTCCTTGAAGTT
GAGTATTGGCCGTCCGCTCTACCGAAAGTTACGGGCACCATTCAACCCGGTCCAG
CACGGCGGCCGGGTAACCGACTTGCTGCCCCGAGAATTATGCAGCATTTTTTTGG
TGTATGTGGGCCCCAAATGAAGTGCAGGTCAAACCTTGACAGTGACGACAAATCGT
TGGGCGGGTCCAGGGCGAATTTTGCGACGAGCT

pUBQ10::ZF108_3xFlag_YPet
pUBQ10
BLRP
ZF108
3xFlag
YPet
OCS term

AGTCTAGCTCAACAGAGCTTTTAACCCAAATTGGTACAATAGAATACAACTTTAGAT
CATAATTCTCAAAAGAAAGAGATTCCTTAGCTATTCTATCTGCCACTCCATTTCCTTC
TCGGCTTGTATGCACAAGCATAAAATCCTCAAACTTGCTAAGTAGATACTTTATGTC
TTGGATAATTGGATTGAGACTTGACAAGCATAACTTTCATGTAACCAAAGACACAAG
TTGCTGAGAATCCACCTCAAAAATGATCTTCCTATAATTGAATCGGGATAATGACAG
CACAGCCCATCTAAGAGCCTCCACTTCTACTTCCAGCACGCTTCTTACTTTTACCAC
AGCTCTTGCACCTAACCATAACACCTTCCCTGTATGATCGCGAAGCACCCACCCTA
AGCCACATTTTAATCCTTCTGTTGGCCATGCCCCATCAAAGTTGCACTTAACCCAAG
ATTGTGGTGGAGCTTCCCATGTTTCTCGTCTGTCCCGACGGTGTTGTGGTTGGTGC
TTTCCTTACATTCTGAGCCTCTTTCCTTCTAATCCACTCATCTGCATCTTCTTGTGTC
CTTACTAATACCTCATTGGTTCCAAATTCCCTCCCTTTAAGCACCAGCTCGTTTCTG
TTCTTCCACAGCCTCCCAAGTATCCAAGGGACTAAAGCCTCCACATTCTTCAGATC
AGGATATTCTTGTTTAAGATGTTGAACTCTATGGAGGTTTGTATGAACTGATGATCT
AGGACCGGATAAGTTCCCTTCTTCATAGCGAACTTATTCAAAGAATGTTTTGTGTAT
CATTCTTGTTACATTGTTATTAATGAAAAAATATTATTGGTCATTGGACTGAACACGA
GTGTTAAATATGGACCAGGCCCCAAATAAGATCCATTGATATATGAATTAAATAACA
AGAATAAATCGAGTCACCAAACCACTTGCCTTTTTTAACGAGACTTGTTCACCAACT
TGATACAAAAGTCATTATCCTATGCAAATCAATAATCATACAAAAATATCCAATAACA

CTAAAAAATTAAAAGAAATGGATAATTTCACAATATGTTATACGATAAAGAAGTTACT
TTTCCAAGAAATTCACTGATTTTATAAGCCCACTTGCATTAGATAAATGGCAAAAAA
AAACAAAAAGGAAAAGAAATAAAGCACGAAGAATTCTAGAAAATACGAAATACGCTT
CAATGCAGTGGGACCCACGGTTCAATTATTGCCAATTTTCAGCTCCACCGTATATTT
AAAAAATAAAACGATAATGCTAAAAAAATATAAATCGTAACGATCGTTAAATCTCAAC
GGCTGGATCTTATGACGACCGTTAGAAATTGTGGTTGTCGACGAGTCAGTAATAAA
CGGCGTCAAAGTGGTTGCAGCCGGCACACACGAGTCGTGTTTATCAACTCAAAGC
ACAAATACTTTTCCTCAACCTAAAAATAAGGCAATTAGCCAAAAACAACTTTGCGTG
TAAACAACGCTCAATACACGTGTCATTTTATTATTAGCTATTGCTTCACCGCCTTAG
CTTTCTCGTGACCTAGTCGTCCTCGTCTTTTCTTCTTCTTCTTCTATAAAACAATACC
CAAAGAGCTCTTCTTCTTCACAATTCAGATTTCAATTTCTCAAAATCTTAAAAACTTT
CTCTCAATTCTCTCTACCGTGATCAAGGTAAATTTCTGTGTTCCTTATTCTCTCAAAA
TCTTCGATTTTGTTTTCGTTCGATCCCAATTTCGTATATGTTCTTTGGTTTAGATTCT
GTTAATCTTAGATCGAAGACGATTTTCTGGGTTTGATCGTTAGATATCATCTTAATTC
TCGATTAGGGTTTCATAGATATCATCCGATTTGTTCAAATAATTTGAGTTTTGTCGAA
TAATTACTCTTCGATTTGTGATTTCTATCTAGATCTGGTGTTAGTTTCTAGTTTGTGC
GATCGAATTTGTCGATTAATCTGAGTTTTTCTGATTAACAGACGCGTCCATGGTTGG
GTACCAACAAATGGCTGGTGGACTTAACGATATCTTCGAAGCTCAGAAGATTGAAT
GGCATGAGGATACTGGTGGATCTAGCATCCCCGGACTTGAGGTTCTTTTCCAAGG
ACCTCTCGAGCCTTATAAATGCCCTGAGTGTGGAAAATCTTTCTCTCAATCTGGAG
ATCTTAGGAGGCACCAAAGGACCCATACCGGTGAAAAACCTTATAAATGCCCTGAG
TGTGGAAAATCATTCAGTAGGAGTGATCATCTTACCACCCATCAGAGGACCCATAC
TGGTGAAAAACCTTATAAATGCCCTGAGTGTGGAAAATCATTCTCACAATCTTCATC
TCTTGTTAGGCATCAGAGGACTCATACCGGAGAAAAACCTTATAAATGCCCTGAGT
GTGGAAAATCATTCTCAACATCTGGAAACCTTGTTAGGCACCAAAGGACCCATACT
GGTGAAAAACCTTATAAATGCCCTGAGTGTGGAAAATCATTCTCTCAGAGAGCAAA
CCTTAGGGCTCATCAAAGGACACACACTGGAGAAAAACCTTATAAATGCCCTGAGT
GTGGAAAATCTTTCTCTAGGAGTGATAAGCTCACCGAGCATCAAAGGACTCACACC
GGACTCGAGGACTACAAAGACGATGACGACAAAGACTACAAAGACGATGACGACA
AAGACTACAAAGACGATGACGACAAAAAGCTTGATATCGCCATGGGGGGCGCGCC
AAGCTATCAAACAAGTTTGTACAAAAAAGCAGGCTCCGCGGCCGCCCCCTTCACC
CTAGACCCTTTGCCTGCTGCTGCTGCCGCTGTGAGCAAAGGCGAAGAGCTGTTCA
CCGGCGTGGTGCCCATCCTGGTGGAGCTGGACGGCGACGTGAACGGCCACAAGT
TCAGCGTGAGCGGCGAGGGCGAGGGCGACGCCACCTACGGCAAGCTGACCCTGA
AGCTGCTGTGCACCACCGGCAAGCTGCCCGTGCCCTGGCCCACCCTGGTGACCA
CCCTGGGCTACGGCGTGCAGTGCTTCGCCCGGTACCCCGACCACATGAAGCAGC
ACGACTTCTTCAAGAGCGCCATGCCCGAGGGCTACGTGCAGGAGCGGACCATCTT
CTTCAAGGACGACGGCAACTACAAGACCCGGGCCGAGGTGAAGTTCGAGGGCGA
CACCCTGGTGAACCGGATCGAGCTGAAGGGCATCGACTTCAAGGAGGACGGCAA
CATCCTGGGCCACAAGCTGGAGTACAACTACAACAGCCACAACGTGTACATCACC
GCCGACAAGCAGAAGAACGGCATCAAGGCCAACTTCAAGATCCGGCACAACATCG
AGGACGGCGGCGTGCAGCTGGCCGACCACTACCAGCAGAACACCCCCATCGGCG
ACGGCCCCGTGCTGCTGCCCGACAACCACTACCTGAGCTACCAGAGCGCCCTGTT
CAAGGACCCCAACGAGAAGCGGGACCACATGGTGCTGCTGGAGTTCCTGACCGC
CGCCGGCATCACCGAGGGCATGAACGAGCTCTATAATTAGAAGGGTGGGCGCGC
CGACCCAGCTTTCTTGTACAAAGTGGTTCGATAATTCCTTAATTAACTAGTTCTAGA

GCGGCCGCCACCGCGGTGGAGCTCCTAGGTGAGTCTAGAGAGTTAATTAAGACCC
GGGACTAGTCCCTAGAGTCCTGCTTTAATGAGATATGCGAGACGCCTATGATCGCA
TGATATTTGCTTTCAATTCTGTTGTGCACGTTGTAAAAAACCTGAGCATGTGTAGCT
CAGATCCTTACCGCCGGTTTCGGTTCATTCTAATGAATATATCACCCGTTACTATCG
TATTTTTATGAATAATATTCTCCGTTCAATTTACTGATTGTACCCTACTACTTATATGT
ACAATATTAAAATGAAAACAATATATTGTGCTGAATAGGTTTATAGCGACATCTATGA
TAGAGCGCCACAATAACAAACAATTGCGTTTTATTATTACAAATCCAATTTTAAAAAA
AGCGGCAGAACCGGTCAAACCTAAAAGACTGATTACATAAATCTTATTCAAATTTCA
AAAGTGCCCCAGGGGCTAGTATCTACGACACACCGAGCGGCGAACTAATAACGCT
CACTGAAGGGAACTCCGGTTCCCCGCCGGCGCGCATGGGTGAGATTCCTTGAAGT
TGAGTATTGGCCGTCCGCTCTACCGAAAGTTACGGGCACCATTCAACCCGGTCCA
GCACGGCGGCCGGGTAACCGACTTGCTGCCCCGAGAATTATGCAGCATTTTTTTG
GTGTATGTGGGCCCCAAATGAAGTGCAGGTCAAACCTTGACAGTGACGACAAATC
GTTGGGCGGGTCCAGGGCGAATTTTGCGACGAGCT

pUBQ10::ZF1CACTA1_3xFlag_TET1cd
pUBQ10
ZF1CACTA1
3xFlag
TET1cd
OCS term

AGTCTAGCTCAACAGAGCTTTTAACCCAAATTGGTACAATAGAATACAACTTTAGAT
CATAATTCTCAAAAGAAAGAGATTCCTTAGCTATTCTATCTGCCACTCCATTTCCTTC
TCGGCTTGTATGCACAAGCATAAAATCCTCAAACTTGCTAAGTAGATACTTTATGTC
TTGGATAATTGGATTGAGACTTGACAAGCATAACTTTCATGTAACCAAAGACACAAG
TTGCTGAGAATCCACCTCAAAAATGATCTTCCTATAATTGAATCGGGATAATGACAG
CACAGCCCATCTAAGAGCCTCCACTTCTACTTCCAGCACGCTTCTTACTTTTACCAC
AGCTCTTGCACCTAACCATAACACCTTCCCTGTATGATCGCGAAGCACCCACCCTA
AGCCACATTTTAATCCTTCTGTTGGCCATGCCCCATCAAAGTTGCACTTAACCCAAG
ATTGTGGTGGAGCTTCCCATGTTTCTCGTCTGTCCCGACGGTGTTGTGGTTGGTGC
TTTCCTTACATTCTGAGCCTCTTTCCTTCTAATCCACTCATCTGCATCTTCTTGTGTC
CTTACTAATACCTCATTGGTTCCAAATTCCCTCCCTTTAAGCACCAGCTCGTTTCTG
TTCTTCCACAGCCTCCCAAGTATCCAAGGGACTAAAGCCTCCACATTCTTCAGATC
AGGATATTCTTGTTTAAGATGTTGAACTCTATGGAGGTTTGTATGAACTGATGATCT
AGGACCGGATAAGTTCCCTTCTTCATAGCGAACTTATTCAAAGAATGTTTTGTGTAT
CATTCTTGTTACATTGTTATTAATGAAAAAATATTATTGGTCATTGGACTGAACACGA
GTGTTAAATATGGACCAGGCCCCAAATAAGATCCATTGATATATGAATTAAATAACA
AGAATAAATCGAGTCACCAAACCACTTGCCTTTTTTAACGAGACTTGTTCACCAACT
TGATACAAAAGTCATTATCCTATGCAAATCAATAATCATACAAAAATATCCAATAACA
CTAAAAAATTAAAAGAAATGGATAATTTCACAATATGTTATACGATAAAGAAGTTACT
TTTCCAAGAAATTCACTGATTTTATAAGCCCACTTGCATTAGATAAATGGCAAAAAA
AAACAAAAAGGAAAAGAAATAAAGCACGAAGAATTCTAGAAAATACGAAATACGCTT
CAATGCAGTGGGACCCACGGTTCAATTATTGCCAATTTTCAGCTCCACCGTATATTT
AAAAAATAAAACGATAATGCTAAAAAAATATAAATCGTAACGATCGTTAAATCTCAAC
GGCTGGATCTTATGACGACCGTTAGAAATTGTGGTTGTCGACGAGTCAGTAATAAA

CGGCGTCAAAGTGGTTGCAGCCGGCACACACGAGTCGTGTTTATCAACTCAAAGC
ACAAATACTTTTCCTCAACCTAAAAATAAGGCAATTAGCCAAAAACAACTTTGCGTG
TAAACAACGCTCAATACACGTGTCATTTTATTATTAGCTATTGCTTCACCGCCTTAG
CTTTCTCGTGACCTAGTCGTCCTCGTCTTTTCTTCTTCTTCTTCTATAAAACAATACC
CAAAGAGCTCTTCTTCTTCACAATTCAGATTTCAATTTCTCAAAATCTTAAAAACTTT
CTCTCAATTCTCTCTACCGTGATCAAGGTAAATTTCTGTGTTCCTTATTCTCTCAAAA
TCTTCGATTTTGTTTTCGTTCGATCCCAATTTCGTATATGTTCTTTGGTTTAGATTCT
GTTAATCTTAGATCGAAGACGATTTTCTGGGTTTGATCGTTAGATATCATCTTAATTC
TCGATTAGGGTTTCATAGATATCATCCGATTTGTTCAAATAATTTGAGTTTTGTCGAA
TAATTACTCTTCGATTTGTGATTTCTATCTAGATCTGGTGTTAGTTTCTAGTTTGTGC
GATCGAATTTGTCGATTAATCTGAGTTTTTCTGATTAACAGATGGTT GAG AAA CCT
TAC AAG TGC CCG GAG TGC GGA AAG TCT TTT TCA CAA AAC TCA ACC TTG
ACG GAG CAT CAG CGA ACA CAT ACC GGG GAA AAG CCG TAT AAG TGT CCG
GAA TGC GGG AAA TCT TTT TCT AGG TCT GAT AAC TTG GTG AGA CAC CAG
CGT ACA CAC ACA GGT GAA AAG CCT TAC AAG TGC CCA GAA TGC GGA AAG
AGC TTC AGC ACC ACC GGC AAT CTG ACC GTA CAT CAG CGA ACT CAT ACA
GGT GAG AAA CCC TAT AAG TGT CCT GAG TGC GGA AAA TCT TTC AGC CAA
TCA TCA AAT TTA GTG AGA CAT CAG AGG ACC CAC ACA GGG GAG AAG CCG
TAT AAA TGT CCC GAG TGT GGG AAA AGT TTC TCT CGA TCC GAT AAC CTG
GTA CGA CAT CAG CGA ACC CAT ACT GGT GAG AAG CCC TAT AAA TGC CCT
GAA TGC GGC AAA TCC TTT TCC ACG TCC GGG AGC CTA GTT AGG CAT CAA
CGA ACC CAT ACC GGT
AACCGCGCCAAGCTTGGTACCGACTACAAAGACGATGACGACAAAGACTACAAAG
ACGATGACGACAAAGACTACAAAGACGATGACGACAAAGGATCCCTTGAGGTTCTT
TTCGGCGCGCCAAGCTATCAAACAAGTTTGTACAAAAAAGCAGGCTCCGCGGCCG
CCCCCTTCACCATGGGATCCCTGCCCACCTGCAGCTGTCTTGATCGAGTTATACAA
AAAGACAAAGGCCCATATTATACACACCTTGGGGCAGGACCAAGTGTTGCTGCTGT
CAGGGAAATCATGGAGAATAGGTATGGTCAAAAAGGAAACGCAATAAGGATAGAAA
TAGTAGTGTACACCGGTAAAGAAGGGAAAAGCTCTCATGGGTGTCCAATTGCTAAG
TGGGTTTTAAGAAGAAGCAGTGATGAAGAAAAAGTTCTTTGTTTGGTCCGGCAGCG
TACAGGCCACCACTGTCCAACTGCTGTGATGGTGGTGCTCATCATGGTGTGGGAT
GGCATCCCTCTTCCAATGGCCGACCGGCTATACACAGAGCTCACAGAGAATCTAA
AGTCATACAATGGGCACCCTACCGACAGAAGATGCACCCTCAATGAAAATCGTACC
TGTACATGTCAAGGAATTGATCCAGAGACTTGTGGAGCTTCATTCTCTTTTGGCTGT
TCATGGAGTATGTACTTTAATGGCTGTAAGTTTGGTAGAAGCCCAAGCCCCAGAAG
ATTTAGAATTGATCCAAGCTCTCCCTTACATGAAAAAAACCTTGAAGATAACTTACA
GAGTTTGGCTACACGATTAGCTCCAATTTATAAGCAGTATGCTCCAGTAGCTTACC
AAAATCAGGTGGAATATGAAAATGTTGCCCGAGAATGTCGGCTTGGCAGCAAGGA
AGGTCGACCCTTCTCTGGGGTCACTGCTTGCCTGGACTTCTGTGCTCATCCCCACA
GGGACATTCACAACATGAATAATGGAAGCACTGTGGTTTGTACCTTAACTCGAGAA
GATAACCGCTCTTTGGGTGTTATTCCTCAAGATGAGCAGCTCCATGTGCTACCTCT
TTATAAGCTTTCAGACACAGATGAGTTTGGCTCCAAGGAAGGAATGGAAGCCAAGA
TCAAATCTGGGGCCATCGAGGTCCTGGCACCCCGCCGCAAAAAAAGAACGTGTTT
CACTCAGCCTGTTCCCCGTTCTGGAAAGAAGAGGGCTGCGATGATGACAGAGGTT
CTTGCACATAAGATAAGGGCAGTGGAAAAGAAACCTATTCCCCGAATCAAGCGGAA
GAATAACTCAACAACAACAAACAACAGTAAGCCTTCGTCACTGCCAACCTTAGGGA

GTAACACTGAGACCGTGCAACCTGAAGTAAAAAGTGAAACCGAACCCCATTTTATC
TTAAAAAGTTCAGACAACACTAAAACTTATTCGCTGATGCCATCCGCTCCTCACCCA
GTGAAAGAGGCATCTCCAGGCTTCTCCTGGTCCCCGAAGACTGCTTCAGCCACAC
CAGCTCCACTGAAGAATGACGCAACAGCCTCATGCGGGTTTTCAGAAAGAAGCAG
CACTCCCCACTGTACGATGCCTTCGGGAAGACTCAGTGGTGCCAATGCTGCAGCT
GCTGATGGCCCTGGCATTTCACAGCTTGGCGAAGTGGCTCCTCTCCCCACCCTGT
CTGCTCCTGTGATGGAGCCCCTCATTAATTCTGAGCCTTCCACTGGTGTGACTGAG
CCGCTAACGCCTCATCAGCCAAACCACCAGCCCTCCTTCCTCACCTCTCCTCAAGA
CCTTGCCTCTTCTCCAATGGAAGAAGATGAGCAGCATTCTGAAGCAGATGAGCCTC
CATCAGACGAACCCCTATCTGATGACCCCCTGTCACCTGCTGAGGAGAAATTGCC
CCACATTGATGAGTATTGGTCAGACAGTGAGCACATCTTTTTGGATGCAAATATTG
GTGGGGTGGCCATCGCACCTGCTCACGGCTCGGTTTTGATTGAGTGTGCCCGGC
GAGAGCTGCACGCTACCACTCCTGTTGAGCACCCCAACCGTAATCATCCAACCCG
CCTCTCCCTTGTCTTTTACCAGCACAAAAACCTAAATAAGCCCCAACATGGTTTTGA
ACTAAACAAGATTAAGTTTGAGGCTAAAGAAGCTAAGAATAAGAAAATGAAGGCCT
CAGAGCAAAAAGACCAGGCAGCTAATGAAGGTCCAGAACAGTCCTCTGAAGTAAA
TGAATTGAACCAAATTCCTTCTCATAAAGCATTAACATTAACCCATGACAATGTTGT
CACCGTGTCCCCTTATGCTCTCACACACGTTGCGGGGCCCTATAACCATTGGGTCT
GAAAGGGTGGGCGCGCCGACCCAGCTTTCTTGTACAAAGTGGTTCGATAATTCTTA
ATTAACTAGTTCTAGAGCGGCCGCCACCGCGGTGGAGCTCCTAGGTGAGTCTAGA
GAGTTAATTAAGACCCGGGACTAGTCCCTAGAGTCCTGCTTTAATGAGATATGCGA
GACGCCTATGATCGCATGATATTTGCTTTCAATTCTGTTGTGCACGTTGTAAAAAAC
CTGAGCATGTGTAGCTCAGATCCTTACCGCCGGTTTCGGTTCATTCTAATGAATATA
TCACCCGTTACTATCGTATTTTTATGAATAATATTCTCCGTTCAATTTACTGATTGTA
CCCTACTACTTATATGTACAATATTAAAATGAAAACAATATATTGTGCTGAATAGGTT
TATAGCGACATCTATGATAGAGCGCCACAATAACAAACAATTGCGTTTTATTATTAC
AAATCCAATTTTAAAAAAAGCGGCAGAACCGGTCAAACCTAAAAGACTGATTACATA
AATCTTATTCAAATTTCAAAAGTGCCCCAGGGGCTAGTATCTACGACACACCGAGC
GGCGAACTAATAACGCTCACTGAAGGGAACTCCGGTTCCCCGCCGGCGCGCATG
GGTGAGATTCCTTGAAGTTGAGTATTGGCCGTCCGCTCTACCGAAAGTTACGGGC
ACCATTCAACCCGGTCCAGCACGGCGGCCGGGTAACCGACTTGCTGCCCCGAGA
ATTATGCAGCATTTTTTTGGTGTATGTGGGCCCCAAATGAAGTGCAGGTCAAACCT
TGACAGTGACGACAAATCGTTGGGCGGGTCCAGGGCGAATTTTGCGACGAGCT

pUBQ10::ZF2CACTA1_3xFlag_TET1cd

pUBQ10
ZF2CACTA1
3xFlag
TET1cd
OCS term

AGTCTAGCTCAACAGAGCTTTTAACCCAAATTGGTACAATAGAATACAACTTTAGAT
CATAATTCTCAAAAGAAAGAGATTCCTTAGCTATTCTATCTGCCACTCCATTTCCTTC
TCGGCTTGTATGCACAAGCATAAAATCCTCAAACTTGCTAAGTAGATACTTTATGTC

TTGGATAATTGGATTGAGACTTGACAAGCATAACTTTCATGTAACCAAAGACACAAG
TTGCTGAGAATCCACCTCAAAAATGATCTTCCTATAATTGAATCGGGATAATGACAG
CACAGCCCATCTAAGAGCCTCCACTTCTACTTCCAGCACGCTTCTTACTTTTACCAC
AGCTCTTGCACCTAACCATAACACCTTCCCTGTATGATCGCGAAGCACCCACCCTA
AGCCACATTTTAATCCTTCTGTTGGCCATGCCCCATCAAAGTTGCACTTAACCCAAG
ATTGTGGTGGAGCTTCCCATGTTTCTCGTCTGTCCCGACGGTGTTGTGGTTGGTGC
TTTCCTTACATTCTGAGCCTCTTTCCTTCTAATCCACTCATCTGCATCTTCTTGTGTC
CTTACTAATACCTCATTGGTTCCAAATTCCCTCCCTTTAAGCACCAGCTCGTTTCTG
TTCTTCCACAGCCTCCCAAGTATCCAAGGGACTAAAGCCTCCACATTCTTCAGATC
AGGATATTCTTGTTTAAGATGTTGAACTCTATGGAGGTTTGTATGAACTGATGATCT
AGGACCGGATAAGTTCCCTTCTTCATAGCGAACTTATTCAAAGAATGTTTTGTGTAT
CATTCTTGTTACATTGTTATTAATGAAAAAATATTATTGGTCATTGGACTGAACACGA
GTGTTAAATATGGACCAGGCCCCAAATAAGATCCATTGATATATGAATTAAATAACA
AGAATAAATCGAGTCACCAAACCACTTGCCTTTTTTAACGAGACTTGTTCACCAACT
TGATACAAAAGTCATTATCCTATGCAAATCAATAATCATACAAAAATATCCAATAACA
CTAAAAAATTAAAAGAAATGGATAATTTCACAATATGTTATACGATAAAGAAGTTACT
TTTCCAAGAAATTCACTGATTTTATAAGCCCACTTGCATTAGATAAATGGCAAAAAA
AAACAAAAAGGAAAAGAAATAAAGCACGAAGAATTCTAGAAAATACGAAATACGCTT
CAATGCAGTGGGACCCACGGTTCAATTATTGCCAATTTTCAGCTCCACCGTATATTT
AAAAAATAAAACGATAATGCTAAAAAAATATAAATCGTAACGATCGTTAAATCTCAAC
GGCTGGATCTTATGACGACCGTTAGAAATTGTGGTTGTCGACGAGTCAGTAATAAA
CGGCGTCAAAGTGGTTGCAGCCGGCACACACGAGTCGTGTTTATCAACTCAAAGC
ACAAATACTTTTCCTCAACCTAAAAATAAGGCAATTAGCCAAAAACAACTTTGCGTG
TAAACAACGCTCAATACACGTGTCATTTTATTATTAGCTATTGCTTCACCGCCTTAG
CTTTCTCGTGACCTAGTCGTCCTCGTCTTTTCTTCTTCTTCTTCTATAAAACAATACC
CAAAGAGCTCTTCTTCTTCACAATTCAGATTTCAATTTCTCAAAATCTTAAAAACTTT
CTCTCAATTCTCTCTACCGTGATCAAGGTAAATTTCTGTGTTCCTTATTCTCTCAAAA
TCTTCGATTTTGTTTTCGTTCGATCCCAATTTCGTATATGTTCTTTGGTTTAGATTCT
GTTAATCTTAGATCGAAGACGATTTTCTGGGTTTGATCGTTAGATATCATCTTAATTC
TCGATTAGGGTTTCATAGATATCATCCGATTTGTTCAAATAATTTGAGTTTTGTCGAA
TAATTACTCTTCGATTTGTGATTTCTATCTAGATCTGGTGTTAGTTTCTAGTTTGTGC
GATCGAATTTGTCGATTAATCTGAGTTTTTCTGATTAACAGATGGTT GAG AAG CCC
TAT AAG TGC CCC GAG TGT GGG AAG TCT TTC AGC AGG GAA GAT AAC CTC
CAC ACT CAT CAA AGA ACG CAT ACG GGA GAA AAG CCT TAC AAA TGT CCG
GAA TGT GGG AAA TCA TTC AGC CAA TCA AGC AAT CTC GTG AGA CAT CAA
CGT ACA CAT ACA GGG GAA AAA CCA TAT AAG TGC CCC GAA TGC GGT AAG
TCA TTT TCA CAC CGT ACT ACT CTG ACA AAC CAT CAA CGT ACC CAT ACT
GGG GAG AAG CCT TAT AAA TGT CCT GAG TGT GGG AAG AGC TTC TCT CAG
AGG GCG CAT TTG GAG CGT CAC CAG AGG ACA CAT ACC GGC GAA AAA CCG
TAC AAA TGT CCT GAA TGT GGA AAG TCA TTC TCA CGT AGT GAC AAT CTC
GTC AGG CAC CAG CGT ACT CAT ACT GGA GAG AAA CCC TAC AAG TGT CCT
GAA TGT GGT AAG TCC TTC AGT CAA TCC TCC AGC CTA GTG AGA CAT CAA
AGG ACG CAT ACC GGG
AACCGCGCCAAGCTTGGTACCGACTACAAAGACGATGACGACAAAGACTACAAAG
ACGATGACGACAAAGACTACAAAGACGATGACGACAAAGGATCCCTTGAGGTTCTT
TTCGGCGCGCCAAGCTATCAAACAAGTTTGTACAAAAAAGCAGGCTCCGCGGCCG

CCCCCTTCACCATGGGATCCCTGCCCACCTGCAGCTGTCTTGATCGAGTTATACAA
AAAGACAAAGGCCCATATTATACACACCTTGGGGCAGGACCAAGTGTTGCTGCTGT
CAGGGAAATCATGGAGAATAGGTATGGTCAAAAAGGAAACGCAATAAGGATAGAAA
TAGTAGTGTACACCGGTAAAGAAGGGAAAAGCTCTCATGGGTGTCCAATTGCTAAG
TGGGTTTTAAGAAGAAGCAGTGATGAAGAAAAAGTTCTTTGTTTGGTCCGGCAGCG
TACAGGCCACCACTGTCCAACTGCTGTGATGGTGGTGCTCATCATGGTGTGGGAT
GGCATCCCTCTTCCAATGGCCGACCGGCTATACACAGAGCTCACAGAGAATCTAAA
GTCATACAATGGGCACCCTACCGACAGAAGATGCACCCTCAATGAAAATCGTACCT
GTACATGTCAAGGAATTGATCCAGAGACTTGTGGAGCTTCATTCTCTTTTGGCTGTT
CATGGAGTATGTACTTTAATGGCTGTAAGTTTGGTAGAAGCCCAAGCCCCAGAAGA
TTTAGAATTGATCCAAGCTCTCCCTTACATGAAAAAAACCTTGAAGATAACTTACAG
AGTTTGGCTACACGATTAGCTCCAATTTATAAGCAGTATGCTCCAGTAGCTTACCAA
AATCAGGTGGAATATGAAAATGTTGCCCGAGAATGTCGGCTTGGCAGCAAGGAAG
GTCGACCCTTCTCTGGGGTCACTGCTTGCCTGGACTTCTGTGCTCATCCCCACAG
GGACATTCACAACATGAATAATGGAAGCACTGTGGTTTGTACCTTAACTCGAGAAG
ATAACCGCTCTTTGGGTGTTATTCCTCAAGATGAGCAGCTCCATGTGCTACCTCTTT
ATAAGCTTTCAGACACAGATGAGTTTGGCTCCAAGGAAGGAATGGAAGCCAAGATC
AAATCTGGGGCCATCGAGGTCCTGGCACCCCGCCGCAAAAAAAGAACGTGTTTCA
CTCAGCCTGTTCCCCGTTCTGGAAAGAAGAGGGCTGCGATGATGACAGAGGTTCT
TGCACATAAGATAAGGGCAGTGGAAAAGAAACCTATTCCCCGAATCAAGCGGAAG
AATAACTCAACAACAACAAACAACAGTAAGCCTTCGTCACTGCCAACCTTAGGGAG
TAACACTGAGACCGTGCAACCTGAAGTAAAAAGTGAAACCGAACCCCATTTTATCT
TAAAAAGTTCAGACAACACTAAAACTTATTCGCTGATGCCATCCGCTCCTCACCCA
GTGAAAGAGGCATCTCCAGGCTTCTCCTGGTCCCCGAAGACTGCTTCAGCCACAC
CAGCTCCACTGAAGAATGACGCAACAGCCTCATGCGGGTTTTCAGAAAGAAGCAG
CACTCCCCACTGTACGATGCCTTCGGGAAGACTCAGTGGTGCCAATGCTGCAGCT
GCTGATGGCCCTGGCATTTCACAGCTTGGCGAAGTGGCTCCTCTCCCCACCCTGT
CTGCTCCTGTGATGGAGCCCCTCATTAATTCTGAGCCTTCCACTGGTGTGACTGAG
CCGCTAACGCCTCATCAGCCAAACCACCAGCCCTCCTTCCTCACCTCTCCTCAAGA
CCTTGCCTCTTCTCCAATGGAAGAAGATGAGCAGCATTCTGAAGCAGATGAGCCTC
CATCAGACGAACCCCTATCTGATGACCCCCTGTCACCTGCTGAGGAGAAATTGCC
CCACATTGATGAGTATTGGTCAGACAGTGAGCACATCTTTTTGGATGCAAATATTG
GTGGGGTGGCCATCGCACCTGCTCACGGCTCGGTTTTGATTGAGTGTGCCCGGC
GAGAGCTGCACGCTACCACTCCTGTTGAGCACCCCAACCGTAATCATCCAACCCG
CCTCTCCCTTGTCTTTTACCAGCACAAAAACCTAAATAAGCCCCAACATGGTTTTGA
ACTAAACAAGATTAAGTTTGAGGCTAAAGAAGCTAAGAATAAGAAAATGAAGGCCT
CAGAGCAAAAAGACCAGGCAGCTAATGAAGGTCCAGAACAGTCCTCTGAAGTAAAT
GAATTGAACCAAATTCCTTCTCATAAAGCATTAACATTAACCCATGACAATGTTGTC
ACCGTGTCCCCTTATGCTCTCACACACGTTGCGGGGCCCTATAACCATTGGGTCTG
AAAGGGTGGGCGCGCCGACCCAGCTTTCTTGTACAAAGTGGTTCGATAATTCTTAA
TTAACTAGTTCTAGAGCGGCCGCCACCGCGGTGGAGCTCCTAGGTGAGTCTAGAG
AGTTAATTAAGACCCGGGACTAGTCCCTAGAGTCCTGCTTTAATGAGATATGCGAG
ACGCCTATGATCGCATGATATTTGCTTTCAATTCTGTTGTGCACGTTGTAAAAAACC
TGAGCATGTGTAGCTCAGATCCTTACCGCCGGTTTCGGTTCATTCTAATGAATATAT
CACCCGTTACTATCGTATTTTTATGAATAATATTCTCCGTTCAATTTACTGATTGTAC
CCTACTACTTATATGTACAATATTAAAATGAAAACAATATATTGTGCTGAATAGGTTT

ATAGCGACATCTATGATAGAGCGCCACAATAACAAACAATTGCGTTTTATTATTACA
AATCCAATTTTAAAAAAAGCGGCAGAACCGGTCAAACCTAAAAGACTGATTACATAA
ATCTTATTCAAATTTCAAAAGTGCCCCAGGGGCTAGTATCTACGACACACCGAGCG
GCGAACTAATAACGCTCACTGAAGGGAACTCCGGTTCCCCGCCGGCGCGCATGG
GTGAGATTCCTTGAAGTTGAGTATTGGCCGTCCGCTCTACCGAAAGTTACGGGCAC
CATTCAACCCGGTCCAGCACGGCGGCCGGGTAACCGACTTGCTGCCCCGAGAATT
ATGCAGCATTTTTTTGGTGTATGTGGGCCCCAAATGAAGTGCAGGTCAAACCTTGA
CAGTGACGACAAATCGTTGGGCGGGTCCAGGGCGAATTTTGCGACGAGCT

SunTagFWAg4-22aa-TET1cd

gRNA FWAg4
U6
NOS term
TET1cd
2xNLS
Linker
1xHA
sfGFP
scFV
UBQ10
Insulator
Omega RBC
dCAS9
3xNLS
GCN4 10x 22aa
OCS term

AAAAAAAGCACCGACTCGGTGCCACTTTTTCAAGTTGATAACGGACTAGCCTTATTT
TAACTTGCTATTTCTAGCTCTAAAACAAGCCCATACATCTTTCCGTCAATCACTACTT
CGACTCTAGCTGTATATAAACTCAGCTTCGTTTTCTTATCTAAGCGATGTGGACTT
TTGAAGATTGTTTTCAACTTAAATGGGCCTATATAAGAAATACTATTGTTCTTTCCCA
TATAAATGGGCCTGCTTCTCTTCTTTCAGATTCCCAGGGGCCTTTTGAAGATTATCT
TCATATCTTAAGAATGAAGATGTTTTATTCAATCAAATTCTTGAAGGTTCGATGCCTA
ATCATTCTAATCCTGGGACAAACTATGAAACAAGATACAAAAACTCCGAATGGAAA
GTTAAAAAGAAGAAAACGAAAGCTACGGTTCAAGAAAATGTAAGCTGATAAACAAA
AAAAAACTGTATGAACGAAGAAGAAGAAAAAAAGCTAAGAAGAAATGATGTATTGT
GCGGAAGGCAAGTCGAGTTTCCGTTGTTCAACGAAAGCTTAAACGTAACATAGATG
ACACCGCGCGCGATAATTTATCCTAGTTTGCGCGCTATATTTTGTTTTCTATCGCGT
ATTAAATGTATAATTGCGGGACTCTAATCATAAAAACCCATCTCATAAATAACGTCA
TGCATTACATGTTAATTATTACATGCTTAACGTAATTCAACAGAAATTATATGATAAT
CATCGCAAGACCGGCAACAGGATTCAATCTTAAGAAACTTTATTGCCAAATGTTTGA
ACGATCGGGGAAATTCGAGCTCAAACTTACGGCCATGGCGTAGGCGGCCTTTTCC
GTTGTGATCTTCGCGGTCTCCTCCTTGTTTTTGGACTAGTACCACCACCGCTACCA
CCACCTTCGGTTACCGTGAAGGTTTTGGTAGCGTCGTCGTAGGTCCATTCACCGTC
AACACCGTTGTCGTTAGCGTACTGTTTGAAAACTTTTTCCGCGGTAGCAGCGTCAA
CAGCTTCGGTGGTGGTTTCACCTTTCAGGGTTTTACCGTTCAGGATAAGCTTGTAC

TCTTCGGTCCGAGATCCTCCTCCCGTACGTCAGACCCAATGGTTATAGGGCCCCG
CAACGTGTGTGAGAGCATAAGGGGACACGGTGACAACATTGTCATGGGTTAATGT
TAATGCTTTATGAGAAGGAATTTGGTTCAATTCATTTACTTCAGAGGACTGTTCTGG
ACCTTCATTAGCTGCCTGGTCTTTTTGCTCTGAGGCCTTCATTTTCTTATTCTTAGCT
TCTTTAGCCTCAAACTTAATCTTGTTTAGTTCAAAACCATGTTGGGGCTTATTTAGGT
TTTTGTGCTGGTAAAAGACAAGGGAGAGGCGGGTTGGATGATTACGGTTGGGGTG
CTCAACAGGAGTGGTAGCGTGCAGCTCTCGCCGGGCACACTCAATCAAAACCGAG
CCGTGAGCAGGTGCGATGGCCACCCCACCAATATTTGCATCCAAAAAGATGTGCT
CACTGTCTGACCAATACTCATCAATGTGGGGCAATTTCTCCTCAGCAGGTGACAGG
GGGTCATCAGATAGGGGTTCGTCTGATGGAGGCTCATCTGCTTCAGAATGCTGCT
CATCTTCTTCCATTGGAGAAGAGGCAAGGTCTTGAGGAGAGGTGAGGAAGGAGGG
CTGGTGGTTTGGCTGATGAGGCGTTAGCGGCTCAGTCACACCAGTGGAAGGCTCA
GAATTAATGAGGGGCTCCATCACAGGAGCAGACAGGGTGGGGAGAGGAGCCACT
TCGCCAAGCTGTGAAATGCCAGGGCCATCAGCAGCTGCAGCATTGGCACCACTGA
GTCTTCCCGAAGGCATCGTACAGTGGGGAGTGCTGCTTCTTTCTGAAAACCCGCAT
GAGGCTGTTGCGTCATTCTTCAGTGGAGCTGGTGTGGCTGAAGCAGTCTTCGGGG
ACCAGGAGAAGCCTGGAGATGCCTCTTTCACTGGGTGAGGAGCGGATGGCATCA
GCGAATAAGTTTTAGTGTTGTCTGAACTTTTTAAGATAAAATGGGGTTCGGTTTCAC
TTTTTACTTCAGGTTGCACGGTCTCAGTGTTACTCCCTAAGGTTGGCAGTGACGAA
GGCTTACTGTTGTTTGTTGTTGTTGAGTTATTCTTCCGCTTGATTCGGGGAATAGGT
TTCTTTTCCACTGCCCTTATCTTATGTGCAAGAACCTCTGTCATCATCGCAGCCCTC
TTCTTTCCAGAACGGGGAACAGGCTGAGTGAAACACGTTCTTTTTTTGCGGCGGG
GTGCCAGGACCTCGATGGCCCCAGATTTGATCTTGGCTTCCATTCCTTCCTTGGAG
CCAAACTCATCTGTGTCTGAAAGCTTATAAAGAGGTAGCACATGGAGCTGCTCATC
TTGAGGAATAACACCCAAAGAGCGGTTATCTTCTCGAGTTAAGGTACAAACCACAG
TGCTTCCATTATTCATGTTGTGAATGTCCCTGTGGGGATGAGCACAGAAGTCCAGG
CAAGCAGTGACCCCAGAGAAGGGTCGACCTTCCTTGCTGCCAAGCCGACATTCTC
GGGCAACATTTTCATATTCCACCTGATTTTGGTAAGCTACTGGAGCATACTGCTTAT
AAATTGGAGCTAATCGTGTAGCCAAACTCTGTAAGTTATCTTCAAGGTTTTTTTCAT
GTAAGGGAGAGCTTGGATCAATTCTAAATCTTCTGGGGCTTGGGCTTCTACCAAAC
TTACAGCCATTAAAGTACATACTCCATGAACAGCCAAAAGAGAATGAAGCTCCACA
AGTCTCTGGATCAATTCCTTGACATGTACAGGTACGATTTTCATTGAGGGTGCATCT
TCTGTCGGTAGGGTGCCCATTGTATGACTTTAGATTCTCTGTGAGCTCTGTGTATA
GCCGGTCGGCCATTGGAAGAGGGATGCCATCCCACACCATGATGAGCACCACCAT
CACAGCAGTTGGACAGTGGTGGCCTGTACGCTGCCGGACCAAACAAAGAACTTTT
TCTTCATCACTGCTTCTTCTTAAAACCCACTTAGCAATTGGACACCCATGAGAGCTT
TTCCCTTCTTTACCGGTGTACACTACTATTTCTATCCTTATTGCGTTTCCTTTTTGAC
CATACCTATTCTCCATGATTTCCCTGACAGCAGCAACACTTGGTCCTGCCCCAAGG
TGTGTATAATATGGGCCTTTGTCTTTTTGTATAACTCGATCAAGACAGCTGCAGGTG
GGCAGCCCGGATCCGCCACCGCCCACTTTCCGCTTTTTCTTAGGAGCTTCCACCTT
GCGTTTTTTCTTGGGAGCGCCGCTGCCCAGGCTGGCATAATCGGGCACGTCATAG
GGATACGTACGACCGGTCCGACCTCCACCTTTGTAGAGCTCATCCATGCCATGTGT
AATCCCAGCAGCAGTTACAAACTCAAGAAGGACCATGTGGTCACGCTTTTCGTTGG
GATCTTTCGAAAGGACAGATTGTGTCGACAGGTAATGGTTGTCTGGTAAAAGGACA
GGGCCATCGCCAATTGGAGTATTTTGTTGATAATGGTCTGCTAGTTGAACGGAACC
ATCTTCAACGTTGTGGCGAATTTTGAAGTTAGCTTTGATTCCATTCTTTTGTTTGTCT

GCCGTGATGTATACATTGTGTGAGTTAAAGTTGTACTCGAGTTTGTGTCCAAGAAT
GTTTCCATCTTCTTTAAAATCAATACCCTTTAACTCGATACGATTAACAAGGGTATCA
CCTTCAAACTTGACTTCAGCACGCGTCTTGTAGGTCCCGTCATCTTTGAAAGATATA
GTGCGTTCCTGTACATAACCTTCGGGCATGGCACTCTTGAAAAAGTCATGCCGTTT
CATGTGATCCGGATAACGGGAAAAGCATTGAACACCATAGGTCAGAGTAGTGACA
AGTGTTGGCCACGGAACAGGTAGTTTTCCAGTAGTGCAAATAAATTTAAGGGTGAG
TTTTCCGTTTGTAGCATCACCTTCACCCTCTCCACGGACAGAAAATTTGTGCCCATT
AACATCACCATCTAATTCAACAAGAATTGGGACAACTCCAGTGAAAAGTTCTTCTCC
TTTGCTACCGCTTCCACCTCCACCTGGATCCAAGCTTCCGCCGCCTCCTGAACCAC
CACCGCCACTACCTCCTCCCCCAGAACCTCCGCCTCCACCAGCGTAATCTGGAAC
ATCGTATGGGTAGCTGCTCACGGTCACCAGGGTGCCCTGGCCCCAGTAGTCGAAC
AGGCCGGTCACGCAGTAGTACAGGGCGGTGTCGTCGCTGCGCACCTTGCTCATCT
GCAGGTACACGGTGTTCTTGCCGTTGTCCTTGCTGATGATGAAGCGGTCCTTCAG
GGCGCTGTTGTAGTCGGTGATGCCGTCGCCCCAGATCACGCCGATCCACTCCAGG
CCGCGGCCGGGGGCCTGGCGCACCCAGTTCACGCCGTAGTCGGTCAGGCTGAA
GCCGCTCACGGCGCAGCTCAGCTTCAGGCTGCCGCCGGGCTGCACCAGGCCGCC
GCCGCTCTCCAGCAGCTTCACCTCGCTGCCGCCGCCGCTGCTGCCGCCGCCGCC
GCTGCCGCCGCCGCCGCTGCCGCCGCCGCCGCGCTTCAGCTCCACCTTGGTGCC
CTGGCCGAACACCCAGTGGTTGCTGTACCACAGGGCGCAGAAGTAGGTGGCGAA
GTCCTCGGGCTGCAGGCTGCTGATGGTCAGGGTGGCCTTGTCGCCGATCAGGCT
GCCGCTGAAGCGGCTGGGCACGCCGGGGGCGCGGTTGTTGGTGCCGCCGATCA
GGCCCTTGAACAGCTTGCCGGGCTTCTCCTGCACCCAGCTGGCGTAGTTGCTGGT
GGTCACGGCGCCGGTGCTGCTGCGGCAGGTGATGGTCACGCGGTCGCCCACGCT
GGCGCTCAGGCTGCTGGGGCTCTGGGTCATCACGATGTCGGGGCCCATAAACCC
TAGGTGTTAATCAGAAAAACTCAGATTAATCGACAAATTCGATCGCACAAACTAGAA
ACTAACACCAGATCTAGATAGAAATCACAAATCGAAGAGTAATTATTCGACAAAACT
CAAATTATTTGAACAAATCGGATGATATCTATGAAACCCTAATCGAGAATTAAGATG
ATATCTAACGATCAAACCCAGAAAATCGTCTTCGATCTAAGATTAACAGAATCTAAA
CCAAAGAACATATACGAAATTGGGATCGAACGAAAACAAAATCGAAGATTTTGAGA
GAATAAGGAACACAGAAATTTACCTTGATCACGGTAGAGAGAATTGAGAGAAAGTT
TTTAAGATTTTGAGAAATTGAAATCTGAATTGTGAAGAAGAAGAGCTCTTTGGGTAT
TGTTTTATAGAAGAAGAAGAAGAAAAGACGAGGACGACTAGGTCACGAGAAAGCTA
AGGCGGTGAAGCAATAGCTAATAATAAAATGACACGTGTATTGAGCGTTGTTTACA
CGCAAAGTTGTTTTTGGCTAATTGCCTTATTTTTAGGTTGAGGAAAAGTATTTGTGC
TTTGAGTTGATAAACACGACTCGTGTGTGCCGGCTGCAACCACTTTGACGCCGTTT
ATTACTGACTCGTCGACAACCACAATTTCTAACGGTCGTCATAAGATCCAGCCGTT
GAGATTTAACGATCGTTACGATTTATATTTTTTTAGCATTATCGTTTTATTTTTTAAAT
ATACGGTGGAGCTGAAAATTGGCAATAATTGAACCGTGGGTCCCACTGCATTGAAG
CGTATTTCGTATTTTCTAGAATTCTTCGTGCTTTATTTCTTTTCCTTTTTGTTTTTTTTT
GCCATTTATCTAATGCAAGTGGGCTTATAAAATCAGTGAATTTCTTGGAAAAGTAAC
TTCTTTATCGTATAACATATTGTGAAATTATCCATTTCTTTTAATTTTTTAGTGTTATT
GGATATTTTTGTATGATTATTGATTTGCATAGGATAATGACTTTTGTATCAAGTTGGT
GAACAAGTCTCGTTAAAAAAGGCAAGTGGTTTGGTGACTCGATTTATTCTTGTTATT
TAATTCATATATCAATGGATCTTATTTGGGGCCTGGTCCATATTTAACACTCGTGTT
CAGTCCAATGACCAATAATATTTTTTCATTAATAACAATGTAACAAGAATGATACACA
AAACATTCTTTGAATAAGTTCGCTATGAAGAAGGGAACTTATCCGGTCCTAGATCAT

CAGTTCATACAAACCTCCATAGAGTTCAACATCTTAAACAAGAATATCCTGATCTGA
AGAATGTGGAGGCTTTAGTCCCTTGGATACTTGGGAGGCTGTGGAAGAACAGAAA
CGAGCTGGTGCTTAAAGGGAGGGAATTTGGAACCAATGAGGTATTAGTAAGGACA
CAAGAAGATGCAGATGAGTGGATTAGAAGGAAAGAGGCTCAGAATGTAAGGAAAG
CACCAACCACAACACCGTCGGGACAGACGAGAAACATGGGAAGCTCCACCACAAT
CTTGGGTTAAGTGCAACTTTGATGGGGCATGGCCAACAGAAGGATTAAAATGTGG
CTTAGGGTGGGTGCTTCGCGATCATACAGGGAAGGTGTTATGGTTAGGTGCAAGA
GCTGTGGTAAAAGTAAGAAGCGTGCTGGAAGTAGAAGTGGAGGCTCTTAGATGGG
CTGTGCTGTCATTATCCCGATTCAATTATAGGAAGATCATTTTTGAGGTGGATTCTC
AGCAACTTGTGTCTTTGGTTACATGAAAGTTATGCTTGTCAAGTCTCAATCCAATTA
TCCAAGACATAAAGTATCTACTTAGCAAGTTTGAGGATTTTATGCTTGTGCATACAA
GCCGAGAAGGAAATGGAGTGGCAGATAGAATAGCTAAGGAATCTCTTTCTTTTGAG
AATTATGATCTAAAGTTGTATTCTATTGTACCAATTTGGGTTAAAAGCTCTGTTGAGC
TAGACTCATATGCATCCTAGGAAACAAGTTGTAATGAGTTGCTGGCCTCTCTTGGG
ATTGTTCATCAAAGTAGCTGTCCCTACACACCACAACAGAATGGTGTTGTGGAGAG
AAAGCACAGACACATCCTTGAGATGGCAAGGGCACTTAAGTTTCAGAGTGGTGTAC
CTACCAGGTTTTGGGGAGACTGTGTCAAGACTGTTGTATACCTGATCAATAGACTC
CCTACTCCAATCTTACAAGGCAAATGTCCATATGAACTACTCTATCAGAAACATGCC
AAGCTTGATCACTTGAGAGTGTTTGGGTGTCTTGCTTTTGCAGCAACACTGCCCAA
AGGTGACAAACTTGCACCTAGAGCCAAGCGAACCATCTTCATTGGTTATTCTGAGA
CTCAAAAGGGTTACAGGTTGTATGACTTGGATAATAAGGTGATCATAGTGAGCAGG
GATGTAGTTTTCAGAGAGTTTCAGTTCCCTTTCAAAGAGTCCATCTCCCATGAACCT
GATATGTTCACTCGAGCAGGTTCACTTAGCTCTGAAGATACAGCTTTGCAGCTAGT
GTATAATGATATTTTTCCTTCCAGCTTTCATACTGATGATGATGATACTCCAGTTCTT
ATTCCTGACATTGTAGAGGCCAATGAGGATATCACTTTGGGGACTCATTCTACCCA
AAATCATATCACTTCAGCTGATGCTGCACCTTCAGGCCACCTGCATGCTCCTGCAG
AGTCAGTTGAGTCAGCTGAGCCTGACCTAGCAAATGCTGAATTTGAGCACCATACT
GCTGTAGCTGATCCCTCTCTAGTTCCAGCAAACCTGCACACTAGACCTAAACGCAA
TGCAGGTCCTCCCATCTGGCTCAAGGACTTTGTGACACTGAACAAAGGCTCTAGG
GATGTTCCATACCCTATAGCCAACTATGTTTCCTATGATCACTTGCCTGTTCACTAT
CAAGCTTATTTGAGTGCTTTCTCCACTTATACTGAACCTAAATCCTTCAAAGAAGCA
GCTCAAGATGAGAAATGGATGGAGGCCATGTCCCTTGAGATACAGGCTCTTGAGG
ATAATAACACCTGGGAGATTGTCCCTTTACCCCCTGGTAAACAGCCTATAGGGTCC
AAATGGGTGTACAAAATTAAATACAAAGCTAATGGTGAAGTTGACAGGTTTAAGGC
AAGGCTAGTGGCCAAGGGATACACTCAGCAAGAAGGCCTTGACTACCATGAAACT
TTTTCTCCAGTGGCCAAAATGGTCACTGTAAAAGCTGTCATATCTGTTGCTGCTTCC
AAGGGCTGGTTCCTTTTCCAGATGGATGTCAACAATGCTTTTCTACAAGGTGACCT
CATGGAAGAAGTTTATATGTCTCTGCCTCAGGGTTTTCACAGCCAAGGGGAGACCA
AAGTGTGCAGGCTGTTGAATCCCTCTATGGTTTAAAGCAAGCATCAAGGCAGTGGA
ACATCAAGCTTACCACTGTCCTTATGCAGGCTGGTTTTATGCAAAGTGCTTATGATC
ACTCCTTGTTTACCAAAAGAGAGGGGACTGACCTTGTCATAATCCTGATTTATGTG
GATGATCTGTTAATAACAGGCAGCAGCAATGTTTTGATTTCAGAAGCAAAGGCAAC
CCTGCATCAGCATTTCAAGATGAAAGATTTGGGAGAACTAAAATACTTCTTAGGCAT
TGAGGTGCTGAGATCAGAAAAGGGAATCCTACTGAACCAGAGGAAGTATGCACTG
GAACTGATATCAGGTGTTGGTCTGGGAGGCTGCAGACCAGTGTCAACCCCAATGG
AACAGAACCAAAGGCTGACTACTGTAGAATATGACAAGCACTTAGGAAAGACTGAT

GATGCAGAGTTAGAGGATGTGGGTTCCTATCAGAGACTGGTGGGAAAGCTTCTCT
ACTTGACAATCACAAGGCCAGATATCAGCTTTGCAGTGCAGGTGCTGAGTCAATTC
ATGCAGCAACCCAAACAGTCACACCTGGAAGCAGCATTGAGGGTTGTGAAGTACA
TAAAAGGTTCTCCAGGTGTTAGGAAGCGGCCGCATGCATATGAGTCTAGCTCAACA
GAGCTTTTAACCCAAATTGGTACAATAGAATACAACTTTAGATCATAATTCTCAAAA
GAAAGAGATTCCTTAGCTATTCTATCTGCCACTCCATTTCCTTCTCGGCTTGTATGC
ACAAGCATAAAATCCTCAAACTTGCTAAGTAGATACTTTATGTCTTGGATAATTGGA
TTGAGACTTGACAAGCATAACTTTCATGTAACCAAAGACACAAGTTGCTGAGAATC
CACCTCAAAAATGATCTTCCTATAATTGAATCGGGATAATGACAGCACAGCCCATCT
AAGAGCCTCCACTTCTACTTCCAGCACGCTTCTTACTTTTACCACAGCTCTTGCACC
TAACCATAACACCTTCCCTGTATGATCGCGAAGCACCCACCCTAAGCCACATTTTA
ATCCTTCTGTTGGCCATGCCCCATCAAAGTTGCACTTAACCCAAGATTGTGGTGGA
GCTTCCCATGTTTCTCGTCTGTCCCGACGGTGTTGTGGTTGGTGCTTTCCTTACAT
TCTGAGCCTCTTTCCTTCTAATCCACTCATCTGCATCTTCTTGTGTCCTTACTAATAC
CTCATTGGTTCCAAATTCCCTCCCTTTAAGCACCAGCTCGTTTCTGTTCTTCCACAG
CCTCCCAAGTATCCAAGGGACTAAAGCCTCCACATTCTTCAGATCAGGATATTCTT
GTTTAAGATGTTGAACTCTATGGAGGTTTGTATGAACTGATGATCTAGGACCGGAT
AAGTTCCCTTCTTCATAGCGAACTTATTCAAAGAATGTTTTGTGTATCATTCTTGTTA
CATTGTTATTAATGAAAAAATATTATTGGTCATTGGACTGAACACGAGTGTTAAATAT
GGACCAGGCCCCAAATAAGATCCATTGATATATGAATTAAATAACAAGAATAAATCG
AGTCACCAAACCACTTGCCTTTTTTAACGAGACTTGTTCACCAACTTGATACAAAAG
TCATTATCCTATGCAAATCAATAATCATACAAAAATATCCAATAACACTAAAAAATTA
AAAGAAATGGATAATTTCACAATATGTTATACGATAAAGAAGTTACTTTTCCAAGAAA
TTCACTGATTTTATAAGCCCACTTGCATTAGATAAATGGCAAAAAAAAACAAAAAGG
AAAAGAAATAAAGCACGAAGAATTCTAGAAAATACGAAATACGCTTCAATGCAGTG
GGACCCACGGTTCAATTATTGCCAATTTTCAGCTCCACCGTATATTTAAAAAATAAA
ACGATAATGCTAAAAAAATATAAATCGTAACGATCGTTAAATCTCAACGGCTGGATC
TTATGACGACCGTTAGAAATTGTGGTTGTCGACGAGTCAGTAATAAACGGCGTCAA
AGTGGTTGCAGCCGGCACACACGAGTCGTGTTTATCAACTCAAAGCACAAATACTT
TTCCTCAACCTAAAAATAAGGCAATTAGCCAAAAACAACTTTGCGTGTAAACAACGC
TCAATACACGTGTCATTTTATTATTAGCTATTGCTTCACCGCCTTAGCTTTCTCGTGA
CCTAGTCGTCCTCGTCTTTTCTTCTTCTTCTTCTATAAAACAATACCCAAAGAGCTCT
TCTTCTTCACAATTCAGATTTCAATTTCTCAAAATCTTAAAAACTTTCTCTCAATTCTC
TCTACCGTGATCAAGGTAAATTTCTGTGTTCCTTATTCTCTCAAAATCTTCGATTTTG
TTTTCGTTCGATCCCAATTTCGTATATGTTCTTTGGTTTAGATTCTGTTAATCTTAGA
TCGAAGACGATTTTCTGGGTTTGATCGTTAGATATCATCTTAATTCTCGATTAGGGT
TTCATAGATATCATCCGATTTGTTCAAATAATTTGAGTTTTGTCGAATAATTACTCTT
CGATTTGTGATTTCTATCTAGATCTGGTGTTAGTTTCTAGTTTGTGCGATCGAATTT
GTCGATTAATCTGAGTTTTTCTGATTAACAGGGATCATCAACAAGTTTGTACAAAAA
AGCAGGCTCTTTAAAGTATTTTTACAACAATTACCAACAACAACAAACAACAAACAA
CATTACAATTACTATTTACAATTACAAAAAAAGTTAACATGGACAAGAAGTACAGCA
TCGGCCTGGCCATCGGCACCAACTCTGTGGGCTGGGCCGTGATCACCGACGAGT
ACAAGGTGCCCAGCAAGAAATTCAAGGTGCTGGGCAACACCGACCGGCACAGCAT
CAAGAAGAACCTGATCGGCGCCCTGCTGTTCGACAGCGGAGAAACAGCCGAGGC
CACCCGGCTGAAGAGAACCGCCAGAAGAAGATACACCAGACGGAAGAACCGGAT
CTGCTATCTGCAAGAGATCTTCAGCAACGAGATGGCCAAGGTGGACGACAGCTTC

TTCCACAGACTGGAAGAGTCCTTCCTGGTGGAAGAGGATAAGAAGCACGAGCGGC
ACCCCATCTTCGGCAACATCGTGGACGAGGTGGCCTACCACGAGAAGTACCCCAC
CATCTACCACCTGAGAAAGAAACTGGTGGACAGCACCGACAAGGCCGACCTGCGG
CTGATCTATCTGGCCCTGGCCCACATGATCAAGTTCCGGGGCCACTTCCTGATCG
AGGGCGACCTGAACCCCGACAACAGCGACGTGGACAAGCTGTTCATCCAGCTGGT
GCAGACCTACAACCAGCTGTTCGAGGAAAACCCCATCAACGCCAGCGGCGTGGAC
GCCAAGGCCATCCTGTCTGCCAGACTGAGCAAGAGCAGACGGCTGGAAAATCTGA
TCGCCCAGCTGCCCGGCGAGAAGAAGAATGGCCTGTTCGGCAACCTGATTGCCCT
GAGCCTGGGCCTGACCCCCAACTTCAAGAGCAACTTCGACCTGGCCGAGGATGC
CAAACTGCAGCTGAGCAAGGACACCTACGACGACGACCTGGACAACCTGCTGGCC
CAGATCGGCGACCAGTACGCCGACCTGTTTCTGGCCGCCAAGAACCTGTCCGACG
CCATCCTGCTGAGCGACATCCTGAGAGTGAACACCGAGATCACCAAGGCCCCCCT
GAGCGCCTCTATGATCAAGAGATACGACGAGCACCACCAGGACCTGACCCTGCTG
AAAGCTCTCGTGCGGCAGCAGCTGCCTGAGAAGTACAAAGAGATTTTCTTCGACC
AGAGCAAGAACGGCTACGCCGGCTACATCGATGGCGGAGCCAGCCAGGAAGAGT
TCTACAAGTTCATCAAGCCCATCCTGGAAAAGATGGACGGCACCGAGGAACTGCT
CGTGAAGCTGAACAGAGAGGACCTGCTGCGGAAGCAGCGGACCTTCGACAACGG
CAGCATCCCCCACCAGATCCACCTGGGAGAGCTGCACGCCATTCTGCGGCGGCA
GGAAGATTTTTACCCATTCCTGAAGGACAACCGGGAAAAGATCGAGAAGATCCTGA
CCTTCCGCATCCCCTACTACGTGGGCCCTCTGGCCAGGGGAAACAGCAGATTCGC
CTGGATGACCAGAAAGAGCGAGGAAACCATCACCCCCTGGAACTTCGAGGAAGTG
GTGGACAAGGGCGCCAGCGCCCAGAGCTTCATCGAGCGGATGACCAACTTCGAT
AAGAACCTGCCCAACGAGAAGGTGCTGCCCAAGCACAGCCTGCTGTACGAGTACT
TCACCGTGTACAACGAGCTGACCAAAGTGAAATACGTGACCGAGGGAATGAGAAA
GCCCGCCTTCCTGAGCGGCGAGCAGAAAAAAGCCATCGTGGACCTGCTGTTCAAG
ACCAACCGGAAAGTGACCGTGAAGCAGCTGAAAGAGGACTACTTCAAGAAAATCG
AGTGCTTCGACTCCGTGGAAATCTCCGGCGTGGAAGATCGGTTCAACGCCTCCCT
GGGCACATACCACGATCTGCTGAAAATTATCAAGGACAAGGACTTCCTGGACAATG
AGGAAAACGAGGACATTCTGGAAGATATCGTGCTGACCCTGACACTGTTTGAGGA
CAGAGAGATGATCGAGGAACGGCTGAAAACCTATGCCCACCTGTTCGACGACAAA
GTGATGAAGCAGCTGAAGCGGCGGAGATACACCGGCTGGGGCAGGCTGAGCCG
GAAGCTGATCAACGGCATCCGGGACAAGCAGTCCGGCAAGACAATCCTGGATTTC
CTGAAGTCCGACGGCTTCGCCAACAGAAACTTCATGCAGCTGATCCACGACGACA
GCCTGACCTTTAAAGAGGACATCCAGAAAGCCCAGGTGTCCGGCCAGGGCGATAG
CCTGCACGAGCACATTGCCAATCTGGCCGGCAGCCCCGCCATTAAGAAGGGCATC
CTGCAGACAGTGAAGGTGGTGGACGAGCTCGTGAAAGTGATGGGCCGGCACAAG
CCCGAGAACATCGTGATCGAAATGGCCAGAGAGAACCAGACCACCCAGAAGGGA
CAGAAGAACAGCCGCGAGAGAATGAAGCGGATCGAAGAGGGCATCAAAGAGCTG
GGCAGCCAGATCCTGAAAGAACACCCCGTGGAAAACACCCAGCTGCAGAACGAGA
AGCTGTACCTGTACTACCTGCAGAATGGGCGGGATATGTACGTGGACCAGGAACT
GGACATCAACCGGCTGTCCGACTACGATGTGGACGCTATCGTGCCTCAGAGCTTT
CTGAAGGACGACTCCATCGATAACAAAGTGCTGACTCGGAGCGACAAGAACCGGG
GCAAGAGCGACAACGTGCCCTCCGAAGAGGTCGTGAAGAAGATGAAGAACTACTG
GCGCCAGCTGCTGAATGCCAAGCTGATTACCCAGAGGAAGTTCGACAATCTGACC
AAGGCCGAGAGAGGCGGCCTGAGCGAACTGGATAAGGCCGGCTTCATCAAGAGA
CAGCTGGTGGAAACCCGGCAGATCACAAAGCACGTGGCACAGATCCTGGACTCCC

GGATGAACACTAAGTACGACGAGAACGACAAACTGATCCGGGAAGTGAAAGTGAT
CACCCTGAAGTCCAAGCTGGTGTCCGATTTCCGGAAGGATTTCCAGTTTTACAAAG
TGCGCGAGATCAACAACTACCACCACGCCCACGACGCCTACCTGAACGCCGTCGT
GGGAACCGCCCTGATCAAAAAGTACCCTAAGCTGGAAAGCGAGTTCGTGTACGGC
GACTACAAGGTGTACGACGTGCGGAAGATGATCGCCAAGAGCGAGCAGGAAATC
GGCAAGGCTACCGCCAAGTACTTCTTCTACAGCAACATCATGAACTTTTTCAAGAC
CGAGATTACCCTGGCCAACGGCGAGATCCGGAAGCGGCCTCTGATCGAGACAAA
CGGCGAAACAGGCGAGATCGTGTGGGATAAGGGCCGGGACTTTGCCACCGTGCG
GAAAGTGCTGTCTATGCCCCAAGTGAATATCGTGAAAAAGACCGAGGTGCAGACA
GGCGGCTTCAGCAAAGAGTCTATCCTGCCCAAGAGGAACAGCGACAAGCTGATCG
CCAGAAAGAAGGACTGGGACCCTAAGAAGTACGGCGGCTTCGACAGCCCCACCG
TGGCCTATTCTGTGCTGGTGGTGGCCAAAGTGGAAAAGGGCAAGTCCAAGAAACT
GAAGAGTGTGAAAGAGCTGCTGGGGATCACCATCATGGAAAGAAGCAGCTTCGAG
AAGAATCCCATCGACTTTCTGGAAGCCAAGGGCTACAAAGAAGTGAAAAAGGACCT
GATCATCAAGCTGCCTAAGTACTCCCTGTTCGAGCTGGAAAACGGCCGGAAGAGA
ATGCTGGCCTCTGCCGGCGAACTGCAGAAGGGAAACGAACTGGCCCTGCCCTCC
AAATATGTGAACTTCCTGTACCTGGCCAGCCACTATGAGAAGCTGAAGGGCTCCC
CCGAGGATAATGAGCAGAAACAGCTGTTTGTGGAACAGCACAAACACTACCTGGA
CGAGATCATCGAGCAGATCAGCGAGTTCTCCAAGAGAGTGATCCTGGCCGACGCT
AATCTGGACAAGGTGCTGAGCGCCTACAACAAGCACAGAGACAAGCCTATCAGAG
AGCAGGCCGAGAATATCATCCACCTGTTTACCCTGACCAATCTGGGAGCCCCTGC
CGCCTTCAAGTACTTTGACACCACCATCGACCGGAAGAGGTACACCAGCACCAAA
GAGGTGCTGGACGCCACCCTGATCCACCAGAGCATCACCGGCCTGTACGAGACA
CGGATCGACCTGTCTCAGCTGGGAGGCGACGCCTATCCCTATGACGTGCCCGATT
ATGCCAGCCTGGGCAGCGGCTCCCCCAAGAAAAAACGCAAGGTGGAAGATCCTAA
GAAAAAGCGGAAAGTGGAAGATGCTCCAAAGAAGAAGAGAAAGGTCGACGGCATT
GGTAGTGGGAGCAACGGCAGCAGCGGATCCAACGGTCCGACTGACGCCGCGGAA
GAAGAACTTTTGAGCAAGAATTATCATCTTGAGAACGAAGTGGCTCGTCTTAAGAA
AGGTTCTGGCAGTGGAGGTTCTGGCTCCGGATCTGGTGGTTCGGGCTCAGGCGG
GTCCGGATCAGGCGAAGAACTGCTTTCAAAGAATTACCACCTGGAAAATGAGGTA
GCTAGACTGAAAAAGGGGAGCGGAAGTGGGGGCTCCGGGTCGGGCTCAGGGGG
CTCCGGTTCGGGAGGCTCAGGGTCGGGGGAGGAGTTGCTGAGCAAAAATTATCAT
TTGGAGAACGAAGTAGCACGACTAAAGAAAGGGTCCGGATCGGGTGGTTCAGGAT
CTGGATCCGGAGGATCAGGGTCCGGTGGGTCGGGCTCAGGAGAGGAGTTACTCT
CGAAAAATTATCATCTCGAAAACGAAGTGGCTCGGCTAAAAAAGGGCAGTGGTTCT
GGAGGATCTGGGTCGGGGTCAGGCGGGTCTGGATCTGGGGGATCTGGATCTGGT
GAAGAGCTATTATCTAAAAACTACCACCTCGAAAATGAGGTGGCACGCTTAAAAAA
GGGAAGTGGCAGTGGTGGGTCGGGATCTGGCTCTGGTGGCTCAGGCTCGGGAGG
TTCAGGTTCCGGGGAAGAGCTACTATCCAAGAATTATCATCTTGAGAACGAGGTAG
CGCGTTTGAAGAAGGGTTCCGGCTCAGGAGGATCTGGGTCAGGGTCAGGGGGTT
CCGGGTCAGGCGGGTCCGGGTCAGGCGAGGAACTGCTCTCGAAGAACTATCATC
TTGAAAATGAGGTCGCTCGATTAAAAAAGGGATCGGGCAGTGGTGGGTCCGGCTC
CGGTTCCGGAGGATCGGGATCTGGGGGCTCGGGATCCGGGGAGGAACTACTTTC
AAAGAATTACCACCTCGAAAACGAAGTAGCTCGATTAAAGAAAGGTTCAGGGTCGG
GTGGCTCAGGTTCGGGATCAGGTGGGTCAGGCTCCGGTGGTTCAGGTTCGGGAG
AAGAATTACTGAGTAAAAATTATCATCTGGAAAATGAGGTAGCGAGACTAAAAAAG

GGGAGTGGTTCTGGCGGTTCGGGATCTGGCTCTGGGGGCTCTGGGTCGGGAGGG
TCTGGGTCTGGCGAGGAATTGCTATCGAAAAATTATCATCTTGAGAACGAAGTTGC
TAGGCTCAAAAAGGGCTCAGGCTCAGGCGGGTCCGGGTCAGGGTCGGGAGGTTC
CGGATCCGGGGGATCAGGCTCAGGGTAACCCGGGGGCGCGCCATGATAACTAGA
GTCCTGCTTTAATGAGATATGCGAGACGCCTATGATCGCATGATATTTGCTTTCAAT
TCTGTTGTGCACGTTGTAAAAAACCTGAGCATGTGTAGCTCAGATCCTTACCGCCG
GTTTCGGTTCATTCTAATGAATATATCACCCGTTACTATCGTATTTTTATGAATAATA
TTCTCCGTTCAATTTACTGATTGTACCCTACTACTTATATGTACAATATTAAAATGAA
AACAATATATTGTGCTGAATAGGTTTATAGCGACATCTATGATAGAGCGCCACAATA
ACAAACAATTGCGTTTTATTATTACAAATCCAATTTTAAAAAAAGCGGCAGAACCGG
TCAAACCTAAAAGACTGATTACATAAATCTTATTCAAATTTCAAAAGTGCCCCAGGG
GCTAGTATCTACGACACACCGAGCGGCGAACTAATAACGCTCACTGAAGGGAACT
CCGGTTCCCCGCCGGCGCGCATGGGTGAGATTCCTTGAAGTTGAGTATTGGCCGT
CCGCTCTACCGAAAGTTACGGCACCATTCAACCCGGTCCAGCACGGCGGCCGG
GTAACCGACTTGCTGCCCCGAGAATTATGCAGCATTTTTTTGGTGTATGTGGGCCC
CAAATGAAGTGCAGGTCAAACCTTGACAGTGACGACAAATCGTTGGGCGGGTCCA
GGGCGAATTTTGCGACAACATGTCGAGGCTCAGCAGGACCGGCATGCAAGCTAGC
TTACTAGTGATATTCTATAGTGTCACCTAAATCTGCGGC

SunTagFWAg4-14aa-TET1cd

gRNA FWAg4
U6
NOS term
TET1cd
2xNLS
Linker
1xHA
sfGFP
scFV
UBQ10
Insulator
Omega RBC
dCAS9
3xNLS
GCN4 10x 14aa
OCS term

AAAAAAAGCACCGACTCGGTGCCACTTTTTCAAGTTGATAACGGACTAGCCTTATTT
TAACTTGCTATTTCTAGCTCTAAAACAAGCCCATACATCTTTCCGTCAATCACTACTT
CGACTCTAGCTGTATATAAACTCAGCTTCGTTTCTTATCTAAGCGATGTGGACTT
TTGAAGATTGTTTTCAACTTAAATGGGCCTATATAAGAAATACTATTGTTCTTTCCCA
TATAAATGGGCCTGCTTCTCTTCTTTCAGATTCCCAGGGGCCTTTTGAAGATTATCT
TCATATCTTAAGAATGAAGATGTTTTATTCAATCAAATTCTTGAAGGTTCGATGCCTA
ATCATTCTAATCCTGGGACAAACTATGAAACAAGATACAAAAACTCCGAATGGAAA
GTTAAAAAGAAGAAAACGAAAGCTACGGTTCAAGAAAATGTAAGCTGATAACAAA
AAAAAACTGTATGAACGAAGAAGAAGAAAAAAAGCTAAGAAGAAATGATGTATTGT

GCGGAAGGCAAGTCGAGTTTCCGTTGTTCAACGAAAGCTTAAACGTAACATAGATG
ACACCGCGCGCGATAATTTATCCTAGTTTGCGCGCTATATTTTGTTTTCTATCGCGT
ATTAAATGTATAATTGCGGGACTCTAATCATAAAAACCCATCTCATAAATAACGTCA
TGCATTACATGTTAATTATTACATGCTTAACGTAATTCAACAGAAATTATATGATAAT
CATCGCAAGACCGGCAACAGGATTCAATCTTAAGAAACTTTATTGCCAAATGTTTGA
ACGATCGGGGAAATTCGAGCTCAAACTTACGGCCATGGCGTAGGCGGCCTTTTCC
GTTGTGATCTTCGCGGTCTCCTCCTTGTTTTTGGACTAGTACCACCACCGCTACCA
CCACCTTCGGTTACCGTGAAGGTTTTGGTAGCGTCGTCGTAGGTCCATTCACCGTC
AACACCGTTGTCGTTAGCGTACTGTTTGAAAACTTTTTCCGCGGTAGCAGCGTCAA
CAGCTTCGGTGGTGGTTTCACCTTTCAGGGTTTTACCGTTCAGGATAAGCTTGTAC
TCTTCGGTCCGAGATCCTCCTCCCGTACGTCAGACCCAATGGTTATAGGGCCCCG
CAACGTGTGTGAGAGCATAAGGGGACACGGTGACAACATTGTCATGGGTTAATGT
TAATGCTTTATGAGAAGGAATTTGGTTCAATTCATTTACTTCAGAGGACTGTTCTGG
ACCTTCATTAGCTGCCTGGTCTTTTTGCTCTGAGGCCTTCATTTTCTTATTCTTAGCT
TCTTTAGCCTCAAACTTAATCTTGTTTAGTTCAAAACCATGTTGGGGCTTATTTAGGT
TTTTGTGCTGGTAAAAGACAAGGGAGAGGCGGGTTGGATGATTACGGTTGGGGTG
CTCAACAGGAGTGGTAGCGTGCAGCTCTCGCCGGGCACACTCAATCAAAACCGAG
CCGTGAGCAGGTGCGATGGCCACCCCACCAATATTTGCATCCAAAAAGATGTGCT
CACTGTCTGACCAATACTCATCAATGTGGGGCAATTTCTCCTCAGCAGGTGACAGG
GGGTCATCAGATAGGGGTTCGTCTGATGGAGGCTCATCTGCTTCAGAATGCTGCT
CATCTTCTTCCATTGGAGAAGAGGCAAGGTCTTGAGGAGAGGTGAGGAAGGAGGG
CTGGTGGTTTGGCTGATGAGGCGTTAGCGGCTCAGTCACACCAGTGGAAGGCTCA
GAATTAATGAGGGGCTCCATCACAGGAGCAGACAGGGTGGGGAGAGGAGCCACT
TCGCCAAGCTGTGAAATGCCAGGGCCATCAGCAGCTGCAGCATTGGCACCACTGA
GTCTTCCCGAAGGCATCGTACAGTGGGGAGTGCTGCTTCTTTCTGAAAACCCGCAT
GAGGCTGTTGCGTCATTCTTCAGTGGAGCTGGTGTGGCTGAAGCAGTCTTCGGGG
ACCAGGAGAAGCCTGGAGATGCCTCTTTCACTGGGTGAGGAGCGGATGGCATCA
GCGAATAAGTTTTAGTGTTGTCTGAACTTTTTAAGATAAAATGGGGTTCGGTTTCAC
TTTTTACTTCAGGTTGCACGGTCTCAGTGTTACTCCCTAAGGTTGGCAGTGACGAA
GGCTTACTGTTGTTTGTTGTTGTTGAGTTATTCTTCCGCTTGATTCGGGGAATAGGT
TTCTTTTCCACTGCCCTTATCTTATGTGCAAGAACCTCTGTCATCATCGCAGCCCTC
TTCTTTCCAGAACGGGGAACAGGCTGAGTGAAACACGTTCTTTTTTTGCGGCGGG
GTGCCAGGACCTCGATGGCCCCAGATTTGATCTTGGCTTCCATTCCTTCCTTGGAG
CCAAACTCATCTGTGTCTGAAAGCTTATAAAGAGGTAGCACATGGAGCTGCTCATC
TTGAGGAATAACACCCAAAGAGCGGTTATCTTCTCGAGTTAAGGTACAAACCACAG
TGCTTCCATTATTCATGTTGTGAATGTCCCTGTGGGGATGAGCACAGAAGTCCAGG
CAAGCAGTGACCCCAGAGAAGGGTCGACCTTCCTTGCTGCCAAGCCGACATTCTC
GGGCAACATTTTCATATTCCACCTGATTTTGGTAAGCTACTGGAGCATACTGCTTAT
AAATTGGAGCTAATCGTGTAGCCAAACTCTGTAAGTTATCTTCAAGGTTTTTTTCAT
GTAAGGGAGAGCTTGGATCAATTCTAAATCTTCTGGGGCTTGGGCTTCTACCAAAC
TTACAGCCATTAAAGTACATACTCCATGAACAGCCAAAAGAGAATGAAGCTCCACA
AGTCTCTGGATCAATTCCTTGACATGTACAGGTACGATTTTCATTGAGGGTGCATCT
TCTGTCGGTAGGGTGCCCATTGTATGACTTTAGATTCTCTGTGAGCTCTGTGTATA
GCCGGTCGGCCATTGGAAGAGGGATGCCATCCCACACCATGATGAGCACCACCAT
CACAGCAGTTGGACAGTGGTGGCCTGTACGCTGCCGGACCAAACAAAGAACTTTT
TCTTCATCACTGCTTCTTCTTAAAACCCACTTAGCAATTGGACACCCATGAGAGCTT

TTCCCTTCTTTACCGGTGTACACTACTATTTCTATCCTTATTGCGTTTCCTTTTTGAC
CATACCTATTCTCCATGATTTCCCTGACAGCAGCAACACTTGGTCCTGCCCCAAGG
TGTGTATAATATGGGCCTTTGTCTTTTTGTATAACTCGATCAAGACAGCTGCAGGTG
GGCAGCCCGGATCCGCCACCGCCCACTTTCCGCTTTTTCTTAGGAGCTTCCACCTT
GCGTTTTTTCTTGGGAGCGCCGCTGCCCAGGCTGGCATAATCGGGCACGTCATAG
GGATACGTACGACCGGTCCGACCTCCACCTTTGTAGAGCTCATCCATGCCATGTGT
AATCCCAGCAGCAGTTACAAACTCAAGAAGGACCATGTGGTCACGCTTTTCGTTGG
GATCTTTCGAAAGGACAGATTGTGTCGACAGGTAATGGTTGTCTGGTAAAAGGACA
GGGCCATCGCCAATTGGAGTATTTTGTTGATAATGGTCTGCTAGTTGAACGGAACC
ATCTTCAACGTTGTGGCGAATTTTGAAGTTAGCTTTGATTCCATTCTTTTGTTTGTCT
GCCGTGATGTATACATTGTGTGAGTTAAAGTTGTACTCGAGTTTGTGTCCAAGAAT
GTTTCCATCTTCTTTAAAATCAATACCCTTTAACTCGATACGATTAACAAGGGTATCA
CCTTCAAACTTGACTTCAGCACGCGTCTTGTAGGTCCCGTCATCTTTGAAAGATATA
GTGCGTTCCTGTACATAACCTTCGGGCATGGCACTCTTGAAAAAGTCATGCCGTTT
CATGTGATCCGGATAACGGGAAAAGCATTGAACACCATAGGTCAGAGTAGTGACA
AGTGTTGGCCACGGAACAGGTAGTTTTCCAGTAGTGCAAATAAATTTAAGGGTGAG
TTTTCCGTTTGTAGCATCACCTTCACCCTCTCCACGGACAGAAAATTTGTGCCCATT
AACATCACCATCTAATTCAACAAGAATTGGGACAACTCCAGTGAAAAGTTCTTCTCC
TTTGCTACCGCTTCCACCTCCACCTGGATCCAAGCTTCCGCCGCCTCCTGAACCAC
CACCGCCACTACCTCCTCCCCCAGAACCTCCGCCTCCACCAGCGTAATCTGGAAC
ATCGTATGGGTAGCTGCTCACGGTCACCAGGGTGCCCTGGCCCCAGTAGTCGAAC
AGGCCGGTCACGCAGTAGTACAGGGCGGTGTCGTCGCTGCGCACCTTGCTCATCT
GCAGGTACACGGTGTTCTTGCCGTTGTCCTTGCTGATGATGAAGCGGTCCTTCAG
GGCGCTGTTGTAGTCGGTGATGCCGTCGCCCCAGATCACGCCGATCCACTCCAGG
CCGCGGCCGGGGGCCTGGCGCACCCAGTTCACGCCGTAGTCGGTCAGGCTGAA
GCCGCTCACGGCGCAGCTCAGCTTCAGGCTGCCGCCGGGCTGCACCAGGCCGCC
GCCGCTCTCCAGCAGCTTCACCTCGCTGCCGCCGCCGCTGCTGCCGCCGCCGCC
GCTGCCGCCGCCGCCGCTGCCGCCGCCGCCGCGCTTCAGCTCCACCTTGGTGCC
CTGGCCGAACACCCAGTGGTTGCTGTACCACAGGGCGCAGAAGTAGGTGGCGAA
GTCCTCGGGCTGCAGGCTGCTGATGGTCAGGGTGGCCTTGTCGCCGATCAGGCT
GCCGCTGAAGCGGCTGGGCACGCCGGGGGCGCGGTTGTTGGTGCCGCCGATCA
GGCCCTTGAACAGCTTGCCGGGCTTCTCCTGCACCCAGCTGGCGTAGTTGCTGGT
GGTCACGGCGCCGGTGCTGCTGCGGCAGGTGATGGTCACGCGGTCGCCCACGCT
GGCGCTCAGGCTGCTGGGGCTCTGGGTCATCACGATGTCGGGGCCCATAAACCC
TAGGTGTTAATCAGAAAAACTCAGATTAATCGACAAATTCGATCGCACAAACTAGAA
ACTAACACCAGATCTAGATAGAAATCACAAATCGAAGAGTAATTATTCGACAAAACT
CAAATTATTTGAACAAATCGGATGATATCTATGAAACCCTAATCGAGAATTAAGATG
ATATCTAACGATCAAACCCAGAAAATCGTCTTCGATCTAAGATTAACAGAATCTAAA
CCAAAGAACATATACGAAATTGGGATCGAACGAAAACAAAATCGAAGATTTTGAGA
GAATAAGGAACACAGAAATTTACCTTGATCACGGTAGAGAGAATTGAGAGAAAGTT
TTTAAGATTTTGAGAAATTGAAATCTGAATTGTGAAGAAGAAGAGCTCTTTGGGTAT
TGTTTTATAGAAGAAGAAGAAGAAAAGACGAGGACGACTAGGTCACGAGAAAGCTA
AGGCGGTGAAGCAATAGCTAATAATAAAATGACACGTGTATTGAGCGTTGTTTACA
CGCAAAGTTGTTTTTGGCTAATTGCCTTATTTTTAGGTTGAGGAAAAGTATTTGTGC
TTTGAGTTGATAAACACGACTCGTGTGTGCCGGCTGCAACCACTTTGACGCCGTTT
ATTACTGACTCGTCGACAACCACAATTTCTAACGGTCGTCATAAGATCCAGCCGTT

GAGATTTAACGATCGTTACGATTTATATTTTTTTAGCATTATCGTTTTATTTTTTAAAT
ATACGGTGGAGCTGAAAATTGGCAATAATTGAACCGTGGGTCCCACTGCATTGAAG
CGTATTTCGTATTTTCTAGAATTCTTCGTGCTTTATTTCTTTTCCTTTTTGTTTTTTTTT
GCCATTTATCTAATGCAAGTGGGCTTATAAAATCAGTGAATTTCTTGGAAAAGTAAC
TTCTTTATCGTATAACATATTGTGAAATTATCCATTTCTTTTAATTTTTTAGTGTTATT
GGATATTTTTGTATGATTATTGATTTGCATAGGATAATGACTTTTGTATCAAGTTGGT
GAACAAGTCTCGTTAAAAAAGGCAAGTGGTTTGGTGACTCGATTTATTCTTGTTATT
TAATTCATATATCAATGGATCTTATTTGGGGCCTGGTCCATATTTAACACTCGTGTT
CAGTCCAATGACCAATAATATTTTTTCATTAATAACAATGTAACAAGAATGATACACA
AAACATTCTTTGAATAAGTTCGCTATGAAGAAGGGAACTTATCCGGTCCTAGATCAT
CAGTTCATACAAACCTCCATAGAGTTCAACATCTTAAACAAGAATATCCTGATCTGA
AGAATGTGGAGGCTTTAGTCCCTTGGATACTTGGGAGGCTGTGGAAGAACAGAAA
CGAGCTGGTGCTTAAAGGGAGGGAATTTGGAACCAATGAGGTATTAGTAAGGACA
CAAGAAGATGCAGATGAGTGGATTAGAAGGAAAGAGGCTCAGAATGTAAGGAAAG
CACCAACCACAACACCGTCGGGACAGACGAGAAACATGGGAAGCTCCACCACAAT
CTTGGGTTAAGTGCAACTTTGATGGGGCATGGCCAACAGAAGGATTAAAATGTGG
CTTAGGGTGGGTGCTTCGCGATCATACAGGGAAGGTGTTATGGTTAGGTGCAAGA
GCTGTGGTAAAAGTAAGAAGCGTGCTGGAAGTAGAAGTGGAGGCTCTTAGATGGG
CTGTGCTGTCATTATCCCGATTCAATTATAGGAAGATCATTTTTGAGGTGGATTCTC
AGCAACTTGTGTCTTTGGTTACATGAAAGTTATGCTTGTCAAGTCTCAATCCAATTA
TCCAAGACATAAAGTATCTACTTAGCAAGTTTGAGGATTTTATGCTTGTGCATACAA
GCCGAGAAGGAAATGGAGTGGCAGATAGAATAGCTAAGGAATCTCTTTCTTTTGAG
AATTATGATCTAAAGTTGTATTCTATTGTACCAATTTGGGTTAAAAGCTCTGTTGAGC
TAGACTCATATGCATCCTAGGAAACAAGTTGTAATGAGTTGCTGGCCTCTCTTGGG
ATTGTTCATCAAAGTAGCTGTCCCTACACACCACAACAGAATGGTGTTGTGGAGAG
AAAGCACAGACACATCCTTGAGATGGCAAGGGCACTTAAGTTTCAGAGTGGTGTAC
CTACCAGGTTTTGGGGAGACTGTGTCAAGACTGTTGTATACCTGATCAATAGACTC
CCTACTCCAATCTTACAAGGCAAATGTCCATATGAACTACTCTATCAGAAACATGCC
AAGCTTGATCACTTGAGAGTGTTTGGGTGTCTTGCTTTTGCAGCAACACTGCCCAA
AGGTGACAAACTTGCACCTAGAGCCAAGCGAACCATCTTCATTGGTTATTCTGAGA
CTCAAAAGGGTTACAGGTTGTATGACTTGGATAATAAGGTGATCATAGTGAGCAGG
GATGTAGTTTTCAGAGAGTTTCAGTTCCCTTTCAAAGAGTCCATCTCCCATGAACCT
GATATGTTCACTCGAGCAGGTTCACTTAGCTCTGAAGATACAGCTTTGCAGCTAGT
GTATAATGATATTTTTCCTTCCAGCTTTCATACTGATGATGATGATACTCCAGTTCTT
ATTCCTGACATTGTAGAGGCCAATGAGGATATCACTTTGGGGACTCATTCTACCCA
AAATCATATCACTTCAGCTGATGCTGCACCTTCAGGCCACCTGCATGCTCCTGCAG
AGTCAGTTGAGTCAGCTGAGCCTGACCTAGCAAATGCTGAATTTGAGCACCATACT
GCTGTAGCTGATCCCTCTCTAGTTCCAGCAAACCTGCACACTAGACCTAAACGCAA
TGCAGGTCCTCCCATCTGGCTCAAGGACTTTGTGACACTGAACAAAGGCTCTAGG
GATGTTCCATACCCTATAGCCAACTATGTTTCCTATGATCACTTGCCTGTTCACTAT
CAAGCTTATTTGAGTGCTTTCTCCACTTATACTGAACCTAAATCCTTCAAAGAAGCA
GCTCAAGATGAGAAATGGATGGAGGCCATGTCCCTTGAGATACAGGCTCTTGAGG
ATAATAACACCTGGGAGATTGTCCCTTTACCCCCTGGTAAACAGCCTATAGGGTCC
AAATGGGTGTACAAAATTAAATACAAAGCTAATGGTGAAGTTGACAGGTTTAAGGC
AAGGCTAGTGGCCAAGGGATACACTCAGCAAGAAGGCCTTGACTACCATGAAACT
TTTTCTCCAGTGGCCAAAATGGTCACTGTAAAAGCTGTCATATCTGTTGCTGCTTCC

AAGGGCTGGTTCCTTTTCCAGATGGATGTCAACAATGCTTTTCTACAAGGTGACCT
CATGGAAGAAGTTTATATGTCTCTGCCTCAGGGTTTTCACAGCCAAGGGGAGACCA
AAGTGTGCAGGCTGTTGAATCCCTCTATGGTTTAAAGCAAGCATCAAGGCAGTGGA
ACATCAAGCTTACCACTGTCCTTATGCAGGCTGGTTTTATGCAAAGTGCTTATGATC
ACTCCTTGTTTACCAAAAGAGAGGGGACTGACCTTGTCATAATCCTGATTTATGTG
GATGATCTGTTAATAACAGGCAGCAGCAATGTTTTGATTTCAGAAGCAAAGGCAAC
CCTGCATCAGCATTTCAAGATGAAAGATTTGGGAGAACTAAAATACTTCTTAGGCAT
TGAGGTGCTGAGATCAGAAAAGGGAATCCTACTGAACCAGAGGAAGTATGCACTG
GAACTGATATCAGGTGTTGGTCTGGGAGGCTGCAGACCAGTGTCAACCCCAATGG
AACAGAACCAAAGGCTGACTACTGTAGAATATGACAAGCACTTAGGAAAGACTGAT
GATGCAGAGTTAGAGGATGTGGGTTCCTATCAGAGACTGGTGGGAAAGCTTCTCT
ACTTGACAATCACAAGGCCAGATATCAGCTTTGCAGTGCAGGTGCTGAGTCAATTC
ATGCAGCAACCCAAACAGTCACACCTGGAAGCAGCATTGAGGGTTGTGAAGTACA
TAAAAGGTTCTCCAGGTGTTAGGAAGCGGCCGCATGCATATGAGTCTAGCTCAACA
GAGCTTTTAACCCAAATTGGTACAATAGAATACAACTTTAGATCATAATTCTCAAAA
GAAAGAGATTCCTTAGCTATTCTATCTGCCACTCCATTTCCTTCTCGGCTTGTATGC
ACAAGCATAAAATCCTCAAACTTGCTAAGTAGATACTTTATGTCTTGGATAATTGGA
TTGAGACTTGACAAGCATAACTTTCATGTAACCAAAGACACAAGTTGCTGAGAATC
CACCTCAAAAATGATCTTCCTATAATTGAATCGGGATAATGACAGCACAGCCCATCT
AAGAGCCTCCACTTCTACTTCCAGCACGCTTCTTACTTTTACCACAGCTCTTGCACC
TAACCATAACACCTTCCCTGTATGATCGCGAAGCACCCACCCTAAGCCACATTTTA
ATCCTTCTGTTGGCCATGCCCCATCAAAGTTGCACTTAACCCAAGATTGTGGTGGA
GCTTCCCATGTTTCTCGTCTGTCCCGACGGTGTTGTGGTTGGTGCTTTCCTTACAT
TCTGAGCCTCTTTCCTTCTAATCCACTCATCTGCATCTTCTTGTGTCCTTACTAATAC
CTCATTGGTTCCAAATTCCCTCCCTTTAAGCACCAGCTCGTTTCTGTTCTTCCACAG
CCTCCCAAGTATCCAAGGGACTAAAGCCTCCACATTCTTCAGATCAGGATATTCTT
GTTTAAGATGTTGAACTCTATGGAGGTTTGTATGAACTGATGATCTAGGACCGGAT
AAGTTCCCTTCTTCATAGCGAACTTATTCAAAGAATGTTTTGTGTATCATTCTTGTTA
CATTGTTATTAATGAAAAAATATTATTGGTCATTGGACTGAACACGAGTGTTAAATAT
GGACCAGGCCCCAAATAAGATCCATTGATATATGAATTAAATAACAAGAATAAATCG
AGTCACCAAACCACTTGCCTTTTTTAACGAGACTTGTTCACCAACTTGATACAAAAG
TCATTATCCTATGCAAATCAATAATCATACAAAAATATCCAATAACACTAAAAAATTA
AAAGAAATGGATAATTTCACAATATGTTATACGATAAAGAAGTTACTTTTCCAAGAAA
TTCACTGATTTTATAAGCCCACTTGCATTAGATAAATGGCAAAAAAAAACAAAAAGG
AAAAGAAATAAAGCACGAAGAATTCTAGAAAATACGAAATACGCTTCAATGCAGTG
GGACCCACGGTTCAATTATTGCCAATTTTCAGCTCCACCGTATATTTAAAAAATAAA
ACGATAATGCTAAAAAAATATAAATCGTAACGATCGTTAAATCTCAACGGCTGGATC
TTATGACGACCGTTAGAAATTGTGGTTGTCGACGAGTCAGTAATAAACGGCGTCAA
AGTGGTTGCAGCCGGCACACACGAGTCGTGTTTATCAACTCAAAGCACAAATACTT
TTCCTCAACCTAAAAATAAGGCAATTAGCCAAAAACAACTTTGCGTGTAAACAACGC
TCAATACACGTGTCATTTTATTATTAGCTATTGCTTCACCGCCTTAGCTTTCTCGTGA
CCTAGTCGTCCTCGTCTTTTCTTCTTCTTCTTCTATAAAACAATACCCAAAGAGCTCT
TCTTCTTCACAATTCAGATTTCAATTTCTCAAAATCTTAAAAACTTTCTCTCAATTCTC
TCTACCGTGATCAAGGTAAATTTCTGTGTTCCTTATTCTCTCAAAATCTTCGATTTTG
TTTTCGTTCGATCCCAATTTCGTATATGTTCTTTGGTTTAGATTCTGTTAATCTTAGA
TCGAAGACGATTTTCTGGGTTTGATCGTTAGATATCATCTTAATTCTCGATTAGGGT

TTCATAGATATCATCCGATTTGTTCAAATAATTTGAGTTTTGTCGAATAATTACTCTT
CGATTTGTGATTTCTATCTAGATCTGGTGTTAGTTTCTAGTTTGTGCGATCGAATTT
GTCGATTAATCTGAGTTTTTCTGATTAACAGGGATCATCAACAAGTTTGTACAAAAA
AGCAGGCTCTTTAAAGTATTTTTACAACAATTACCAACAACAACAAACAACAAACAA
CATTACAATTACTATTTACAATTACAAAAAAAGTTAACATGGACAAGAAGTACAGCA
TCGGCCTGGCCATCGGCACCAACTCTGTGGGCTGGGCCGTGATCACCGACGAGT
ACAAGGTGCCCAGCAAGAAATTCAAGGTGCTGGGCAACACCGACCGGCACAGCAT
CAAGAAGAACCTGATCGGCGCCCTGCTGTTCGACAGCGGAGAAACAGCCGAGGC
CACCCGGCTGAAGAGAACCGCCAGAAGAAGATACACCAGACGGAAGAACCGGAT
CTGCTATCTGCAAGAGATCTTCAGCAACGAGATGGCCAAGGTGGACGACAGCTTC
TTCCACAGACTGGAAGAGTCCTTCCTGGTGGAAGAGGATAAGAAGCACGAGCGGC
ACCCCATCTTCGGCAACATCGTGGACGAGGTGGCCTACCACGAGAAGTACCCCAC
CATCTACCACCTGAGAAAGAAACTGGTGGACAGCACCGACAAGGCCGACCTGCGG
CTGATCTATCTGGCCCTGGCCCACATGATCAAGTTCCGGGGCCACTTCCTGATCG
AGGGCGACCTGAACCCCGACAACAGCGACGTGGACAAGCTGTTCATCCAGCTGGT
GCAGACCTACAACCAGCTGTTCGAGGAAAACCCCATCAACGCCAGCGGCGTGGAC
GCCAAGGCCATCCTGTCTGCCAGACTGAGCAAGAGCAGACGGCTGGAAAATCTGA
TCGCCCAGCTGCCCGGCGAGAAGAAGAATGGCCTGTTCGGCAACCTGATTGCCCT
GAGCCTGGGCCTGACCCCCAACTTCAAGAGCAACTTCGACCTGGCCGAGGATGC
CAAACTGCAGCTGAGCAAGGACACCTACGACGACGACCTGGACAACCTGCTGGCC
CAGATCGGCGACCAGTACGCCGACCTGTTTCTGGCCGCCAAGAACCTGTCCGACG
CCATCCTGCTGAGCGACATCCTGAGAGTGAACACCGAGATCACCAAGGCCCCCCT
GAGCGCCTCTATGATCAAGAGATACGACGAGCACCACCAGGACCTGACCCTGCTG
AAAGCTCTCGTGCGGCAGCAGCTGCCTGAGAAGTACAAAGAGATTTTCTTCGACC
AGAGCAAGAACGGCTACGCCGGCTACATCGATGGCGGAGCCAGCCAGGAAGAGT
TCTACAAGTTCATCAAGCCCATCCTGGAAAAGATGGACGGCACCGAGGAACTGCT
CGTGAAGCTGAACAGAGAGGACCTGCTGCGGAAGCAGCGGACCTTCGACAACGG
CAGCATCCCCCACCAGATCCACCTGGGAGAGCTGCACGCCATTCTGCGGCGGCA
GGAAGATTTTTACCCATTCCTGAAGGACAACCGGGAAAAGATCGAGAAGATCCTGA
CCTTCCGCATCCCCTACTACGTGGGCCCTCTGGCCAGGGGAAACAGCAGATTCGC
CTGGATGACCAGAAAGAGCGAGGAAACCATCACCCCCTGGAACTTCGAGGAAGTG
GTGGACAAGGGCGCCAGCGCCCAGAGCTTCATCGAGCGGATGACCAACTTCGAT
AAGAACCTGCCCAACGAGAAGGTGCTGCCCAAGCACAGCCTGCTGTACGAGTACT
TCACCGTGTACAACGAGCTGACCAAAGTGAAATACGTGACCGAGGGAATGAGAAA
GCCCGCCTTCCTGAGCGGCGAGCAGAAAAAAGCCATCGTGGACCTGCTGTTCAAG
ACCAACCGGAAAGTGACCGTGAAGCAGCTGAAAGAGGACTACTTCAAGAAAATCG
AGTGCTTCGACTCCGTGGAAATCTCCGGCGTGGAAGATCGGTTCAACGCCTCCCT
GGGCACATACCACGATCTGCTGAAAATTATCAAGGACAAGGACTTCCTGGACAATG
AGGAAAACGAGGACATTCTGGAAGATATCGTGCTGACCCTGACACTGTTTGAGGA
CAGAGAGATGATCGAGGAACGGCTGAAAACCTATGCCCACCTGTTCGACGACAAA
GTGATGAAGCAGCTGAAGCGGCGGAGATACACCGGCTGGGGCAGGCTGAGCCG
GAAGCTGATCAACGGCATCCGGGACAAGCAGTCCGGCAAGACAATCCTGGATTTC
CTGAAGTCCGACGGCTTCGCCAACAGAAACTTCATGCAGCTGATCCACGACGACA
GCCTGACCTTTAAAGAGGACATCCAGAAAGCCCAGGTGTCCGGCCAGGGCGATAG
CCTGCACGAGCACATTGCCAATCTGGCCGGCAGCCCCGCCATTAAGAAGGGCATC
CTGCAGACAGTGAAGGTGGTGGACGAGCTCGTGAAAGTGATGGGCCGGCACAAG

CCCGAGAACATCGTGATCGAAATGGCCAGAGAGAACCAGACCACCCAGAAGGGA
CAGAAGAACAGCCGCGAGAGAATGAAGCGGATCGAAGAGGGCATCAAAGAGCTG
GGCAGCCAGATCCTGAAAGAACACCCCGTGGAAAACACCCAGCTGCAGAACGAGA
AGCTGTACCTGTACTACCTGCAGAATGGGCGGGATATGTACGTGGACCAGGAACT
GGACATCAACCGGCTGTCCGACTACGATGTGGACGCTATCGTGCCTCAGAGCTTT
CTGAAGGACGACTCCATCGATAACAAAGTGCTGACTCGGAGCGACAAGAACCGGG
GCAAGAGCGACAACGTGCCCTCCGAAGAGGTCGTGAAGAAGATGAAGAACTACTG
GCGCCAGCTGCTGAATGCCAAGCTGATTACCCAGAGGAAGTTCGACAATCTGACC
AAGGCCGAGAGAGGCGGCCTGAGCGAACTGGATAAGGCCGGCTTCATCAAGAGA
CAGCTGGTGGAAACCCGGCAGATCACAAAGCACGTGGCACAGATCCTGGACTCCC
GGATGAACACTAAGTACGACGAGAACGACAAACTGATCCGGGAAGTGAAAGTGAT
CACCCTGAAGTCCAAGCTGGTGTCCGATTTCCGGAAGGATTTCCAGTTTTACAAAG
TGCGCGAGATCAACAACTACCACCACGCCCACGACGCCTACCTGAACGCCGTCGT
GGGAACCGCCCTGATCAAAAAGTACCCTAAGCTGGAAAGCGAGTTCGTGTACGGC
GACTACAAGGTGTACGACGTGCGGAAGATGATCGCCAAGAGCGAGCAGGAAATC
GGCAAGGCTACCGCCAAGTACTTCTTCTACAGCAACATCATGAACTTTTTCAAGAC
CGAGATTACCCTGGCCAACGGCGAGATCCGGAAGCGGCCTCTGATCGAGACAAA
CGGCGAAACAGGCGAGATCGTGTGGGATAAGGGCCGGGACTTTGCCACCGTGCG
GAAAGTGCTGTCTATGCCCCAAGTGAATATCGTGAAAAAGACCGAGGTGCAGACA
GGCGGCTTCAGCAAAGAGTCTATCCTGCCCAAGAGGAACAGCGACAAGCTGATCG
CCAGAAAGAAGGACTGGGACCCTAAGAAGTACGGCGGCTTCGACAGCCCCACCG
TGGCCTATTCTGTGCTGGTGGTGGCCAAAGTGGAAAAGGGCAAGTCCAAGAAACT
GAAGAGTGTGAAAGAGCTGCTGGGGATCACCATCATGGAAAGAAGCAGCTTCGAG
AAGAATCCCATCGACTTTCTGGAAGCCAAGGGCTACAAAGAAGTGAAAAAGGACCT
GATCATCAAGCTGCCTAAGTACTCCCTGTTCGAGCTGGAAAACGGCCGGAAGAGA
ATGCTGGCCTCTGCCGGCGAACTGCAGAAGGGAAACGAACTGGCCCTGCCCTCC
AAATATGTGAACTTCCTGTACCTGGCCAGCCACTATGAGAAGCTGAAGGGCTCCC
CCGAGGATAATGAGCAGAAACAGCTGTTTGTGGAACAGCACAAACACTACCTGGA
CGAGATCATCGAGCAGATCAGCGAGTTCTCCAAGAGAGTGATCCTGGCCGACGCT
AATCTGGACAAGGTGCTGAGCGCCTACAACAAGCACAGAGACAAGCCTATCAGAG
AGCAGGCCGAGAATATCATCCACCTGTTTACCCTGACCAATCTGGGAGCCCCTGC
CGCCTTCAAGTACTTTGACACCACCATCGACCGGAAGAGGTACACCAGCACCAAA
GAGGTGCTGGACGCCACCCTGATCCACCAGAGCATCACCGGCCTGTACGAGACA
CGGATCGACCTGTCTCAGCTGGGAGGCGACGCCTATCCCTATGACGTGCCCGATT
ATGCCAGCCTGGGCAGCGGCTCCCCCAAGAAAAAACGCAAGGTGGAAGATCCTAA
GAAAAAGCGGAAAGTGGAAGATGCTCCAAAGAAGAAGAGAAAGGTCGACGGCATT
GGTAGTGGGAGCAACGGCAGCAGCGGATCCAACGGTCCGACTGACGCCGCGGAA
GAAGAACTTTTGAGCAAGAATTATCATCTTGAGAACGAAGTGGCTCGTCTTAAGAA
AGGTTCTGGCAGTGGAGGTTCTGGCTCCGGAGGTTCCGGCGGGGAAGAACTGCT
TTCAAAGAATTACCACCTGGAAAATGAGGTAGCTAGACTGAAAAAGGGGAGCGGA
AGTGGGGGCTCCGGGTCGGGCGGCTCTGGGGGAGAGGAGTTGCTGAGCAAAAAT
TATCATTTGGAGAACGAAGTAGCACGACTAAAGAAAGGGTCCGGATCGGGTGGTT
CAGGATCTGGAGGTTCGGGTGGGGAGGAGTTACTCTCGAAAAATTATCATCTCGA
AAACGAAGTGGCTCGGCTAAAAAAGGGCAGTGGTTCTGGAGGATCTGGGTCGGG
GGGATCCGGGGGTGAAGAGCTATTATCTAAAAACTACCACCTCGAAAATGAGGTG
GCACGCTTAAAAAAGGGAAGTGGCAGTGGTGGGTCGGGATCTGGCGGGTCGGGT

GGAGAAGAGCTACTATCCAAGAATTATCATCTTGAGAACGAGGTAGCGCGTTTGAA
GAAGGGTTCCGGCTCAGGAGGATCTGGGTCAGGGGGCTCAGGAGGGGAGGAACT
GCTCTCGAAGAACTATCATCTTGAAAATGAGGTCGCTCGATTAAAAAAGGGATCGG
GCAGTGGTGGGTCCGGCTCCGGTGGTTCGGGGGGCGAGGAACTACTTTCAAAGA
ATTACCACCTCGAAAACGAAGTAGCTCGATTAAAGAAAGGTTCAGGGTCGGGTGG
CTCAGGTTCGGGAGGATCCGGCGGTGAAGAATTACTGAGTAAAAATTATCATCTGG
AAAATGAGGTAGCGAGACTAAAAAAGGGGAGTGGTTCTGGCGGTTCGGGATCTGG
CGGGTCGGGAGGGGAGGAATTGCTATCGAAAAATTATCATCTTGAGAACGAAGTT
GCTAGGCTCAAAAAGGGCTCAGGCTCAGGCGGGTCCGGGTCAGGGGGCTCAGGT
GGCTAACCCGGGGGCGCGCCATGATAACTAGAGTCCTGCTTTAATGAGATATGCG
AGACGCCTATGATCGCATGATATTTGCTTTCAATTCTGTTGTGCACGTTGTAAAAAA
CCTGAGCATGTGTAGCTCAGATCCTTACCGCCGGTTTCGGTTCATTCTAATGAATA
TATCACCCGTTACTATCGTATTTTTATGAATAATATTCTCCGTTCAATTTACTGATTG
TACCCTACTACTTATATGTACAATATTAAAATGAAAACAATATATTGTGCTGAATAGG
TTTATAGCGACATCTATGATAGAGCGCCACAATAACAAACAATTGCGTTTTATTATT
ACAAATCCAATTTTAAAAAAAGCGGCAGAACCGGTCAAACCTAAAAGACTGATTAC
ATAAATCTTATTCAAATTTCAAAAGTGCCCCAGGGGCTAGTATCTACGACACACCG
AGCGGCGAACTAATAACGCTCACTGAAGGGAACTCCGGTTCCCCGCCGGCGCGC
ATGGGTGAGATTCCTTGAAGTTGAGTATTGGCCGTCCGCTCTACCGAAAGTTACGG
GCACCATTCAACCCGGTCCAGCACGGCGGCCGGGTAACCGACTTGCTGCCCCGA
GAATTATGCAGCATTTTTTTGGTGTATGTGGGCCCCAAATGAAGTGCAGGTCAAAC
CTTGACAGTGACGACAAATCGTTGGGCGGGTCCAGGGCGAATTTTGCGACAACAT
GTCGAGGCTCAGCAGGACCGGCATGCAAGCTAGCTTACTAGTGATATTCTATAGT
GTCACCTAAATCTGCGGC

SunTagCACTA1g2-22aa-TET1cd

gRNA CACTA1g2
U6
NOS term
TET1cd
2xNLS
Linker
1xHA
sfGFP
scFV
UBQ10
Insulator
Omega RBC
dCAS9
3xNLS
GCN4 10x 22aa
OCS term

AAAAAAAGCACCGACTCGGTGCCACTTTTTCAAGTTGATAACGGACTAGCCTTATTT
TAACTTGCTATTTCTAGCTCTAAAACCTACTGCTATCAATGAGGACCAATCACTACT
TCGACTCTAGCTGTATATAAACTCAGCTTCGTTTTCTTATCTAAGCGATGTGGACT
TTTGAAGATTGTTTTCAACTTAAATGGGCCTATATAAGAAATACTATTGTTCTTTCCC
ATATAAATGGGCCTGCTTCTCTTCTTTCAGATTCCCAGGGGCCTTTTGAAGATTATC
TTCATATCTTAAGAATGAAGATGTTTTATTCAATCAAATTCTTGAAGGTTCGATGCCT
AATCATTCTAATCCTGGGACAAACTATGAAACAAGATACAAAAACTCCGAATGGAAA
GTTAAAAAGAAGAAAACGAAAGCTACGGTTCAAGAAAATGTAAGCTGATAAACAAA
AAAAAACTGTATGAACGAAGAAGAAGAAAAAAAGCTAAGAAGAAATGATGTATTGT
GCGGAAGGCAAGTCGAGTTTCCGTTGTTCAACGAAAGCTTAAACGTAACATAGATG
ACACCGCGCGCGATAATTTATCCTAGTTTGCGCGCTATATTTTGTTTTCTATCGCGT
ATTAAATGTATAATTGCGGGACTCTAATCATAAAAACCCATCTCATAAATAACGTCA
TGCATTACATGTTAATTATTACATGCTTAACGTAATTCAACAGAAATTATATGATAAT
CATCGCAAGACCGGCAACAGGATTCAATCTTAAGAAACTTTATTGCCAAATGTTTGA
ACGATCGGGGAAATTCGAGCTCAAACTTACGGCCATGGCGTAGGCGGCCTTTTCC
GTTGTGATCTTCGCGGTCTCCTCCTTGTTTTTGGACTAGTACCACCACCGCTACCA
CCACCTTCGGTTACCGTGAAGGTTTTGGTAGCGTCGTCGTAGGTCCATTCACCGTC
AACACCGTTGTCGTTAGCGTACTGTTTGAAAACTTTTTCCGCGGTAGCAGCGTCAA
CAGCTTCGGTGGTGGTTTCACCTTTCAGGGTTTTACCGTTCAGGATAAGCTTGTAC
TCTTCGGTCCGAGATCCTCCTCCCGTACGTCAGACCCAATGGTTATAGGGCCCCG
CAACGTGTGTGAGAGCATAAGGGGACACGGTGACAACATTGTCATGGGTTAATGT
TAATGCTTTATGAGAAGGAATTTGGTTCAATTCATTTACTTCAGAGGACTGTTCTGG
ACCTTCATTAGCTGCCTGGTCTTTTTGCTCTGAGGCCTTCATTTTCTTATTCTTAGCT
TCTTTAGCCTCAAACTTAATCTTGTTTAGTTCAAAACCATGTTGGGGCTTATTTAGGT
TTTTGTGCTGGTAAAAGACAAGGGAGAGGCGGGTTGGATGATTACGGTTGGGGTG
CTCAACAGGAGTGGTAGCGTGCAGCTCTCGCCGGGCACACTCAATCAAAACCGAG
CCGTGAGCAGGTGCGATGGCCACCCCACCAATATTTGCATCCAAAAAGATGTGCT
CACTGTCTGACCAATACTCATCAATGTGGGGCAATTTCTCCTCAGCAGGTGACAGG
GGGTCATCAGATAGGGGTTCGTCTGATGGAGGCTCATCTGCTTCAGAATGCTGCT
CATCTTCTTCCATTGGAGAAGAGGCAAGGTCTTGAGGAGAGGTGAGGAAGGAGGG
CTGGTGGTTTGGCTGATGAGGCGTTAGCGGCTCAGTCACACCAGTGGAAGGCTCA
GAATTAATGAGGGGCTCCATCACAGGAGCAGACAGGGTGGGGAGAGGAGCCACT
TCGCCAAGCTGTGAAATGCCAGGGCCATCAGCAGCTGCAGCATTGGCACCACTGA
GTCTTCCCGAAGGCATCGTACAGTGGGGAGTGCTGCTTCTTTCTGAAAACCCGCAT
GAGGCTGTTGCGTCATTCTTCAGTGGAGCTGGTGTGGCTGAAGCAGTCTTCGGGG
ACCAGGAGAAGCCTGGAGATGCCTCTTTCACTGGGTGAGGAGCGGATGGCATCA
GCGAATAAGTTTTAGTGTTGTCTGAACTTTTTAAGATAAAATGGGGTTCGGTTTCAC
TTTTTACTTCAGGTTGCACGGTCTCAGTGTTACTCCCTAAGGTTGGCAGTGACGAA
GGCTTACTGTTGTTTGTTGTTGTTGAGTTATTCTTCCGCTTGATTCGGGGAATAGGT
TTCTTTTCCACTGCCCTTATCTTATGTGCAAGAACCTCTGTCATCATCGCAGCCCTC
TTCTTTCCAGAACGGGGAACAGGCTGAGTGAAACACGTTCTTTTTTTGCGGCGGG
GTGCCAGGACCTCGATGGCCCCAGATTTGATCTTGGCTTCCATTCCTTCCTTGGAG
CCAAACTCATCTGTGTCTGAAAGCTTATAAAGAGGTAGCACATGGAGCTGCTCATC
TTGAGGAATAACACCCAAAGAGCGGTTATCTTCTCGAGTTAAGGTACAAACCACAG
TGCTTCCATTATTCATGTTGTGAATGTCCCTGTGGGGATGAGCACAGAAGTCCAGG
CAAGCAGTGACCCCAGAGAAGGGTCGACCTTCCTTGCTGCCAAGCCGACATTCTC

GGGCAACATTTTCATATTCCACCTGATTTTGGTAAGCTACTGGAGCATACTGCTTAT
AAATTGGAGCTAATCGTGTAGCCAAACTCTGTAAGTTATCTTCAAGGTTTTTTTCAT
GTAAGGGAGAGCTTGGATCAATTCTAAATCTTCTGGGGCTTGGGCTTCTACCAAAC
TTACAGCCATTAAAGTACATACTCCATGAACAGCCAAAAGAGAATGAAGCTCCACA
AGTCTCTGGATCAATTCCTTGACATGTACAGGTACGATTTTCATTGAGGGTGCATCT
TCTGTCGGTAGGGTGCCCATTGTATGACTTTAGATTCTCTGTGAGCTCTGTGTATA
GCCGGTCGGCCATTGGAAGAGGGATGCCATCCCACACCATGATGAGCACCACCAT
CACAGCAGTTGGACAGTGGTGGCCTGTACGCTGCCGGACCAAACAAAGAACTTTT
TCTTCATCACTGCTTCTTCTTAAAACCCACTTAGCAATTGGACACCCATGAGAGCTT
TTCCCTTCTTTACCGGTGTACACTACTATTTCTATCCTTATTGCGTTTCCTTTTTGAC
CATACCTATTCTCCATGATTTCCCTGACAGCAGCAACACTTGGTCCTGCCCCAAGG
TGTGTATAATATGGGCCTTTGTCTTTTTGTATAACTCGATCAAGACAGCTGCAGGTG
GGCAGCCCGGATCCGCCACCGCCCACTTTCCGCTTTTTCTTAGGAGCTTCCACCTT
GCGTTTTTTCTTGGGAGCGCCGCTGCCCAGGCTGGCATAATCGGGCACGTCATAG
GGATACGTACGACCGGTCCGACCTCCACCTTTGTAGAGCTCATCCATGCCATGTGT
AATCCCAGCAGCAGTTACAAACTCAAGAAGGACCATGTGGTCACGCTTTTCGTTGG
GATCTTTCGAAAGGACAGATTGTGTCGACAGGTAATGGTTGTCTGGTAAAAGGACA
GGGCCATCGCCAATTGGAGTATTTTGTTGATAATGGTCTGCTAGTTGAACGGAACC
ATCTTCAACGTTGTGGCGAATTTTGAAGTTAGCTTTGATTCCATTCTTTTGTTTGTCT
GCCGTGATGTATACATTGTGTGAGTTAAAGTTGTACTCGAGTTTGTGTCCAAGAAT
GTTTCCATCTTCTTTAAAATCAATACCCTTTAACTCGATACGATTAACAAGGGTATCA
CCTTCAAACTTGACTTCAGCACGCGTCTTGTAGGTCCCGTCATCTTTGAAAGATATA
GTGCGTTCCTGTACATAACCTTCGGGCATGGCACTCTTGAAAAAGTCATGCCGTTT
CATGTGATCCGGATAACGGGAAAAGCATTGAACACCATAGGTCAGAGTAGTGACA
AGTGTTGGCCACGGAACAGGTAGTTTTCCAGTAGTGCAAATAAATTTAAGGGTGAG
TTTTCCGTTTGTAGCATCACCTTCACCCTCTCCACGGACAGAAAATTTGTGCCCATT
AACATCACCATCTAATTCAACAAGAATTGGGACAACTCCAGTGAAAAGTTCTTCTCC
TTTGCTACCGCTTCCACCTCCACCTGGATCCAAGCTTCCGCCGCCTCCTGAACCAC
CACCGCCACTACCTCCTCCCCCAGAACCTCCGCCTCCACCAGCGTAATCTGGAAC
ATCGTATGGGTAGCTGCTCACGGTCACCAGGGTGCCCTGGCCCCAGTAGTCGAAC
AGGCCGGTCACGCAGTAGTACAGGGCGGTGTCGTCGCTGCGCACCTTGCTCATCT
GCAGGTACACGGTGTTCTTGCCGTTGTCCTTGCTGATGATGAAGCGGTCCTTCAG
GGCGCTGTTGTAGTCGGTGATGCCGTCGCCCCAGATCACGCCGATCCACTCCAGG
CCGCGGCCGGGGGCCTGGCGCACCCAGTTCACGCCGTAGTCGGTCAGGCTGAA
GCCGCTCACGGCGCAGCTCAGCTTCAGGCTGCCGCCGGGCTGCACCAGGCCGCC
GCCGCTCTCCAGCAGCTTCACCTCGCTGCCGCCGCCGCTGCTGCCGCCGCCGCC
GCTGCCGCCGCCGCCGCTGCCGCCGCCGCCGCGCTTCAGCTCCACCTTGGTGCC
CTGGCCGAACACCCAGTGGTTGCTGTACCACAGGGCGCAGAAGTAGGTGGCGAA
GTCCTCGGGCTGCAGGCTGCTGATGGTCAGGGTGGCCTTGTCGCCGATCAGGCT
GCCGCTGAAGCGGCTGGGCACGCCGGGGGCGCGGTTGTTGGTGCCGCCGATCA
GGCCCTTGAACAGCTTGCCGGGCTTCTCCTGCACCCAGCTGGCGTAGTTGCTGGT
GGTCACGGCGCCGGTGCTGCTGCGGCAGGTGATGGTCACGCGGTCGCCCACGCT
GGCGCTCAGGCTGCTGGGGCTCTGGGTCATCACGATGTCGGGGCCCATAAACCC
TAGGTGTTAATCAGAAAAACTCAGATTAATCGACAAATTCGATCGCACAAACTAGAA
ACTAACACCAGATCTAGATAGAAATCACAAATCGAAGAGTAATTATTCGACAAAACT
CAAATTATTTGAACAAATCGGATGATATCTATGAAACCCTAATCGAGAATTAAGATG

ATATCTAACGATCAAACCCAGAAAATCGTCTTCGATCTAAGATTAACAGAATCTAAA
CCAAAGAACATATACGAAATTGGGATCGAACGAAAACAAAATCGAAGATTTTGAGA
GAATAAGGAACACAGAAATTTACCTTGATCACGGTAGAGAGAATTGAGAGAAAGTT
TTTAAGATTTTGAGAAATTGAAATCTGAATTGTGAAGAAGAAGAGCTCTTTGGGTAT
TGTTTTATAGAAGAAGAAGAAGAAAAGACGAGGACGACTAGGTCACGAGAAAGCTA
AGGCGGTGAAGCAATAGCTAATAATAAAATGACACGTGTATTGAGCGTTGTTTACA
CGCAAAGTTGTTTTTGGCTAATTGCCTTATTTTTAGGTTGAGGAAAAGTATTTGTGC
TTTGAGTTGATAAACACGACTCGTGTGTGCCGGCTGCAACCACTTTGACGCCGTTT
ATTACTGACTCGTCGACAACCACAATTTCTAACGGTCGTCATAAGATCCAGCCGTT
GAGATTTAACGATCGTTACGATTTATATTTTTTTAGCATTATCGTTTTATTTTTTAAAT
ATACGGTGGAGCTGAAAATTGGCAATAATTGAACCGTGGGTCCCACTGCATTGAAG
CGTATTTCGTATTTTCTAGAATTCTTCGTGCTTTATTTCTTTTCCTTTTTGTTTTTTTTT
GCCATTTATCTAATGCAAGTGGGCTTATAAAATCAGTGAATTTCTTGGAAAAGTAAC
TTCTTTATCGTATAACATATTGTGAAATTATCCATTTCTTTTAATTTTTTAGTGTTATT
GGATATTTTTGTATGATTATTGATTTGCATAGGATAATGACTTTTGTATCAAGTTGGT
GAACAAGTCTCGTTAAAAAAGGCAAGTGGTTTGGTGACTCGATTTATTCTTGTTATT
TAATTCATATATCAATGGATCTTATTTGGGGCCTGGTCCATATTTAACACTCGTGTT
CAGTCCAATGACCAATAATATTTTTTCATTAATAACAATGTAACAAGAATGATACACA
AAACATTCTTTGAATAAGTTCGCTATGAAGAAGGGAACTTATCCGGTCCTAGATCAT
CAGTTCATACAAACCTCCATAGAGTTCAACATCTTAAACAAGAATATCCTGATCTGA
AGAATGTGGAGGCTTTAGTCCCTTGGATACTTGGGAGGCTGTGGAAGAACAGAAA
CGAGCTGGTGCTTAAAGGGAGGGAATTTGGAACCAATGAGGTATTAGTAAGGACA
CAAGAAGATGCAGATGAGTGGATTAGAAGGAAAGAGGCTCAGAATGTAAGGAAAG
CACCAACCACAACACCGTCGGGACAGACGAGAAACATGGGAAGCTCCACCACAAT
CTTGGGTTAAGTGCAACTTTGATGGGGCATGGCCAACAGAAGGATTAAAATGTGG
CTTAGGGTGGGTGCTTCGCGATCATACAGGGAAGGTGTTATGGTTAGGTGCAAGA
GCTGTGGTAAAAGTAAGAAGCGTGCTGGAAGTAGAAGTGGAGGCTCTTAGATGGG
CTGTGCTGTCATTATCCCGATTCAATTATAGGAAGATCATTTTTGAGGTGGATTCTC
AGCAACTTGTGTCTTTGGTTACATGAAAGTTATGCTTGTCAAGTCTCAATCCAATTA
TCCAAGACATAAAGTATCTACTTAGCAAGTTTGAGGATTTTATGCTTGTGCATACAA
GCCGAGAAGGAAATGGAGTGGCAGATAGAATAGCTAAGGAATCTCTTTCTTTTGAG
AATTATGATCTAAAGTTGTATTCTATTGTACCAATTTGGGTTAAAAGCTCTGTTGAGC
TAGACTCATATGCATCCTAGGAAACAAGTTGTAATGAGTTGCTGGCCTCTCTTGGG
ATTGTTCATCAAAGTAGCTGTCCCTACACACCACAACAGAATGGTGTTGTGGAGAG
AAAGCACAGACACATCCTTGAGATGGCAAGGGCACTTAAGTTTCAGAGTGGTGTAC
CTACCAGGTTTTGGGGAGACTGTGTCAAGACTGTTGTATACCTGATCAATAGACTC
CCTACTCCAATCTTACAAGGCAAATGTCCATATGAACTACTCTATCAGAAACATGCC
AAGCTTGATCACTTGAGAGTGTTTGGGTGTCTTGCTTTTGCAGCAACACTGCCCAA
AGGTGACAAACTTGCACCTAGAGCCAAGCGAACCATCTTCATTGGTTATTCTGAGA
CTCAAAAGGGTTACAGGTTGTATGACTTGGATAATAAGGTGATCATAGTGAGCAGG
GATGTAGTTTTCAGAGAGTTTCAGTTCCCTTTCAAAGAGTCCATCTCCCATGAACCT
GATATGTTCACTCGAGCAGGTTCACTTAGCTCTGAAGATACAGCTTTGCAGCTAGT
GTATAATGATATTTTTCCTTCCAGCTTTCATACTGATGATGATGATACTCCAGTTCTT
ATTCCTGACATTGTAGAGGCCAATGAGGATATCACTTTGGGGACTCATTCTACCCA
AAATCATATCACTTCAGCTGATGCTGCACCTTCAGGCCACCTGCATGCTCCTGCAG
AGTCAGTTGAGTCAGCTGAGCCTGACCTAGCAAATGCTGAATTTGAGCACCATACT

GCTGTAGCTGATCCCTCTCTAGTTCCAGCAAACCTGCACACTAGACCTAAACGCAA
TGCAGGTCCTCCCATCTGGCTCAAGGACTTTGTGACACTGAACAAAGGCTCTAGG
GATGTTCCATACCCTATAGCCAACTATGTTTCCTATGATCACTTGCCTGTTCACTAT
CAAGCTTATTTGAGTGCTTTCTCCACTTATACTGAACCTAAATCCTTCAAAGAAGCA
GCTCAAGATGAGAAATGGATGGAGGCCATGTCCCTTGAGATACAGGCTCTTGAGG
ATAATAACACCTGGGAGATTGTCCCTTTACCCCCTGGTAAACAGCCTATAGGGTCC
AAATGGGTGTACAAAATTAAATACAAAGCTAATGGTGAAGTTGACAGGTTTAAGGC
AAGGCTAGTGGCCAAGGGATACACTCAGCAAGAAGGCCTTGACTACCATGAAACT
TTTTCTCCAGTGGCCAAAATGGTCACTGTAAAAGCTGTCATATCTGTTGCTGCTTCC
AAGGGCTGGTTCCTTTTCCAGATGGATGTCAACAATGCTTTTCTACAAGGTGACCT
CATGGAAGAAGTTTATATGTCTCTGCCTCAGGGTTTTCACAGCCAAGGGGAGACCA
AAGTGTGCAGGCTGTTGAATCCCTCTATGGTTTAAAGCAAGCATCAAGGCAGTGGA
ACATCAAGCTTACCACTGTCCTTATGCAGGCTGGTTTTATGCAAAGTGCTTATGATC
ACTCCTTGTTTACCAAAAGAGAGGGGACTGACCTTGTCATAATCCTGATTTATGTG
GATGATCTGTTAATAACAGGCAGCAGCAATGTTTTGATTTCAGAAGCAAAGGCAAC
CCTGCATCAGCATTTCAAGATGAAAGATTTGGGAGAACTAAAATACTTCTTAGGCAT
TGAGGTGCTGAGATCAGAAAAGGGAATCCTACTGAACCAGAGGAAGTATGCACTG
GAACTGATATCAGGTGTTGGTCTGGGAGGCTGCAGACCAGTGTCAACCCCAATGG
AACAGAACCAAAGGCTGACTACTGTAGAATATGACAAGCACTTAGGAAAGACTGAT
GATGCAGAGTTAGAGGATGTGGGTTCCTATCAGAGACTGGTGGGAAAGCTTCTCT
ACTTGACAATCACAAGGCCAGATATCAGCTTTGCAGTGCAGGTGCTGAGTCAATTC
ATGCAGCAACCCAAACAGTCACACCTGGAAGCAGCATTGAGGGTTGTGAAGTACA
TAAAAGGTTCTCCAGGTGTTAGGAAGCGGCCGCATGCATATGAGTCTAGCTCAACA
GAGCTTTTAACCCAAATTGGTACAATAGAATACAACTTTAGATCATAATTCTCAAAA
GAAAGAGATTCCTTAGCTATTCTATCTGCCACTCCATTTCCTTCTCGGCTTGTATGC
ACAAGCATAAAATCCTCAAACTTGCTAAGTAGATACTTTATGTCTTGGATAATTGGA
TTGAGACTTGACAAGCATAACTTTCATGTAACCAAAGACACAAGTTGCTGAGAATC
CACCTCAAAAATGATCTTCCTATAATTGAATCGGGATAATGACAGCACAGCCCATCT
AAGAGCCTCCACTTCTACTTCCAGCACGCTTCTTACTTTTACCACAGCTCTTGCACC
TAACCATAACACCTTCCCTGTATGATCGCGAAGCACCCACCCTAAGCCACATTTTA
ATCCTTCTGTTGGCCATGCCCCATCAAAGTTGCACTTAACCCAAGATTGTGGTGGA
GCTTCCCATGTTTCTCGTCTGTCCCGACGGTGTTGTGGTTGGTGCTTTCCTTACAT
TCTGAGCCTCTTTCCTTCTAATCCACTCATCTGCATCTTCTTGTGTCCTTACTAATAC
CTCATTGGTTCCAAATTCCCTCCCTTTAAGCACCAGCTCGTTTCTGTTCTTCCACAG
CCTCCCAAGTATCCAAGGGACTAAAGCCTCCACATTCTTCAGATCAGGATATTCTT
GTTTAAGATGTTGAACTCTATGGAGGTTTGTATGAACTGATGATCTAGGACCGGAT
AAGTTCCCTTCTTCATAGCGAACTTATTCAAAGAATGTTTTGTGTATCATTCTTGTTA
CATTGTTATTAATGAAAAAATATTATTGGTCATTGGACTGAACACGAGTGTTAAATAT
GGACCAGGCCCCAAATAAGATCCATTGATATATGAATTAAATAACAAGAATAAATCG
AGTCACCAAACCACTTGCCTTTTTTAACGAGACTTGTTCACCAACTTGATACAAAAG
TCATTATCCTATGCAAATCAATAATCATACAAAAATATCCAATAACACTAAAAAATTA
AAAGAAATGGATAATTTCACAATATGTTATACGATAAAGAAGTTACTTTTCCAAGAAA
TTCACTGATTTTATAAGCCCACTTGCATTAGATAAATGGCAAAAAAAAACAAAAAGG
AAAAGAAATAAAGCACGAAGAATTCTAGAAAATACGAAATACGCTTCAATGCAGTG
GGACCCACGGTTCAATTATTGCCAATTTTCAGCTCCACCGTATATTTAAAAAATAAA
ACGATAATGCTAAAAAAATATAAATCGTAACGATCGTTAAATCTCAACGGCTGGATC

TTATGACGACCGTTAGAAATTGTGGTTGTCGACGAGTCAGTAATAAACGGCGTCAA
AGTGGTTGCAGCCGGCACACACGAGTCGTGTTTATCAACTCAAAGCACAAATACTT
TTCCTCAACCTAAAAATAAGGCAATTAGCCAAAAACAACTTTGCGTGTAAACAACGC
TCAATACACGTGTCATTTTATTATTAGCTATTGCTTCACCGCCTTAGCTTTCTCGTGA
CCTAGTCGTCCTCGTCTTTTCTTCTTCTTCTTCTATAAAACAATACCCAAAGAGCTCT
TCTTCTTCACAATTCAGATTTCAATTTCTCAAAATCTTAAAAACTTTCTCTCAATTCTC
TCTACCGTGATCAAGGTAAATTTCTGTGTTCCTTATTCTCTCAAAATCTTCGATTTTG
TTTTCGTTCGATCCCAATTTCGTATATGTTCTTTGGTTTAGATTCTGTTAATCTTAGA
TCGAAGACGATTTTCTGGGTTTGATCGTTAGATATCATCTTAATTCTCGATTAGGGT
TTCATAGATATCATCCGATTTGTTCAAATAATTTGAGTTTTGTCGAATAATTACTCTT
CGATTTGTGATTTCTATCTAGATCTGGTGTTAGTTTCTAGTTTGTGCGATCGAATTT
GTCGATTAATCTGAGTTTTTCTGATTAACAGGGATCATCAACAAGTTTGTACAAAAA
AGCAGGCTCTTTAAAGTATTTTTACAACAATTACCAACAACAACAAACAACAAACAA
CATTACAATTACTATTTACAATTACAAAAAAAGTTAACATGGACAAGAAGTACAGCA
TCGGCCTGGCCATCGGCACCAACTCTGTGGGCTGGGCCGTGATCACCGACGAGT
ACAAGGTGCCCAGCAAGAAATTCAAGGTGCTGGGCAACACCGACCGGCACAGCAT
CAAGAAGAACCTGATCGGCGCCCTGCTGTTCGACAGCGGAGAAACAGCCGAGGC
CACCCGGCTGAAGAGAACCGCCAGAAGAAGATACACCAGACGGAAGAACCGGAT
CTGCTATCTGCAAGAGATCTTCAGCAACGAGATGGCCAAGGTGGACGACAGCTTC
TTCCACAGACTGGAAGAGTCCTTCCTGGTGGAAGAGGATAAGAAGCACGAGCGGC
ACCCCATCTTCGGCAACATCGTGGACGAGGTGGCCTACCACGAGAAGTACCCCAC
CATCTACCACCTGAGAAAGAAACTGGTGGACAGCACCGACAAGGCCGACCTGCGG
CTGATCTATCTGGCCCTGGCCCACATGATCAAGTTCCGGGGCCACTTCCTGATCG
AGGGCGACCTGAACCCCGACAACAGCGACGTGGACAAGCTGTTCATCCAGCTGGT
GCAGACCTACAACCAGCTGTTCGAGGAAAACCCCATCAACGCCAGCGGCGTGGAC
GCCAAGGCCATCCTGTCTGCCAGACTGAGCAAGAGCAGACGGCTGGAAAATCTGA
TCGCCCAGCTGCCCGGCGAGAAGAAGAATGGCCTGTTCGGCAACCTGATTGCCCT
GAGCCTGGGCCTGACCCCCAACTTCAAGAGCAACTTCGACCTGGCCGAGGATGC
CAAACTGCAGCTGAGCAAGGACACCTACGACGACGACCTGGACAACCTGCTGGCC
CAGATCGGCGACCAGTACGCCGACCTGTTTCTGGCCGCCAAGAACCTGTCCGACG
CCATCCTGCTGAGCGACATCCTGAGAGTGAACACCGAGATCACCAAGGCCCCCCT
GAGCGCCTCTATGATCAAGAGATACGACGAGCACCACCAGGACCTGACCCTGCTG
AAAGCTCTCGTGCGGCAGCAGCTGCCTGAGAAGTACAAAGAGATTTTCTTCGACC
AGAGCAAGAACGGCTACGCCGGCTACATCGATGGCGGAGCCAGCCAGGAAGAGT
TCTACAAGTTCATCAAGCCCATCCTGGAAAAGATGGACGGCACCGAGGAACTGCT
CGTGAAGCTGAACAGAGAGGACCTGCTGCGGAAGCAGCGGACCTTCGACAACGG
CAGCATCCCCCACCAGATCCACCTGGGAGAGCTGCACGCCATTCTGCGGCGGCA
GGAAGATTTTTACCCATTCCTGAAGGACAACCGGGAAAAGATCGAGAAGATCCTGA
CCTTCCGCATCCCCTACTACGTGGGCCCTCTGGCCAGGGGAAACAGCAGATTCGC
CTGGATGACCAGAAAGAGCGAGGAAACCATCACCCCCTGGAACTTCGAGGAAGTG
GTGGACAAGGGCGCCAGCGCCCAGAGCTTCATCGAGCGGATGACCAACTTCGAT
AAGAACCTGCCCAACGAGAAGGTGCTGCCCAAGCACAGCCTGCTGTACGAGTACT
TCACCGTGTACAACGAGCTGACCAAAGTGAAATACGTGACCGAGGGAATGAGAAA
GCCCGCCTTCCTGAGCGGCGAGCAGAAAAAGGCCATCGTGGACCTGCTGTTCAAG
ACCAACCGGAAAGTGACCGTGAAGCAGCTGAAAGAGGACTACTTCAAGAAAATCG
AGTGCTTCGACTCCGTGGAAATCTCCGGCGTGGAAGATCGGTTCAACGCCTCCCT

GGGCACATACCACGATCTGCTGAAAATTATCAAGGACAAGGACTTCCTGGACAATG
AGGAAAACGAGGACATTCTGGAAGATATCGTGCTGACCCTGACACTGTTTGAGGA
CAGAGAGATGATCGAGGAACGGCTGAAAACCTATGCCCACCTGTTCGACGACAAA
GTGATGAAGCAGCTGAAGCGGCGGAGATACACCGGCTGGGGCAGGCTGAGCCG
GAAGCTGATCAACGGCATCCGGGACAAGCAGTCCGGCAAGACAATCCTGGATTTC
CTGAAGTCCGACGGCTTCGCCAACAGAAACTTCATGCAGCTGATCCACGACGACA
GCCTGACCTTTAAAGAGGACATCCAGAAAGCCCAGGTGTCCGGCCAGGGCGATAG
CCTGCACGAGCACATTGCCAATCTGGCCGGCAGCCCCGCCATTAAGAAGGGCATC
CTGCAGACAGTGAAGGTGGTGGACGAGCTCGTGAAAGTGATGGGCCGGCACAAG
CCCGAGAACATCGTGATCGAAATGGCCAGAGAGAACCAGACCACCCAGAAGGGA
CAGAAGAACAGCCGCGAGAGAATGAAGCGGATCGAAGAGGGCATCAAAGAGCTG
GGCAGCCAGATCCTGAAAGAACACCCCGTGGAAAACACCCAGCTGCAGAACGAGA
AGCTGTACCTGTACTACCTGCAGAATGGGCGGGATATGTACGTGGACCAGGAACT
GGACATCAACCGGCTGTCCGACTACGATGTGGACGCTATCGTGCCTCAGAGCTTT
CTGAAGGACGACTCCATCGATAACAAAGTGCTGACTCGGAGCGACAAGAACCGGG
GCAAGAGCGACAACGTGCCCTCCGAAGAGGTCGTGAAGAAGATGAAGAACTACTG
GCGCCAGCTGCTGAATGCCAAGCTGATTACCCAGAGGAAGTTCGACAATCTGACC
AAGGCCGAGAGAGGCGGCCTGAGCGAACTGGATAAGGCCGGCTTCATCAAGAGA
CAGCTGGTGGAAACCCGGCAGATCACAAAGCACGTGGCACAGATCCTGGACTCCC
GGATGAACACTAAGTACGACGAGAACGACAAACTGATCCGGGAAGTGAAAGTGAT
CACCCTGAAGTCCAAGCTGGTGTCCGATTTCCGGAAGGATTTCCAGTTTTACAAAG
TGCGCGAGATCAACAACTACCACCACGCCCACGACGCCTACCTGAACGCCGTCGT
GGGAACCGCCCTGATCAAAAAGTACCCTAAGCTGGAAAGCGAGTTCGTGTACGGC
GACTACAAGGTGTACGACGTGCGGAAGATGATCGCCAAGAGCGAGCAGGAAATC
GGCAAGGCTACCGCCAAGTACTTCTTCTACAGCAACATCATGAACTTTTTCAAGAC
CGAGATTACCCTGGCCAACGGCGAGATCCGGAAGCGGCCTCTGATCGAGACAAA
CGGCGAAACAGGCGAGATCGTGTGGGATAAGGGCCGGGACTTTGCCACCGTGCG
GAAAGTGCTGTCTATGCCCCAAGTGAATATCGTGAAAAAGACCGAGGTGCAGACA
GGCGGCTTCAGCAAAGAGTCTATCCTGCCCAAGAGGAACAGCGACAAGCTGATCG
CCAGAAAGAAGGACTGGGACCCTAAGAAGTACGGCGGCTTCGACAGCCCCACCG
TGGCCTATTCTGTGCTGGTGGTGGCCAAAGTGGAAAAGGGCAAGTCCAAGAAACT
GAAGAGTGTGAAAGAGCTGCTGGGGATCACCATCATGGAAAGAAGCAGCTTCGAG
AAGAATCCCATCGACTTTCTGGAAGCCAAGGGCTACAAAGAAGTGAAAAAGGACCT
GATCATCAAGCTGCCTAAGTACTCCCTGTTCGAGCTGGAAAACGGCCGGAAGAGA
ATGCTGGCCTCTGCCGGCGAACTGCAGAAGGGAAACGAACTGGCCCTGCCCTCC
AAATATGTGAACTTCCTGTACCTGGCCAGCCACTATGAGAAGCTGAAGGGCTCCC
CCGAGGATAATGAGCAGAAACAGCTGTTTGTGGAACAGCACAAACACTACCTGGA
CGAGATCATCGAGCAGATCAGCGAGTTCTCCAAGAGAGTGATCCTGGCCGACGCT
AATCTGGACAAGGTGCTGAGCGCCTACAACAAGCACAGAGACAAGCCTATCAGAG
AGCAGGCCGAGAATATCATCCACCTGTTTACCCTGACCAATCTGGGAGCCCCTGC
CGCCTTCAAGTACTTTGACACCACCATCGACCGGAAGAGGTACACCAGCACCAAA
GAGGTGCTGGACGCCACCCTGATCCACCAGAGCATCACCGGCCTGTACGAGACA
CGGATCGACCTGTCTCAGCTGGGAGGCGACGCCTATCCCTATGACGTGCCCGATT
ATGCCAGCCTGGGCAGCGGCTCCCCCAAGAAAAAACGCAAGGTGGAAGATCCTAA
GAAAAAGCGGAAAGTGGAAGATGCTCCAAAGAAGAAGAGAAAGGTCGACGGCATT
GGTAGTGGGAGCAACGGCAGCAGCGGATCCAACGGTCCGACTGACGCCGCGGAA

GAAGAACTTTTGAGCAAGAATTATCATCTTGAGAACGAAGTGGCTCGTCTTAAGAA
AGGTTCTGGCAGTGGAGGTTCTGGCTCCGGATCTGGTGGTTCGGGCTCAGGCGG
GTCCGGATCAGGCGAAGAACTGCTTTCAAAGAATTACCACCTGGAAAATGAGGTA
GCTAGACTGAAAAAGGGAGCGGAAGTGGGGGCTCCGGGTCGGGCTCAGGGGG
CTCCGGTTCGGGAGGCTCAGGGTCGGGGGAGGAGTTGCTGAGCAAAAATTATCAT
TTGGAGAACGAAGTAGCACGACTAAAGAAAGGGTCCGGATCGGGTGGTTCAGGAT
CTGGATCCGGAGGATCAGGGTCCGGTGGGTCGGGCTCAGGAGAGGAGTTACTCT
CGAAAAATTATCATCTCGAAAACGAAGTGGCTCGGCTAAAAAAGGGCAGTGGTTCT
GGAGGATCTGGGTCGGGGTCAGGCGGGTCTGGATCTGGGGGATCTGGATCTGGT
GAAGAGCTATTATCTAAAAACTACCACCTCGAAAATGAGGTGGCACGCTTAAAAAA
GGGAAGTGGCAGTGGTGGGTCGGGATCTGGCTCTGGTGGCTCAGGCTCGGGAGG
TTCAGGTTCCGGGGAAGAGCTACTATCCAAGAATTATCATCTTGAGAACGAGGTAG
CGCGTTTGAAGAAGGGTTCCGGCTCAGGAGGATCTGGGTCAGGGTCAGGGGGTT
CCGGGTCAGGCGGGTCCGGGTCAGGCGAGGAACTGCTCTCGAAGAACTATCATC
TTGAAAATGAGGTCGCTCGATTAAAAAAGGGATCGGGCAGTGGTGGGTCCGGCTC
CGGTTCCGGAGGATCGGGATCTGGGGGCTCGGGATCCGGGGAGGAACTACTTTC
AAAGAATTACCACCTCGAAAACGAAGTAGCTCGATTAAAGAAAGGTTCAGGGTCGG
GTGGCTCAGGTTCGGGATCAGGTGGGTCAGGCTCCGGTGGTTCAGGTTCGGGAG
AAGAATTACTGAGTAAAAATTATCATCTGGAAAATGAGGTAGCGAGACTAAAAAAG
GGGAGTGGTTCTGGCGGTTCGGGATCTGGCTCTGGGGGCTCTGGGTCGGGAGGG
TCTGGGTCTGGCGAGGAATTGCTATCGAAAAATTATCATCTTGAGAACGAAGTTGC
TAGGCTCAAAAAGGGCTCAGGCTCAGGCGGGTCCGGGTCAGGGTCGGGAGGTTC
CGGATCCGGGGGATCAGGCTCAGGGTAACCCGGGGGCGCGCCATGATAACTAGA
GTCCTGCTTTAATGAGATATGCGAGACGCCTATGATCGCATGATATTTGCTTTCAAT
TCTGTTGTGCACGTTGTAAAAAACCTGAGCATGTGTAGCTCAGATCCTTACCGCCG
GTTTCGGTTCATTCTAATGAATATATCACCCGTTACTATCGTATTTTTATGAATAATA
TTCTCCGTTCAATTTACTGATTGTACCCTACTACTTATATGTACAATATTAAAATGAA
AACAATATATTGTGCTGAATAGGTTTATAGCGACATCTATGATAGAGCGCCACAATA
ACAAACAATTGCGTTTTATTATTACAAATCCAATTTTAAAAAAAGCGGCAGAACCGG
TCAAACCTAAAAGACTGATTACATAAATCTTATTCAAATTTCAAAAGTGCCCCAGGG
GCTAGTATCTACGACACACCGAGCGGCGAACTAATAACGCTCACTGAAGGGAACT
CCGGTTCCCCGCCGGCGCGCATGGGTGAGATTCCTTGAAGTTGAGTATTGGCCGT
CCGCTCTACCGAAAGTTACGGGCACCATTCAACCCGGTCCAGCACGGCGGCCGG
GTAACCGACTTGCTGCCCCGAGAATTATGCAGCATTTTTTTGGTGTATGTGGGCCC
CAAATGAAGTGCAGGTCAAACCTTGACAGTGACGACAAATCGTTGGGCGGGTCCA
GGGCGAATTTTGCGACAACATGTCGAGGCTCAGCAGGACCGGCATGCAAGCTAGC
TTACTAGTGATATTCTATAGTGTCACCTAAATCTGCGGC

SunTagng-22aa
NOS term
TET1cd
2xNLS
Linker
1xHA

sfGFP
scFV
UBQ10
Insulator
Omega RBC
dCAS9
3xNLS
GCN4 10x 22aa
OCS term

CACGGGGTGGTTTGGTTTAAACGTAACATAGATGACACCGCGCGATAATTTATC
CTAGTTTGCGCGCTATATTTTGTTTTCTATCGCGTATTAAATGTATAATTGCGGGAC
TCTAATCATAAAAACCCATCTCATAAATAACGTCATGCATTACATGTTAATTATTACA
TGCTTAACGTAATTCAACAGAAATTATATGATAATCATCGCAAGACCGGCAACAGG
ATTCAATCTTAAGAAACTTTATTGCCAAATGTTTGAACGATCGGGGAAATTCGAGCT
CAAACTTACGGCCATGGCGTAGGCGGCCTTTTCCGTTGTGATCTTCGCGGTCTCCT
CCTTGTTTTTGGACTAGTACCACCACCGCTACCACCACCTTCGGTTACCGTGAAGG
TTTTGGTAGCGTCGTCGTAGGTCCATTCACCGTCAACACCGTTGTCGTTAGCGTAC
TGTTTGAAAACTTTTTCCGCGGTAGCAGCGTCAACAGCTTCGGTGGTGGTTTCACC
TTTCAGGGTTTTACCGTTCAGGATAAGCTTGTACTCTTCGGTCCGAGATCCTCCTC
CCGTACGTCAGACCCAATGGTTATAGGGCCCCGCAACGTGTGTGAGAGCATAAGG
GGACACGGTGACAACATTGTCATGGGTTAATGTTAATGCTTTATGAGAAGGAATTT
GGTTCAATTCATTTACTTCAGAGGACTGTTCTGGACCTTCATTAGCTGCCTGGTCTT
TTTGCTCTGAGGCCTTCATTTTCTTATTCTTAGCTTCTTTAGCCTCAAACTTAATCTT
GTTTAGTTCAAAACCATGTTGGGGCTTATTTAGGTTTTTGTGCTGGTAAAAGACAAG
GGAGAGGCGGGTTGGATGATTACGGTTGGGGTGCTCAACAGGAGTGGTAGCGTG
CAGCTCTCGCCGGGCACACTCAATCAAAACCGAGCCGTGAGCAGGTGCGATGGC
CACCCCACCAATATTTGCATCCAAAAAGATGTGCTCACTGTCTGACCAATACTCATC
AATGTGGGGCAATTTCTCCTCAGCAGGTGACAGGGGGTCATCAGATAGGGGTTCG
TCTGATGGAGGCTCATCTGCTTCAGAATGCTGCTCATCTTCTTCCATTGGAGAAGA
GGCAAGGTCTTGAGGAGAGGTGAGGAAGGAGGGCTGGTGGTTTGGCTGATGAGG
CGTTAGCGGCTCAGTCACACCAGTGGAAGGCTCAGAATTAATGAGGGGCTCCATC
ACAGGAGCAGACAGGGTGGGGAGAGGAGCCACTTCGCCAAGCTGTGAAATGCCA
GGGCCATCAGCAGCTGCAGCATTGGCACCACTGAGTCTTCCCGAAGGCATCGTAC
AGTGGGGAGTGCTGCTTCTTTCTGAAAACCCGCATGAGGCTGTTGCGTCATTCTTC
AGTGGAGCTGGTGTGGCTGAAGCAGTCTTCGGGGACCAGGAGAAGCCTGGAGAT
GCCTCTTTCACTGGGTGAGGAGCGGATGGCATCAGCGAATAAGTTTTAGTGTTGTC
TGAACTTTTTAAGATAAAATGGGGTTCGGTTTCACTTTTTACTTCAGGTTGCACGGT
CTCAGTGTTACTCCCTAAGGTTGGCAGTGACGAAGGCTTACTGTTGTTTGTTGTTG
TTGAGTTATTCTTCCGCTTGATTCGGGGAATAGGTTTCTTTTCCACTGCCCTTATCT
TATGTGCAAGAACCTCTGTCATCATCGCAGCCCTCTTCTTTCCAGAACGGGGAACA
GGCTGAGTGAAACACGTTCTTTTTTTGCGGCGGGGTGCCAGGACCTCGATGGCCC
CAGATTTGATCTTGGCTTCCATTCCTTCCTTGGAGCCAAACTCATCTGTGTCTGAAA
GCTTATAAAGAGGTAGCACATGGAGCTGCTCATCTTGAGGAATAACACCCAAAGAG

CGGTTATCTTCTCGAGTTAAGGTACAAACCACAGTGCTTCCATTATTCATGTTGTGA
ATGTCCCTGTGGGGATGAGCACAGAAGTCCAGGCAAGCAGTGACCCCAGAGAAG
GGTCGACCTTCCTTGCTGCCAAGCCGACATTCTCGGGCAACATTTTCATATTCCAC
CTGATTTTGGTAAGCTACTGGAGCATACTGCTTATAAATTGGAGCTAATCGTGTAGC
CAAACTCTGTAAGTTATCTTCAAGGTTTTTTTCATGTAAGGGAGAGCTTGGATCAAT
TCTAAATCTTCTGGGGCTTGGGCTTCTACCAAACTTACAGCCATTAAAGTACATACT
CCATGAACAGCCAAAAGAGAATGAAGCTCCACAAGTCTCTGGATCAATTCCTTGAC
ATGTACAGGTACGATTTTCATTGAGGGTGCATCTTCTGTCGGTAGGGTGCCCATTG
TATGACTTTAGATTCTCTGTGAGCTCTGTGTATAGCCGGTCGGCCATTGGAAGAGG
GATGCCATCCCACACCATGATGAGCACCACCATCACAGCAGTTGGACAGTGGTGG
CCTGTACGCTGCCGGACCAAACAAAGAACTTTTTCTTCATCACTGCTTCTTCTTAAA
ACCCACTTAGCAATTGGACACCCATGAGAGCTTTTCCCTTCTTTACCGGTGTACAC
TACTATTTCTATCCTTATTGCGTTTCCTTTTTGACCATACCTATTCTCCATGATTTCC
CTGACAGCAGCAACACTTGGTCCTGCCCCAAGGTGTGTATAATATGGGCCTTTGTC
TTTTTGTATAACTCGATCAAGACAGCTGCAGGTGGGCAGCCCGGATCCGCCACCG
CCCACTTTCCGCTTTTTCTTAGGAGCTTCCACCTTGCGTTTTTTCTTGGGAGCGCC
GCTGCCCAGGCTGGCATAATCGGGCACGTCATAGGGATACGTACGACCGGTCCG
ACCTCCACCTTTGTAGAGCTCATCCATGCCATGTGTAATCCCAGCAGCAGTTACAA
ACTCAAGAAGGACCATGTGGTCACGCTTTTCGTTGGGATCTTTCGAAAGGACAGAT
TGTGTCGACAGGTAATGGTTGTCTGGTAAAAGGACAGGGCCATCGCCAATTGGAG
TATTTTGTTGATAATGGTCTGCTAGTTGAACGGAACCATCTTCAACGTTGTGGCGAA
TTTTGAAGTTAGCTTTGATTCCATTCTTTTGTTTGTCTGCCGTGATGTATACATTGTG
TGAGTTAAAGTTGTACTCGAGTTTGTGTCCAAGAATGTTTCCATCTTCTTTAAAATCA
ATACCCTTTAACTCGATACGATTAACAAGGGTATCACCTTCAAACTTGACTTCAGCA
CGCGTCTTGTAGGTCCCGTCATCTTTGAAAGATATAGTGCGTTCCTGTACATAACC
TTCGGGCATGGCACTCTTGAAAAAGTCATGCCGTTTCATGTGATCCGGATAACGGG
AAAAGCATTGAACACCATAGGTCAGAGTAGTGACAAGTGTTGGCCACGGAACAGG
TAGTTTTCCAGTAGTGCAAATAAATTTAAGGGTGAGTTTTCCGTTTGTAGCATCACC
TTCACCCTCTCCACGGACAGAAAATTTGTGCCCATTAACATCACCATCTAATTCAAC
AAGAATTGGGACAACTCCAGTGAAAAGTTCTTCTCCTTTGCTACCGCTTCCACCTC
CACCTGGATCCAAGCTTCCGCCGCCTCCTGAACCACCACCGCCACTACCTCCTCC
CCCAGAACCTCCGCCTCCACCAGCGTAATCTGGAACATCGTATGGGTAGCTGCTC
ACGGTCACCAGGGTGCCCTGGCCCCAGTAGTCGAACAGGCCGGTCACGCAGTAG
TACAGGGCGGTGTCGTCGCTGCGCACCTTGCTCATCTGCAGGTACACGGTGTTCT
TGCCGTTGTCCTTGCTGATGATGAAGCGGTCCTTCAGGGCGCTGTTGTAGTCGGT
GATGCCGTCGCCCCAGATCACGCCGATCCACTCCAGGCCGCGGCCGGGGGCCTG
GCGCACCCAGTTCACGCCGTAGTCGGTCAGGCTGAAGCCGCTCACGGCGCAGCT
CAGCTTCAGGCTGCCGCCGGGCTGCACCAGGCCGCCGCCGCTCTCCAGCAGCTT
CACCTCGCTGCCGCCGCCGCTGCTGCCGCCGCCGCCGCTGCCGCCGCCGCCGC
TGCCGCCGCCGCCGCGCTTCAGCTCCACCTTGGTGCCCTGGCCGAACACCCAGT
GGTTGCTGTACCACAGGGCGCAGAAGTAGGTGGCGAAGTCCTCGGGCTGCAGGC
TGCTGATGGTCAGGGTGGCCTTGTCGCCGATCAGGCTGCCGCTGAAGCGGCTGG
GCACGCCGGGGGCGCGGTTGTTGGTGCCGCCGATCAGGCCCTTGAACAGCTTGC
CGGGCTTCTCCTGCACCCAGCTGGCGTAGTTGCTGGTGGTCACGGCGCCGGTGC
TGCTGCGGCAGGTGATGGTCACGCGGTCGCCCACGCTGGCGCTCAGGCTGCTGG
GGCTCTGGGTCATCACGATGTCGGGGCCCATAAACCCTAGGTGTTAATCAGAAAA

ACTCAGATTAATCGACAAATTCGATCGCACAAACTAGAAACTAACACCAGATCTAGA
TAGAAATCACAAATCGAAGAGTAATTATTCGACAAAACTCAAATTATTTGAACAAAT
CGGATGATATCTATGAAACCCTAATCGAGAATTAAGATGATATCTAACGATCAAACC
CAGAAAATCGTCTTCGATCTAAGATTAACAGAATCTAAACCAAAGAACATATACGAA
ATTGGGATCGAACGAAAACAAAATCGAAGATTTTGAGAGAATAAGGAACACAGAAA
TTTACCTTGATCACGGTAGAGAGAATTGAGAGAAAGTTTTTAAGATTTTGAGAAATT
GAAATCTGAATTGTGAAGAAGAAGAGCTCTTTGGGTATTGTTTTATAGAAGAAGAA
GAAGAAAAGACGAGGACGACTAGGTCACGAGAAAGCTAAGGCGGTGAAGCAATAG
CTAATAATAAAATGACACGTGTATTGAGCGTTGTTTACACGCAAAGTTGTTTTTGGC
TAATTGCCTTATTTTTAGGTTGAGGAAAAGTATTTGTGCTTTGAGTTGATAAACACG
ACTCGTGTGTGCCGGCTGCAACCACTTTGACGCCGTTTATTACTGACTCGTCGACA
ACCACAATTTCTAACGGTCGTCATAAGATCCAGCCGTTGAGATTTAACGATCGTTA
CGATTTATATTTTTTTAGCATTATCGTTTTATTTTTTAAATATACGGTGGAGCTGAAA
ATTGGCAATAATTGAACCGTGGGTCCCACTGCATTGAAGCGTATTTCGTATTTTCTA
GAATTCTTCGTGCTTTATTTCTTTTCCTTTTTGTTTTTTTTTGCCATTTATCTAATGCA
AGTGGGCTTATAAAATCAGTGAATTTCTTGGAAAAGTAACTTCTTTATCGTATAACA
TATTGTGAAATTATCCATTTCTTTTAATTTTTTAGTGTTATTGGATATTTTTGTATGAT
TATTGATTTGCATAGGATAATGACTTTTGTATCAAGTTGGTGAACAAGTCTCGTTAA
AAAAGGCAAGTGGTTTGGTGACTCGATTTATTCTTGTTATTTAATTCATATATCAATG
GATCTTATTTGGGGCCTGGTCCATATTTAACACTCGTGTTCAGTCCAATGACCAATA
ATATTTTTTCATTAATAACAATGTAACAAGAATGATACACAAAACATTCTTTGAATAA
GTTCGCTATGAAGAAGGGAACTTATCCGGTCCTAGATCATCAGTTCATACAAACCT
CCATAGAGTTCAACATCTTAAACAAGAATATCCTGATCTGAAGAATGTGGAGGCTTT
AGTCCCTTGGATACTTGGGAGGCTGTGGAAGAACAGAAACGAGCTGGTGCTTAAA
GGGAGGGAATTTGGAACCAATGAGGTATTAGTAAGGACACAAGAAGATGCAGATG
AGTGGATTAGAAGGAAAGAGGCTCAGAATGTAAGGAAAGCACCAACCACAACACC
GTCGGGACAGACGAGAAACATGGGAAGCTCCACCACAATCTTGGGTTAAGTGCAA
CTTTGATGGGGCATGGCCAACAGAAGGATTAAAATGTGGCTTAGGGTGGGTGCTT
CGCGATCATACAGGGAAGGTGTTATGGTTAGGTGCAAGAGCTGTGGTAAAAGTAA
GAAGCGTGCTGGAAGTAGAAGTGGAGGCTCTTAGATGGGCTGTGCTGTCATTATC
CCGATTCAATTATAGGAAGATCATTTTTGAGGTGGATTCTCAGCAACTTGTGTCTTT
GGTTACATGAAAGTTATGCTTGTCAAGTCTCAATCCAATTATCCAAGACATAAAGTA
TCTACTTAGCAAGTTTGAGGATTTTATGCTTGTGCATACAAGCCGAGAAGGAAATG
GAGTGGCAGATAGAATAGCTAAGGAATCTCTTTCTTTTGAGAATTATGATCTAAAGT
TGTATTCTATTGTACCAATTTGGGTTAAAAGCTCTGTTGAGCTAGACTCATATGCAT
CCTAGGAAACAAGTTGTAATGAGTTGCTGGCCTCTCTTGGGATTGTTCATCAAAGT
AGCTGTCCCTACACACCACAACAGAATGGTGTTGTGGAGAGAAAGCACAGACACA
TCCTTGAGATGGCAAGGGCACTTAAGTTTCAGAGTGGTGTACCTACCAGGTTTTGG
GGAGACTGTGTCAAGACTGTTGTATACCTGATCAATAGACTCCCTACTCCAATCTTA
CAAGGCAAATGTCCATATGAACTACTCTATCAGAAACATGCCAAGCTTGATCACTT
GAGAGTGTTTGGGTGTCTTGCTTTTGCAGCAACACTGCCCAAAGGTGACAAACTTG
CACCTAGAGCCAAGCGAACCATCTTCATTGGTTATTCTGAGACTCAAAAGGGTTAC
AGGTTGTATGACTTGGATAATAAGGTGATCATAGTGAGCAGGGATGTAGTTTTCAG
AGAGTTTCAGTTCCCTTTCAAAGAGTCCATCTCCCATGAACCTGATATGTTCACTCG
AGCAGGTTCACTTAGCTCTGAAGATACAGCTTTGCAGCTAGTGTATAATGATATTTT
TCCTTCCAGCTTTCATACTGATGATGATGATACTCCAGTTCTTATTCCTGACATTGT

AGAGGCCAATGAGGATATCACTTTGGGGACTCATTCTACCCAAAATCATATCACTT
CAGCTGATGCTGCACCTTCAGGCCACCTGCATGCTCCTGCAGAGTCAGTTGAGTC
AGCTGAGCCTGACCTAGCAAATGCTGAATTTGAGCACCATACTGCTGTAGCTGATC
CCTCTCTAGTTCCAGCAAACCTGCACACTAGACCTAAACGCAATGCAGGTCCTCCC
ATCTGGCTCAAGGACTTTGTGACACTGAACAAAGGCTCTAGGGATGTTCCATACCC
TATAGCCAACTATGTTTCCTATGATCACTTGCCTGTTCACTATCAAGCTTATTTGAGT
GCTTTCTCCACTTATACTGAACCTAAATCCTTCAAAGAAGCAGCTCAAGATGAGAAA
TGGATGGAGGCCATGTCCCTTGAGATACAGGCTCTTGAGGATAATAACACCTGGG
AGATTGTCCCTTTACCCCCTGGTAAACAGCCTATAGGGTCCAAATGGGTGTACAAA
ATTAAATACAAAGCTAATGGTGAAGTTGACAGGTTTAAGGCAAGGCTAGTGGCCAA
GGGATACACTCAGCAAGAAGGCCTTGACTACCATGAAACTTTTTCTCCAGTGGCCA
AAATGGTCACTGTAAAAGCTGTCATATCTGTTGCTGCTTCCAAGGGCTGGTTCCTTT
TCCAGATGGATGTCAACAATGCTTTTCTACAAGGTGACCTCATGGAAGAAGTTTATA
TGTCTCTGCCTCAGGGTTTTCACAGCCAAGGGGAGACCAAAGTGTGCAGGCTGTT
GAATCCCTCTATGGTTTAAAGCAAGCATCAAGGCAGTGGAACATCAAGCTTACCAC
TGTCCTTATGCAGGCTGGTTTTATGCAAAGTGCTTATGATCACTCCTTGTTTACCAA
AAGAGAGGGGACTGACCTTGTCATAATCCTGATTTATGTGGATGATCTGTTAATAA
CAGGCAGCAGCAATGTTTTGATTTCAGAAGCAAAGGCAACCCTGCATCAGCATTTC
AAGATGAAAGATTTGGGAGAACTAAAATACTTCTTAGGCATTGAGGTGCTGAGATC
AGAAAAGGGAATCCTACTGAACCAGAGGAAGTATGCACTGGAACTGATATCAGGT
GTTGGTCTGGGAGGCTGCAGACCAGTGTCAACCCCAATGGAACAGAACCAAAGGC
TGACTACTGTAGAATATGACAAGCACTTAGGAAAGACTGATGATGCAGAGTTAGAG
GATGTGGGTTCCTATCAGAGACTGGTGGGAAAGCTTCTCTACTTGACAATCACAAG
GCCAGATATCAGCTTTGCAGTGCAGGTGCTGAGTCAATTCATGCAGCAACCCAAAC
AGTCACACCTGGAAGCAGCATTGAGGGTTGTGAAGTACATAAAAGGTTCTCCAGGT
GTTAGGAAGCGGCCGCATGCATATGAGTCTAGCTCAACAGAGCTTTTAACCCAAAT
TGGTACAATAGAATACAACTTTAGATCATAATTCTCAAAAGAAAGAGATTCCTTAGC
TATTCTATCTGCCACTCCATTTCCTTCTCGGCTTGTATGCACAAGCATAAAATCCTC
AAACTTGCTAAGTAGATACTTTATGTCTTGGATAATTGGATTGAGACTTGACAAGCA
TAACTTTCATGTAACCAAAGACACAAGTTGCTGAGAATCCACCTCAAAAATGATCTT
CCTATAATTGAATCGGGATAATGACAGCACAGCCCATCTAAGAGCCTCCACTTCTA
CTTCCAGCACGCTTCTTACTTTTACCACAGCTCTTGCACCTAACCATAACACCTTCC
CTGTATGATCGCGAAGCACCCACCCTAAGCCACATTTTAATCCTTCTGTTGGCCAT
GCCCCATCAAAGTTGCACTTAACCCAAGATTGTGGTGGAGCTTCCCATGTTTCTCG
TCTGTCCCGACGGTGTTGTGGTTGGTGCTTTCCTTACATTCTGAGCCTCTTTCCTTC
TAATCCACTCATCTGCATCTTCTTGTGTCCTTACTAATACCTCATTGGTTCCAAATTC
CCTCCCTTTAAGCACCAGCTCGTTTCTGTTCTTCCACAGCCTCCCAAGTATCCAAG
GGACTAAAGCCTCCACATTCTTCAGATCAGGATATTCTTGTTTAAGATGTTGAACTC
TATGGAGGTTTGTATGAACTGATGATCTAGGACCGGATAAGTTCCCTTCTTCATAG
CGAACTTATTCAAAGAATGTTTTGTGTATCATTCTTGTTACATTGTTATTAATGAAAA
AATATTATTGGTCATTGGACTGAACACGAGTGTTAAATATGGACCAGGCCCCAAAT
AAGATCCATTGATATATGAATTAAATAACAAGAATAAATCGAGTCACCAAACCACTT
GCCTTTTTTAACGAGACTTGTTCACCAACTTGATACAAAAGTCATTATCCTATGCAA
ATCAATAATCATACAAAAATATCCAATAACACTAAAAAATTAAAAGAAATGGATAATT
TCACAATATGTTATACGATAAAGAAGTTACTTTTCCAAGAAATTCACTGATTTTATAA
GCCCACTTGCATTAGATAAATGGCAAAAAAAAACAAAAAGGAAAAGAAATAAAGCA

CGAAGAATTCTAGAAAATACGAAATACGCTTCAATGCAGTGGGACCCACGGTTCAA
TTATTGCCAATTTTCAGCTCCACCGTATATTTAAAAAATAAAACGATAATGCTAAAAA
AATATAAATCGTAACGATCGTTAAATCTCAACGGCTGGATCTTATGACGACCGTTAG
AAATTGTGGTTGTCGACGAGTCAGTAATAAACGGCGTCAAAGTGGTTGCAGCCGG
CACACACGAGTCGTGTTTATCAACTCAAAGCACAAATACTTTTCCTCAACCTAAAAA
TAAGGCAATTAGCCAAAAACAACTTTGCGTGTAAACAACGCTCAATACACGTGTCA
TTTTATTATTAGCTATTGCTTCACCGCCTTAGCTTTCTCGTGACCTAGTCGTCCTCG
TCTTTTCTTCTTCTTCTTCTATAAAACAATACCCAAAGAGCTCTTCTTCTTCACAATT
CAGATTTCAATTTCTCAAAATCTTAAAAACTTTCTCTCAATTCTCTCTACCGTGATCA
AGGTAAATTTCTGTGTTCCTTATTCTCTCAAAATCTTCGATTTTGTTTTCGTTCGATC
CCAATTTCGTATATGTTCTTTGGTTTAGATTCTGTTAATCTTAGATCGAAGACGATTT
TCTGGGTTTGATCGTTAGATATCATCTTAATTCTCGATTAGGGTTTCATAGATATCAT
CCGATTTGTTCAAATAATTTGAGTTTTGTCGAATAATTACTCTTCGATTTGTGATTTC
TATCTAGATCTGGTGTTAGTTTCTAGTTTGTGCGATCGAATTTGTCGATTAATCTGA
GTTTTTCTGATTAACAGGGATCATCAACAAGTTTGTACAAAAAAGCAGGCTCTTTAA
AGTATTTTTACAACAATTACCAACAACAACAAACAACAAACAACATTACAATTACTAT
TTACAATTACAAAAAAAGTTAACATGGACAAGAAGTACAGCATCGGCCTGGCCATC
GGCACCAACTCTGTGGGCTGGGCCGTGATCACCGACGAGTACAAGGTGCCCAGC
AAGAAATTCAAGGTGCTGGGCAACACCGACCGGCACAGCATCAAGAAGAACCTGA
TCGGCGCCCTGCTGTTCGACAGCGGAGAAACAGCCGAGGCCACCCGGCTGAAGA
GAACCGCCAGAAGAAGATACACCAGACGGAAGAACCGGATCTGCTATCTGCAAGA
GATCTTCAGCAACGAGATGGCCAAGGTGGACGACAGCTTCTTCCACAGACTGGAA
GAGTCCTTCCTGGTGGAAGAGGATAAGAAGCACGAGCGGCACCCCATCTTCGGCA
ACATCGTGGACGAGGTGGCCTACCACGAGAAGTACCCCACCATCTACCACCTGAG
AAAGAAACTGGTGGACAGCACCGACAAGGCCGACCTGCGGCTGATCTATCTGGCC
CTGGCCCACATGATCAAGTTCCGGGGCCACTTCCTGATCGAGGGCGACCTGAACC
CCGACAACAGCGACGTGGACAAGCTGTTCATCCAGCTGGTGCAGACCTACAACCA
GCTGTTCGAGGAAAACCCCATCAACGCCAGCGGCGTGGACGCCAAGGCCATCCT
GTCTGCCAGACTGAGCAAGAGCAGACGGCTGGAAAATCTGATCGCCCAGCTGCCC
GGCGAGAAGAAGAATGGCCTGTTCGGCAACCTGATTGCCCTGAGCCTGGGCCTG
ACCCCCAACTTCAAGAGCAACTTCGACCTGGCCGAGGATGCCAAACTGCAGCTGA
GCAAGGACACCTACGACGACGACCTGGACAACCTGCTGGCCCAGATCGGCGACC
AGTACGCCGACCTGTTTCTGGCCGCCAAGAACCTGTCCGACGCCATCCTGCTGAG
CGACATCCTGAGAGTGAACACCGAGATCACCAAGGCCCCCCTGAGCGCCTCTATG
ATCAAGAGATACGACGAGCACCACCAGGACCTGACCCTGCTGAAAGCTCTCGTGC
GGCAGCAGCTGCCTGAGAAGTACAAAGAGATTTTCTTCGACCAGAGCAAGAACGG
CTACGCCGGCTACATCGATGGCGGAGCCAGCCAGGAAGAGTTCTACAAGTTCATC
AAGCCCATCCTGGAAAAGATGGACGGCACCGAGGAACTGCTCGTGAAGCTGAACA
GAGAGGACCTGCTGCGGAAGCAGCGGACCTTCGACAACGGCAGCATCCCCCACC
AGATCCACCTGGGAGAGCTGCACGCCATTCTGCGGCGGCAGGAAGATTTTTACCC
ATTCCTGAAGGACAACCGGGAAAAGATCGAGAAGATCCTGACCTTCCGCATCCCC
TACTACGTGGGCCCTCTGGCCAGGGGAAACAGCAGATTCGCCTGGATGACCAGAA
AGAGCGAGGAAACCATCACCCCCTGGAACTTCGAGGAAGTGGTGGACAAGGGCG
CCAGCGCCCAGAGCTTCATCGAGCGGATGACCAACTTCGATAAGAACCTGCCCAA
CGAGAAGGTGCTGCCCAAGCACAGCCTGCTGTACGAGTACTTCACCGTGTACAAC
GAGCTGACCAAAGTGAAATACGTGACCGAGGGAATGAGAAAGCCCGCCTTCCTGA

GCGGCGAGCAGAAAAAAGCCATCGTGGACCTGCTGTTCAAGACCAACCGGAAAGT
GACCGTGAAGCAGCTGAAAGAGGACTACTTCAAGAAAATCGAGTGCTTCGACTCC
GTGGAAATCTCCGGCGTGGAAGATCGGTTCAACGCCTCCCTGGGCACATACCACG
ATCTGCTGAAAATTATCAAGGACAAGGACTTCCTGGACAATGAGGAAAACGAGGAC
ATTCTGGAAGATATCGTGCTGACCCTGACACTGTTTGAGGACAGAGAGATGATCGA
GGAACGGCTGAAAACCTATGCCCACCTGTTCGACGACAAAGTGATGAAGCAGCTG
AAGCGGCGGAGATACACCGGCTGGGGCAGGCTGAGCCGGAAGCTGATCAACGGC
ATCCGGGACAAGCAGTCCGGCAAGACAATCCTGGATTTCCTGAAGTCCGACGGCT
TCGCCAACAGAAACTTCATGCAGCTGATCCACGACGACAGCCTGACCTTTAAAGAG
GACATCCAGAAAGCCCAGGTGTCCGGCCAGGGCGATAGCCTGCACGAGCACATT
GCCAATCTGGCCGGCAGCCCCGCCATTAAGAAGGGCATCCTGCAGACAGTGAAG
GTGGTGGACGAGCTCGTGAAAGTGATGGGCCGGCACAAGCCCGAGAACATCGTG
ATCGAAATGGCCAGAGAGAACCAGACCACCCAGAAGGGACAGAAGAACAGCCGC
GAGAGAATGAAGCGGATCGAAGAGGGCATCAAAGAGCTGGGCAGCCAGATCCTG
AAAGAACACCCCGTGGAAAACACCCAGCTGCAGAACGAGAAGCTGTACCTGTACT
ACCTGCAGAATGGGCGGGATATGTACGTGGACCAGGAACTGGACATCAACCGGCT
GTCCGACTACGATGTGGACGCTATCGTGCCTCAGAGCTTTCTGAAGGACGACTCC
ATCGATAACAAAGTGCTGACTCGGAGCGACAAGAACCGGGGCAAGAGCGACAAC
GTGCCCTCCGAAGAGGTCGTGAAGAAGATGAAGAACTACTGGCGCCAGCTGCTGA
ATGCCAAGCTGATTACCCAGAGGAAGTTCGACAATCTGACCAAGGCCGAGAGAGG
CGGCCTGAGCGAACTGGATAAGGCCGGCTTCATCAAGAGACAGCTGGTGGAAAC
CCGGCAGATCACAAAGCACGTGGCACAGATCCTGGACTCCCGGATGAACACTAAG
TACGACGAGAACGACAAACTGATCCGGGAAGTGAAAGTGATCACCCTGAAGTCCA
AGCTGGTGTCCGATTTCCGGAAGGATTTCCAGTTTTACAAAGTGCGCGAGATCAAC
AACTACCACCACGCCCACGACGCCTACCTGAACGCCGTCGTGGGAACCGCCCTG
ATCAAAAAGTACCCTAAGCTGGAAAGCGAGTTCGTGTACGGCGACTACAAGGTGT
ACGACGTGCGGAAGATGATCGCCAAGAGCGAGCAGGAAATCGGCAAGGCTACCG
CCAAGTACTTCTTCTACAGCAACATCATGAACTTTTTCAAGACCGAGATTACCCTGG
CCAACGGCGAGATCCGGAAGCGGCCTCTGATCGAGACAAACGGCGAAACAGGCG
AGATCGTGTGGGATAAGGGCCGGGACTTTGCCACCGTGCGGAAAGTGCTGTCTAT
GCCCCAAGTGAATATCGTGAAAAAGACCGAGGTGCAGACAGGCGGCTTCAGCAAA
GAGTCTATCCTGCCCAAGAGGAACAGCGACAAGCTGATCGCCAGAAAGAAGGACT
GGGACCCTAAGAAGTACGGCGGCTTCGACAGCCCCACCGTGGCCTATTCTGTGCT
GGTGGTGGCCAAAGTGGAAAAGGGCAAGTCCAAGAAACTGAAGAGTGTGAAAGAG
CTGCTGGGGATCACCATCATGGAAAGAAGCAGCTTCGAGAAGAATCCCATCGACT
TTCTGGAAGCCAAGGGCTACAAAGAAGTGAAAAAGGACCTGATCATCAAGCTGCC
TAAGTACTCCCTGTTCGAGCTGGAAAACGGCCGGAAGAGAATGCTGGCCTCTGCC
GGCGAACTGCAGAAGGGAAACGAACTGGCCCTGCCCTCCAAATATGTGAACTTCC
TGTACCTGGCCAGCCACTATGAGAAGCTGAAGGGCTCCCCCGAGGATAATGAGCA
GAAACAGCTGTTTGTGGAACAGCACAAACACTACCTGGACGAGATCATCGAGCAG
ATCAGCGAGTTCTCCAAGAGAGTGATCCTGGCCGACGCTAATCTGGACAAGGTGC
TGAGCGCCTACAACAAGCACAGAGACAAGCCTATCAGAGAGCAGGCCGAGAATAT
CATCCACCTGTTTACCCTGACCAATCTGGGAGCCCCTGCCGCCTTCAAGTACTTTG
ACACCACCATCGACCGGAAGAGGTACACCAGCACCAAAGAGGTGCTGGACGCCA
CCCTGATCCACCAGAGCATCACCGGCCTGTACGAGACACGGATCGACCTGTCTCA
GCTGGGAGGCGACGCCTATCCCTATGACGTGCCCGATTATGCCAGCCTGGGCAG

CGGCTCCCCCAAGAAAAAACGCAAGGTGGAAGATCCTAAGAAAAAGCGGAAAGTG
GAAGATGCTCCAAAGAAGAAGAGAAAGGTCGACGGCATTGGTAGTGGGAGCAACG
GCAGCAGCGGATCCAACGGTCCGACTGACGCCGCGGAAGAAGAACTTTTGAGCA
AGAATTATCATCTTGAGAACGAAGTGGCTCGTCTTAAGAAAGGTTCTGGCAGTGGA
GGTTCTGGCTCCGGATCTGGTGGTTCGGGCTCAGGCGGGTCCGGATCAGGCGAA
GAACTGCTTTCAAAGAATTACCACCTGGAAAATGAGGTAGCTAGACTGAAAAAGGG
GAGCGGAAGTGGGGGCTCCGGGTCGGGCTCAGGGGGCTCCGGTTCGGGAGGCT
CAGGGTCGGGGGAGGAGTTGCTGAGCAAAAATTATCATTTGGAGAACGAAGTAGC
ACGACTAAAGAAAGGGTCCGGATCGGGTGGTTCAGGATCTGGATCCGGAGGATCA
GGGTCCGGTGGGTCGGGCTCAGGAGAGGAGTTACTCTCGAAAAATTATCATCTCG
AAAACGAAGTGGCTCGGCTAAAAAAGGGCAGTGGTTCTGGAGGATCTGGGTCGG
GGTCAGGCGGGTCTGGATCTGGGGGATCTGGATCTGGTGAAGAGCTATTATCTAA
AAACTACCACCTCGAAAATGAGGTGGCACGCTTAAAAAAGGGAAGTGGCAGTGGT
GGGTCGGGATCTGGCTCTGGTGGCTCAGGCTCGGGAGGTTCAGGTTCCGGGGAA
GAGCTACTATCCAAGAATTATCATCTTGAGAACGAGGTAGCGCGTTTGAAGAAGGG
TTCCGGCTCAGGAGGATCTGGGTCAGGGTCAGGGGGTTCCGGGTCAGGCGGGTC
CGGGTCAGGCGAGGAACTGCTCTCGAAGAACTATCATCTTGAAAATGAGGTCGCT
CGATTAAAAAAGGGATCGGGCAGTGGTGGGTCCGGCTCCGGTTCCGGAGGATCG
GGATCTGGGGGCTCGGGATCCGGGGAGGAACTACTTTCAAAGAATTACCACCTCG
AAAACGAAGTAGCTCGATTAAAGAAAGGTTCAGGGTCGGGTGGCTCAGGTTCGGG
ATCAGGTGGGTCAGGCTCCGGTGGTTCAGGTTCGGGAGAAGAATTACTGAGTAAA
AATTATCATCTGGAAAATGAGGTAGCGAGACTAAAAAAGGGGAGTGGTTCTGGCG
GTTCGGGATCTGGCTCTGGGGGCTCTGGGTCGGGAGGGTCTGGGTCTGGCGAGG
AATTGCTATCGAAAAATTATCATCTTGAGAACGAAGTTGCTAGGCTCAAAAAGGGC
TCAGGCTCAGGCGGGTCCGGGTCAGGGTCGGGAGGTTCCGGATCCGGGGGATC
AGGCTCAGGGTAACCCGGGGGCGCGCCATGATAACTAGAGTCCTGCTTTAATGAG
ATATGCGAGACGCCTATGATCGCATGATATTTGCTTTCAATTCTGTTGTGCACGTTG
TAAAAAACCTGAGCATGTGTAGCTCAGATCCTTACCGCCGGTTTCGGTTCATTCTA
ATGAATATATCACCCGTTACTATCGTATTTTTATGAATAATATTCTCCGTTCAATTTA
CTGATTGTACCCTACTACTTATATGTACAATATTAAAATGAAAACAATATATTGTGCT
GAATAGGTTTATAGCGACATCTATGATAGAGCGCCACAATAACAAACAATTGCGTTT
TATTATTACAAATCCAATTTTAAAAAAAGCGGCAGAACCGGTCAAACCTAAAAGACT
GATTACATAAATCTTATTCAAATTTCAAAAGTGCCCCAGGGGCTAGTATCTACGACA
CACCGAGCGGCGAACTAATAACGCTCACTGAAGGGAACTCCGGTTCCCCGCCGG
CGCGCATGGGTGAGATTCCTTGAAGTTGAGTATTGGCCGTCCGCTCTACCGAAAG
TTACGGGCACCATTCAACCCGGTCCAGCACGGCGGCCGGGTAACCGACTTGCTG
CCCCGAGAATTATGCAGCATTTTTTTGGTGTATGTGGGCCCCAAATGAAGTGCAGG
TCAAACCTTGACAGTGACGACAAATCGTTGGGCGGGTCCAGGGCGAATTTTGCGA
CAACATGTCGAGGCTCAGCAGGACCGGCATGCAAGCTAGCTTACTAGTGATATTCT
ATAGTGTCACCTAAATCTGCGGC

<u>SunTagng-14aa</u>

NOS term

TET1cd

2xNLS
Linker
1xHA
sfGFP
scFV
UBQ10
Insulator
Omega RBC
dCAS9
3xNLS
GCN4 10x 14aa
OCS term

CACGGGGTGGTTTGGTTTAAACGTAACATAGATGACACCGCGCGATAATTTATC
CTAGTTTGCGCGCTATATTTTGTTTTCTATCGCGTATTAAATGTATAATTGCGGGAC
TCTAATCATAAAAACCCATCTCATAAATAACGTCATGCATTACATGTTAATTATTACA
TGCTTAACGTAATTCAACAGAAATTATATGATAATCATCGCAAGACCGGCAACAGG
ATTCAATCTTAAGAAACTTTATTGCCAAATGTTTGAACGATCGGGGAAATTCGAGCT
CAAACTTACGGCCATGGCGTAGGCGGCCTTTTCCGTTGTGATCTTCGCGGTCTCCT
CCTTGTTTTTGGACTAGTACCACCACCGCTACCACCACCTTCGGTTACCGTGAAGG
TTTTGGTAGCGTCGTCGTAGGTCCATTCACCGTCAACACCGTTGTCGTTAGCGTAC
TGTTTGAAAACTTTTTCCGCGGTAGCAGCGTCAACAGCTTCGGTGGTGGTTTCACC
TTTCAGGGTTTTACCGTTCAGGATAAGCTTGTACTCTTCGGTCCGAGATCCTCCTC
CCGTACGTCAGACCCAATGGTTATAGGGCCCCGCAACGTGTGTGAGAGCATAAGG
GGACACGGTGACAACATTGTCATGGGTTAATGTTAATGCTTTATGAGAAGGAATTT
GGTTCAATTCATTTACTTCAGAGGACTGTTCTGGACCTTCATTAGCTGCCTGGTCTT
TTTGCTCTGAGGCCTTCATTTTCTTATTCTTAGCTTCTTTAGCCTCAAACTTAATCTT
GTTTAGTTCAAAACCATGTTGGGGCTTATTTAGGTTTTTGTGCTGGTAAAAGACAAG
GGAGAGGCGGGTTGGATGATTACGGTTGGGGTGCTCAACAGGAGTGGTAGCGTG
CAGCTCTCGCCGGGCACACTCAATCAAAACCGAGCCGTGAGCAGGTGCGATGGC
CACCCCACCAATATTTGCATCCAAAAAGATGTGCTCACTGTCTGACCAATACTCATC
AATGTGGGGCAATTTCTCCTCAGCAGGTGACAGGGGGTCATCAGATAGGGGTTCG
TCTGATGGAGGCTCATCTGCTTCAGAATGCTGCTCATCTTCTTCCATTGGAGAAGA
GGCAAGGTCTTGAGGAGAGGTGAGGAAGGAGGGCTGGTGGTTTGGCTGATGAGG
CGTTAGCGGCTCAGTCACACCAGTGGAAGGCTCAGAATTAATGAGGGGCTCCATC
ACAGGAGCAGACAGGGTGGGGAGAGGAGCCACTTCGCCAAGCTGTGAAATGCCA
GGGCCATCAGCAGCTGCAGCATTGGCACCACTGAGTCTTCCCGAAGGCATCGTAC
AGTGGGGAGTGCTGCTTCTTTCTGAAAACCCGCATGAGGCTGTTGCGTCATTCTTC
AGTGGAGCTGGTGTGGCTGAAGCAGTCTTCGGGGACCAGGAGAAGCCTGGAGAT
GCCTCTTTCACTGGGTGAGGAGCGGATGGCATCAGCGAATAAGTTTTAGTGTTGTC
TGAACTTTTTAAGATAAAATGGGGTTCGGTTTCACTTTTTACTTCAGGTTGCACGGT
CTCAGTGTTACTCCCTAAGGTTGGCAGTGACGAAGGCTTACTGTTGTTTGTTGTTG
TTGAGTTATTCTTCCGCTTGATTCGGGGAATAGGTTTCTTTTCCACTGCCCTTATCT
TATGTGCAAGAACCTCTGTCATCATCGCAGCCCTCTTCTTTCCAGAACGGGGAACA

GGCTGAGTGAAACACGTTCTTTTTTTGCGGCGGGGTGCCAGGACCTCGATGGCCC
CAGATTTGATCTTGGCTTCCATTCCTTCCTTGGAGCCAAACTCATCTGTGTCTGAAA
GCTTATAAAGAGGTAGCACATGGAGCTGCTCATCTTGAGGAATAACACCCAAAGAG
CGGTTATCTTCTCGAGTTAAGGTACAAACCACAGTGCTTCCATTATTCATGTTGTGA
ATGTCCCTGTGGGGATGAGCACAGAAGTCCAGGCAAGCAGTGACCCCAGAGAAG
GGTCGACCTTCCTTGCTGCCAAGCCGACATTCTCGGGCAACATTTTCATATTCCAC
CTGATTTTGGTAAGCTACTGGAGCATACTGCTTATAAATTGGAGCTAATCGTGTAGC
CAAACTCTGTAAGTTATCTTCAAGGTTTTTTTCATGTAAGGGAGAGCTTGGATCAAT
TCTAAATCTTCTGGGGCTTGGGCTTCTACCAAACTTACAGCCATTAAAGTACATACT
CCATGAACAGCCAAAAGAGAATGAAGCTCCACAAGTCTCTGGATCAATTCCTTGAC
ATGTACAGGTACGATTTTCATTGAGGGTGCATCTTCTGTCGGTAGGGTGCCCATTG
TATGACTTTAGATTCTCTGTGAGCTCTGTGTATAGCCGGTCGGCCATTGGAAGAGG
GATGCCATCCCACACCATGATGAGCACCACCATCACAGCAGTTGGACAGTGGTGG
CCTGTACGCTGCCGGACCAAACAAAGAACTTTTTCTTCATCACTGCTTCTTCTTAAA
ACCCACTTAGCAATTGGACACCCATGAGAGCTTTTCCCTTCTTTACCGGTGTACAC
TACTATTTCTATCCTTATTGCGTTTCCTTTTTGACCATACCTATTCTCCATGATTTCC
CTGACAGCAGCAACACTTGGTCCTGCCCCAAGGTGTGTATAATATGGGCCTTTGTC
TTTTTGTATAACTCGATCAAGACAGCTGCAGGTGGGCAGCCCGGATCCGCCACCG
CCCACTTTCCGCTTTTTCTTAGGAGCTTCCACCTTGCGTTTTTTCTTGGGAGCGCC
GCTGCCCAGGCTGGCATAATCGGGCACGTCATAGGGATACGTACGACCGGTCCG
ACCTCCACCTTTGTAGAGCTCATCCATGCCATGTGTAATCCCAGCAGCAGTTACAA
ACTCAAGAAGGACCATGTGGTCACGCTTTTCGTTGGGATCTTTCGAAAGGACAGAT
TGTGTCGACAGGTAATGGTTGTCTGGTAAAAGGACAGGGCCATCGCCAATTGGAG
TATTTTGTTGATAATGGTCTGCTAGTTGAACGGAACCATCTTCAACGTTGTGGCGAA
TTTTGAAGTTAGCTTTGATTCCATTCTTTTGTTTGTCTGCCGTGATGTATACATTGTG
TGAGTTAAAGTTGTACTCGAGTTTGTGTCCAAGAATGTTTCCATCTTCTTTAAAATCA
ATACCCTTTAACTCGATACGATTAACAAGGGTATCACCTTCAAACTTGACTTCAGCA
CGCGTCTTGTAGGTCCCGTCATCTTTGAAAGATATAGTGCGTTCCTGTACATAACC
TTCGGGCATGGCACTCTTGAAAAAGTCATGCCGTTTCATGTGATCCGGATAACGGG
AAAAGCATTGAACACCATAGGTCAGAGTAGTGACAAGTGTTGGCCACGGAACAGG
TAGTTTTCCAGTAGTGCAAATAAATTTAAGGGTGAGTTTTCCGTTTGTAGCATCACC
TTCACCCTCTCCACGGACAGAAAATTTGTGCCCATTAACATCACCATCTAATTCAAC
AAGAATTGGGACAACTCCAGTGAAAAGTTCTTCTCCTTTGCTACCGCTTCCACCTC
CACCTGGATCCAAGCTTCCGCCGCCTCCTGAACCACCACCGCCACTACCTCCTCC
CCCAGAACCTCCGCCTCCACCAGCGTAATCTGGAACATCGTATGGGTAGCTGCTC
ACGGTCACCAGGGTGCCCTGGCCCCAGTAGTCGAACAGGCCGGTCACGCAGTAG
TACAGGGCGGTGTCGTCGCTGCGCACCTTGCTCATCTGCAGGTACACGGTGTTCT
TGCCGTTGTCCTTGCTGATGATGAAGCGGTCCTTCAGGGCGCTGTTGTAGTCGGT
GATGCCGTCGCCCCAGATCACGCCGATCCACTCCAGGCCGCGGCCGGGGGCCTG
GCGCACCCAGTTCACGCCGTAGTCGGTCAGGCTGAAGCCGCTCACGGCGCAGCT
CAGCTTCAGGCTGCCGCCGGGCTGCACCAGGCCGCCGCCGCTCTCCAGCAGCTT
CACCTCGCTGCCGCCGCCGCTGCTGCCGCCGCCGCCGCTGCCGCCGCCGCCGC
TGCCGCCGCCGCCGCGCTTCAGCTCCACCTTGGTGCCCTGGCCGAACACCCAGT
GGTTGCTGTACCACAGGGCGCAGAAGTAGGTGGCGAAGTCCTCGGGCTGCAGGC
TGCTGATGGTCAGGGTGGCCTTGTCGCCGATCAGGCTGCCGCTGAAGCGGCTGG
GCACGCCGGGGGCGCGGTTGTTGGTGCCGCCGATCAGGCCCTTGAACAGCTTGC

CGGGCTTCTCCTGCACCCAGCTGGCGTAGTTGCTGGTGGTCACGGCGCCGGTGC
TGCTGCGGCAGGTGATGGTCACGCGGTCGCCCACGCTGGCGCTCAGGCTGCTGG
GGCTCTGGGTCATCACGATGTCGGGGCCCATAAACCCTAGGTGTTAATCAGAAAA
ACTCAGATTAATCGACAAATTCGATCGCACAAACTAGAAACTAACACCAGATCTAGA
TAGAAATCACAAATCGAAGAGTAATTATTCGACAAAACTCAAATTATTTGAACAAAT
CGGATGATATCTATGAAACCCTAATCGAGAATTAAGATGATATCTAACGATCAAACC
CAGAAAATCGTCTTCGATCTAAGATTAACAGAATCTAAACCAAAGAACATATACGAA
ATTGGGATCGAACGAAAACAAAATCGAAGATTTTGAGAGAATAAGGAACACAGAAA
TTTACCTTGATCACGGTAGAGAGAATTGAGAGAAAGTTTTTAAGATTTTGAGAAATT
GAAATCTGAATTGTGAAGAAGAAGAGCTCTTTGGGTATTGTTTTATAGAAGAAGAA
GAAGAAAAGACGAGGACGACTAGGTCACGAGAAAGCTAAGGCGGTGAAGCAATAG
CTAATAATAAAATGACACGTGTATTGAGCGTTGTTTACACGCAAAGTTGTTTTTGGC
TAATTGCCTTATTTTTAGGTTGAGGAAAAGTATTTGTGCTTTGAGTTGATAAACACG
ACTCGTGTGTGCCGGCTGCAACCACTTTGACGCCGTTTATTACTGACTCGTCGACA
ACCACAATTTCTAACGGTCGTCATAAGATCCAGCCGTTGAGATTTAACGATCGTTA
CGATTTATATTTTTTTAGCATTATCGTTTTATTTTTTAAATATACGGTGGAGCTGAAA
ATTGGCAATAATTGAACCGTGGGTCCCACTGCATTGAAGCGTATTTCGTATTTTCTA
GAATTCTTCGTGCTTTATTTCTTTTCCTTTTTGTTTTTTTTTGCCATTTATCTAATGCA
AGTGGGCTTATAAAATCAGTGAATTTCTTGGAAAAGTAACTTCTTTATCGTATAACA
TATTGTGAAATTATCCATTTCTTTTAATTTTTTAGTGTTATTGGATATTTTTGTATGAT
TATTGATTTGCATAGGATAATGACTTTTGTATCAAGTTGGTGAACAAGTCTCGTTAA
AAAAGGCAAGTGGTTTGGTGACTCGATTTATTCTTGTTATTTAATTCATATATCAATG
GATCTTATTTGGGGCCTGGTCCATATTTAACACTCGTGTTCAGTCCAATGACCAATA
ATATTTTTTCATTAATAACAATGTAACAAGAATGATACACAAAACATTCTTTGAATAA
GTTCGCTATGAAGAAGGGAACTTATCCGGTCCTAGATCATCAGTTCATACAAACCT
CCATAGAGTTCAACATCTTAAACAAGAATATCCTGATCTGAAGAATGTGGAGGCTTT
AGTCCCTTGGATACTTGGGAGGCTGTGGAAGAACAGAAACGAGCTGGTGCTTAAA
GGGAGGGAATTTGGAACCAATGAGGTATTAGTAAGGACACAAGAAGATGCAGATG
AGTGGATTAGAAGGAAAGAGGCTCAGAATGTAAGGAAAGCACCAACCACAACACC
GTCGGGACAGACGAGAAACATGGGAAGCTCCACCACAATCTTGGGTTAAGTGCAA
CTTTGATGGGGCATGGCCAACAGAAGGATTAAAATGTGGCTTAGGGTGGGTGCTT
CGCGATCATACAGGGAAGGTGTTATGGTTAGGTGCAAGAGCTGTGGTAAAAGTAA
GAAGCGTGCTGGAAGTAGAAGTGGAGGCTCTTAGATGGGCTGTGCTGTCATTATC
CCGATTCAATTATAGGAAGATCATTTTTGAGGTGGATTCTCAGCAACTTGTGTCTTT
GGTTACATGAAAGTTATGCTTGTCAAGTCTCAATCCAATTATCCAAGACATAAAGTA
TCTACTTAGCAAGTTTGAGGATTTTATGCTTGTGCATACAAGCCGAGAAGGAAATG
GAGTGGCAGATAGAATAGCTAAGGAATCTCTTTCTTTTGAGAATTATGATCTAAAGT
TGTATTCTATTGTACCAATTTGGGTTAAAAGCTCTGTTGAGCTAGACTCATATGCAT
CCTAGGAAACAAGTTGTAATGAGTTGCTGGCCTCTCTTGGGATTGTTCATCAAAGT
AGCTGTCCCTACACACCACAACAGAATGGTGTTGTGGAGAGAAAGCACAGACACA
TCCTTGAGATGGCAAGGGCACTTAAGTTTCAGAGTGGTGTACCTACCAGGTTTTGG
GGAGACTGTGTCAAGACTGTTGTATACCTGATCAATAGACTCCCTACTCCAATCTTA
CAAGGCAAATGTCCATATGAACTACTCTATCAGAAACATGCCAAGCTTGATCACTT
GAGAGTGTTTGGGTGTCTTGCTTTTGCAGCAACACTGCCCAAAGGTGACAAACTTG
CACCTAGAGCCAAGCGAACCATCTTCATTGGTTATTCTGAGACTCAAAAGGGTTAC
AGGTTGTATGACTTGGATAATAAGGTGATCATAGTGAGCAGGGATGTAGTTTTCAG

AGAGTTTCAGTTCCCTTTCAAAGAGTCCATCTCCCATGAACCTGATATGTTCACTCG
AGCAGGTTCACTTAGCTCTGAAGATACAGCTTTGCAGCTAGTGTATAATGATATTTT
TCCTTCCAGCTTTCATACTGATGATGATGATACTCCAGTTCTTATTCCTGACATTGT
AGAGGCCAATGAGGATATCACTTTGGGGACTCATTCTACCCAAAATCATATCACTT
CAGCTGATGCTGCACCTTCAGGCCACCTGCATGCTCCTGCAGAGTCAGTTGAGTC
AGCTGAGCCTGACCTAGCAAATGCTGAATTTGAGCACCATACTGCTGTAGCTGATC
CCTCTCTAGTTCCAGCAAACCTGCACACTAGACCTAAACGCAATGCAGGTCCTCCC
ATCTGGCTCAAGGACTTTGTGACACTGAACAAAGGCTCTAGGGATGTTCCATACCC
TATAGCCAACTATGTTTCCTATGATCACTTGCCTGTTCACTATCAAGCTTATTTGAGT
GCTTTCTCCACTTATACTGAACCTAAATCCTTCAAAGAAGCAGCTCAAGATGAGAAA
TGGATGGAGGCCATGTCCCTTGAGATACAGGCTCTTGAGGATAATAACACCTGGG
AGATTGTCCCTTTACCCCCTGGTAAACAGCCTATAGGGTCCAAATGGGTGTACAAA
ATTAAATACAAAGCTAATGGTGAAGTTGACAGGTTTAAGGCAAGGCTAGTGGCCAA
GGGATACACTCAGCAAGAAGGCCTTGACTACCATGAAACTTTTTCTCCAGTGGCCA
AAATGGTCACTGTAAAAGCTGTCATATCTGTTGCTGCTTCCAAGGGCTGGTTCCTTT
TCCAGATGGATGTCAACAATGCTTTTCTACAAGGTGACCTCATGGAAGAAGTTTATA
TGTCTCTGCCTCAGGGTTTTCACAGCCAAGGGGAGACCAAAGTGTGCAGGCTGTT
GAATCCCTCTATGGTTTAAAGCAAGCATCAAGGCAGTGGAACATCAAGCTTACCAC
TGTCCTTATGCAGGCTGGTTTTATGCAAAGTGCTTATGATCACTCCTTGTTTACCAA
AAGAGAGGGGACTGACCTTGTCATAATCCTGATTTATGTGGATGATCTGTTAATAA
CAGGCAGCAGCAATGTTTTGATTTCAGAAGCAAAGGCAACCCTGCATCAGCATTTC
AAGATGAAAGATTTGGGAGAACTAAAATACTTCTTAGGCATTGAGGTGCTGAGATC
AGAAAAGGGAATCCTACTGAACCAGAGGAAGTATGCACTGGAACTGATATCAGGT
GTTGGTCTGGGAGGCTGCAGACCAGTGTCAACCCCAATGGAACAGAACCAAAGGC
TGACTACTGTAGAATATGACAAGCACTTAGGAAAGACTGATGATGCAGAGTTAGAG
GATGTGGGTTCCTATCAGAGACTGGTGGGAAAGCTTCTCTACTTGACAATCACAAG
GCCAGATATCAGCTTTGCAGTGCAGGTGCTGAGTCAATTCATGCAGCAACCCAAAC
AGTCACACCTGGAAGCAGCATTGAGGGTTGTGAAGTACATAAAAGGTTCTCCAGGT
GTTAGGAAGCGGCCGCATGCATATGAGTCTAGCTCAACAGAGCTTTTAACCCAAAT
TGGTACAATAGAATACAACTTTAGATCATAATTCTCAAAAGAAAGAGATTCCTTAGC
TATTCTATCTGCCACTCCATTTCCTTCTCGGCTTGTATGCACAAGCATAAAATCCTC
AAACTTGCTAAGTAGATACTTTATGTCTTGGATAATTGGATTGAGACTTGACAAGCA
TAACTTTCATGTAACCAAAGACACAAGTTGCTGAGAATCCACCTCAAAAATGATCTT
CCTATAATTGAATCGGGATAATGACAGCACAGCCCATCTAAGAGCCTCCACTTCTA
CTTCCAGCACGCTTCTTACTTTTACCACAGCTCTTGCACCTAACCATAACACCTTCC
CTGTATGATCGCGAAGCACCCACCCTAAGCCACATTTTAATCCTTCTGTTGGCCAT
GCCCCATCAAAGTTGCACTTAACCCAAGATTGTGGTGGAGCTTCCCATGTTTCTCG
TCTGTCCCGACGGTGTTGTGGTTGGTGCTTTCCTTACATTCTGAGCCTCTTTCCTTC
TAATCCACTCATCTGCATCTTCTTGTGTCCTTACTAATACCTCATTGGTTCCAAATTC
CCTCCCTTTAAGCACCAGCTCGTTTCTGTTCTTCCACAGCCTCCCAAGTATCCAAG
GGACTAAAGCCTCCACATTCTTCAGATCAGGATATTCTTGTTTAAGATGTTGAACTC
TATGGAGGTTTGTATGAACTGATGATCTAGGACCGGATAAGTTCCCTTCTTCATAG
CGAACTTATTCAAAGAATGTTTTGTGTATCATTCTTGTTACATTGTTATTAATGAAAA
AATATTATTGGTCATTGGACTGAACACGAGTGTTAAATATGGACCAGGCCCCAAAT
AAGATCCATTGATATATGAATTAAATAACAAGAATAAATCGAGTCACCAAACCACTT
GCCTTTTTTAACGAGACTTGTTCACCAACTTGATACAAAAGTCATTATCCTATGCAA

ATCAATAATCATACAAAAATATCCAATAACACTAAAAAATTAAAAGAAATGGATAATT
TCACAATATGTTATACGATAAAGAAGTTACTTTTCCAAGAAATTCACTGATTTTATAA
GCCCACTTGCATTAGATAAATGGCAAAAAAAAACAAAAAGGAAAAGAAATAAAGCA
CGAAGAATTCTAGAAAATACGAAATACGCTTCAATGCAGTGGGACCCACGGTTCAA
TTATTGCCAATTTTCAGCTCCACCGTATATTTAAAAAATAAAACGATAATGCTAAAAA
AATATAAATCGTAACGATCGTTAAATCTCAACGGCTGGATCTTATGACGACCGTTAG
AAATTGTGGTTGTCGACGAGTCAGTAATAAACGGCGTCAAAGTGGTTGCAGCCGG
CACACACGAGTCGTGTTTATCAACTCAAAGCACAAATACTTTTCCTCAACCTAAAAA
TAAGGCAATTAGCCAAAAACAACTTTGCGTGTAAACAACGCTCAATACACGTGTCA
TTTTATTATTAGCTATTGCTTCACCGCCTTAGCTTTCTCGTGACCTAGTCGTCCTCG
TCTTTTCTTCTTCTTCTTCTATAAAACAATACCCAAAGAGCTCTTCTTCTTCACAATT
CAGATTTCAATTTCTCAAAATCTTAAAAACTTTCTCTCAATTCTCTCTACCGTGATCA
AGGTAAATTTCTGTGTTCCTTATTCTCTCAAAATCTTCGATTTTGTTTTCGTTCGATC
CCAATTTCGTATATGTTCTTTGGTTTAGATTCTGTTAATCTTAGATCGAAGACGATTT
TCTGGGTTTGATCGTTAGATATCATCTTAATTCTCGATTAGGGTTTCATAGATATCAT
CCGATTTGTTCAAATAATTTGAGTTTTGTCGAATAATTACTCTTCGATTTGTGATTTC
TATCTAGATCTGGTGTTAGTTTCTAGTTTGTGCGATCGAATTTGTCGATTAATCTGA
GTTTTTCTGATTAACAGGGATCATCAACAAGTTTGTACAAAAAAGCAGGCTCTTTAA
AGTATTTTTACAACAATTACCAACAACAACAAACAACAAACAACATTACAATTACTAT
TTACAATTACAAAAAAAGTTAACATGGACAAGAAGTACAGCATCGGCCTGGCCATC
GGCACCAACTCTGTGGGCTGGGCCGTGATCACCGACGAGTACAAGGTGCCCAGC
AAGAAATTCAAGGTGCTGGGCAACACCGACCGGCACAGCATCAAGAAGAACCTGA
TCGGCGCCCTGCTGTTCGACAGCGGAGAAACAGCCGAGGCCACCCGGCTGAAGA
GAACCGCCAGAAGAAGATACACCAGACGGAAGAACCGGATCTGCTATCTGCAAGA
GATCTTCAGCAACGAGATGGCCAAGGTGGACGACAGCTTCTTCCACAGACTGGAA
GAGTCCTTCCTGGTGGAAGAGGATAAGAAGCACGAGCGGCACCCCATCTTCGGCA
ACATCGTGGACGAGGTGGCCTACCACGAGAAGTACCCCACCATCTACCACCTGAG
AAAGAAACTGGTGGACAGCACCGACAAGGCCGACCTGCGGCTGATCTATCTGGCC
CTGGCCCACATGATCAAGTTCCGGGGCCACTTCCTGATCGAGGGCGACCTGAACC
CCGACAACAGCGACGTGGACAAGCTGTTCATCCAGCTGGTGCAGACCTACAACCA
GCTGTTCGAGGAAAACCCCATCAACGCCAGCGGCGTGGACGCCAAGGCCATCCT
GTCTGCCAGACTGAGCAAGAGCAGACGGCTGGAAAATCTGATCGCCCAGCTGCCC
GGCGAGAAGAAGAATGGCCTGTTCGGCAACCTGATTGCCCTGAGCCTGGGCCTG
ACCCCCAACTTCAAGAGCAACTTCGACCTGGCCGAGGATGCCAAACTGCAGCTGA
GCAAGGACACCTACGACGACGACCTGGACAACCTGCTGGCCCAGATCGGCGACC
AGTACGCCGACCTGTTTCTGGCCGCCAAGAACCTGTCCGACGCCATCCTGCTGAG
CGACATCCTGAGAGTGAACACCGAGATCACCAAGGCCCCCCTGAGCGCCTCTATG
ATCAAGAGATACGACGAGCACCACCAGGACCTGACCCTGCTGAAAGCTCTCGTGC
GGCAGCAGCTGCCTGAGAAGTACAAAGAGATTTTCTTCGACCAGAGCAAGAACGG
CTACGCCGGCTACATCGATGGCGGAGCCAGCCAGGAAGAGTTCTACAAGTTCATC
AAGCCCATCCTGGAAAAGATGGACGGCACCGAGGAACTGCTCGTGAAGCTGAACA
GAGAGGACCTGCTGCGGAAGCAGCGGACCTTCGACAACGGCAGCATCCCCCACC
AGATCCACCTGGGAGAGCTGCACGCCATTCTGCGGCGGCAGGAAGATTTTTACCC
ATTCCTGAAGGACAACCGGGAAAAGATCGAGAAGATCCTGACCTTCCGCATCCCC
TACTACGTGGGCCCTCTGGCCAGGGGAAACAGCAGATTCGCCTGGATGACCAGAA
AGAGCGAGGAAACCATCACCCCCTGGAACTTCGAGGAAGTGGTGGACAAGGGCG

CCAGCGCCCAGAGCTTCATCGAGCGGATGACCAACTTCGATAAGAACCTGCCCAA
CGAGAAGGTGCTGCCCAAGCACAGCCTGCTGTACGAGTACTTCACCGTGTACAAC
GAGCTGACCAAAGTGAAATACGTGACCGAGGGAATGAGAAAGCCCGCCTTCCTGA
GCGGCGAGCAGAAAAAAGCCATCGTGGACCTGCTGTTCAAGACCAACCGGAAAGT
GACCGTGAAGCAGCTGAAAGAGGACTACTTCAAGAAAATCGAGTGCTTCGACTCC
GTGGAAATCTCCGGCGTGGAAGATCGGTTCAACGCCTCCCTGGGCACATACCACG
ATCTGCTGAAAATTATCAAGGACAAGGACTTCCTGGACAATGAGGAAAACGAGGAC
ATTCTGGAAGATATCGTGCTGACCCTGACACTGTTTGAGGACAGAGAGATGATCGA
GGAACGGCTGAAAACCTATGCCCACCTGTTCGACGACAAAGTGATGAAGCAGCTG
AAGCGGCGGAGATACACCGGCTGGGGCAGGCTGAGCCGGAAGCTGATCAACGGC
ATCCGGGACAAGCAGTCCGGCAAGACAATCCTGGATTTCCTGAAGTCCGACGGCT
TCGCCAACAGAAACTTCATGCAGCTGATCCACGACGACAGCCTGACCTTTAAAGAG
GACATCCAGAAAGCCCAGGTGTCCGGCCAGGGCGATAGCCTGCACGAGCACATT
GCCAATCTGGCCGGCAGCCCCGCCATTAAGAAGGGCATCCTGCAGACAGTGAAG
GTGGTGGACGAGCTCGTGAAAGTGATGGGCCGGCACAAGCCCGAGAACATCGTG
ATCGAAATGGCCAGAGAGAACCAGACCACCCAGAAGGGACAGAAGAACAGCCGC
GAGAGAATGAAGCGGATCGAAGAGGGCATCAAAGAGCTGGGCAGCCAGATCCTG
AAAGAACACCCCGTGGAAAACACCCAGCTGCAGAACGAGAAGCTGTACCTGTACT
ACCTGCAGAATGGGCGGGATATGTACGTGGACCAGGAACTGGACATCAACCGGCT
GTCCGACTACGATGTGGACGCTATCGTGCCTCAGAGCTTTCTGAAGGACGACTCC
ATCGATAACAAAGTGCTGACTCGGAGCGACAAGAACCGGGGCAAGAGCGACAAC
GTGCCCTCCGAAGAGGTCGTGAAGAAGATGAAGAACTACTGGCGCCAGCTGCTGA
ATGCCAAGCTGATTACCCAGAGGAAGTTCGACAATCTGACCAAGGCCGAGAGAGG
CGGCCTGAGCGAACTGGATAAGGCCGGCTTCATCAAGAGACAGCTGGTGGAAAC
CCGGCAGATCACAAAGCACGTGGCACAGATCCTGGACTCCCGGATGAACACTAAG
TACGACGAGAACGACAAACTGATCCGGGAAGTGAAAGTGATCACCCTGAAGTCCA
AGCTGGTGTCCGATTTCCGGAAGGATTTCCAGTTTTACAAAGTGCGCGAGATCAAC
AACTACCACCACGCCCACGACGCCTACCTGAACGCCGTCGTGGGAACCGCCCTG
ATCAAAAAGTACCCTAAGCTGGAAAGCGAGTTCGTGTACGGCGACTACAAGGTGT
ACGACGTGCGGAAGATGATCGCCAAGAGCGAGCAGGAAATCGGCAAGGCTACCG
CCAAGTACTTCTTCTACAGCAACATCATGAACTTTTTCAAGACCGAGATTACCCTGG
CCAACGGCGAGATCCGGAAGCGGCCTCTGATCGAGACAAACGGCGAAACAGGCG
AGATCGTGTGGGATAAGGGCCGGGACTTTGCCACCGTGCGGAAAGTGCTGTCTAT
GCCCCAAGTGAATATCGTGAAAAAGACCGAGGTGCAGACAGGCGGCTTCAGCAAA
GAGTCTATCCTGCCCAAGAGGAACAGCGACAAGCTGATCGCCAGAAAGAAGGACT
GGGACCCTAAGAAGTACGGCGGCTTCGACAGCCCCACCGTGGCCTATTCTGTGCT
GGTGGTGGCCAAAGTGGAAAAGGGCAAGTCCAAGAAACTGAAGAGTGTGAAAGAG
CTGCTGGGGATCACCATCATGGAAAGAAGCAGCTTCGAGAAGAATCCCATCGACT
TTCTGGAAGCCAAGGGCTACAAAGAAGTGAAAAAGGACCTGATCATCAAGCTGCC
TAAGTACTCCCTGTTCGAGCTGGAAAACGGCCGGAAGAGAATGCTGGCCTCTGCC
GGCGAACTGCAGAAGGGAAACGAACTGGCCCTGCCCTCCAAATATGTGAACTTCC
TGTACCTGGCCAGCCACTATGAGAAGCTGAAGGGCTCCCCCGAGGATAATGAGCA
GAAACAGCTGTTTGTGGAACAGCACAAACACTACCTGGACGAGATCATCGAGCAG
ATCAGCGAGTTCTCCAAGAGAGTGATCCTGGCCGACGCTAATCTGGACAAGGTGC
TGAGCGCCTACAACAAGCACAGAGACAAGCCTATCAGAGAGCAGGCCGAGAATAT
CATCCACCTGTTTACCCTGACCAATCTGGGAGCCCCTGCCGCCTTCAAGTACTTTG

ACACCACCATCGACCGGAAGAGGTACACCAGCACCAAAGAGGTGCTGGACGCCA
CCCTGATCCACCAGAGCATCACCGGCCTGTACGAGACACGGATCGACCTGTCTCA
GCTGGGAGGCGACGCCTATCCCTATGACGTGCCCGATTATGCCAGCCTGGGCAG
CGGCTCCCCCAAGAAAAAACGCAAGGTGGAAGATCCTAAGAAAAAGCGGAAAGTG
GAAGATGCTCCAAAGAAGAAGAGAAAGGTCGACGGCATTGGTAGTGGGAGCAACG
GCAGCAGCGGATCCAACGGTCCGACTGACGCCGCGGAAGAAGAACTTTTGAGCA
AGAATTATCATCTTGAGAACGAAGTGGCTCGTCTTAAGAAAGGTTCTGGCAGTGGA
GGTTCTGGCTCCGGAGGTTCCGGCGGGGAAGAACTGCTTTCAAAGAATTACCACC
TGGAAAATGAGGTAGCTAGACTGAAAAAGGGGAGCGGAAGTGGGGGCTCCGGGT
CGGGCGGCTCTGGGGGAGAGGAGTTGCTGAGCAAAAATTATCATTTGGAGAACGA
AGTAGCACGACTAAAGAAAGGGTCCGGATCGGGTGGTTCAGGATCTGGAGGTTCG
GGTGGGGAGGAGTTACTCTCGAAAAATTATCATCTCGAAAACGAAGTGGCTCGGC
TAAAAAAGGGCAGTGGTTCTGGAGGATCTGGGTCGGGGGGATCCGGGGGTGAAG
AGCTATTATCTAAAAACTACCACCTCGAAAATGAGGTGGCACGCTTAAAAAAGGGA
AGTGGCAGTGGTGGGTCGGGATCTGGCGGGTCGGGTGGAGAAGAGCTACTATCC
AAGAATTATCATCTTGAGAACGAGGTAGCGCGTTTGAAGAAGGGTTCCGGCTCAG
GAGGATCTGGGTCAGGGGGCTCAGGAGGGGAGGAACTGCTCTCGAAGAACTATC
ATCTTGAAAATGAGGTCGCTCGATTAAAAAAGGGATCGGGCAGTGGTGGGTCCGG
CTCCGGTGGTTCGGGGGGCGAGGAACTACTTTCAAAGAATTACCACCTCGAAAAC
GAAGTAGCTCGATTAAAGAAAGGTTCAGGGTCGGGTGGCTCAGGTTCGGGAGGAT
CCGGCGGTGAAGAATTACTGAGTAAAAATTATCATCTGGAAAATGAGGTAGCGAGA
CTAAAAAAGGGGAGTGGTTCTGGCGGTTCGGGATCTGGCGGGTCGGGAGGGGAG
GAATTGCTATCGAAAAATTATCATCTTGAGAACGAAGTTGCTAGGCTCAAAAAGGG
CTCAGGCTCAGGCGGGTCCGGGTCAGGGGGCTCAGGTGGCTAACCCGGGGGCG
CGCCATGATAACTAGAGTCCTGCTTTAATGAGATATGCGAGACGCCTATGATCGCA
TGATATTTGCTTTCAATTCTGTTGTGCACGTTGTAAAAAACCTGAGCATGTGTAGCT
CAGATCCTTACCGCCGGTTTCGGTTCATTCTAATGAATATATCACCCGTTACTATCG
TATTTTTATGAATAATATTCTCCGTTCAATTTACTGATTGTACCCTACTACTTATATGT
ACAATATTAAAATGAAAACAATATATTGTGCTGAATAGGTTTATAGCGACATCTATG
ATAGAGCGCCACAATAACAAACAATTGCGTTTTATTATTACAAATCCAATTTTAAAAA
AAGCGGCAGAACCGGTCAAACCTAAAAGACTGATTACATAAATCTTATTCAAATTTC
AAAAGTGCCCCAGGGGCTAGTATCTACGACACACCGAGCGGCGAACTAATAACGC
TCACTGAAGGGAACTCCGGTTCCCCGCCGGCGCGCATGGGTGAGATTCCTTGAAG
TTGAGTATTGGCCGTCCGCTCTACCGAAAGTTACGGGCACCATTCAACCCGGTCC
AGCACGGCGGCCGGGTAACCGACTTGCTGCCCCGAGAATTATGCAGCATTTTTTT
GGTGTATGTGGGCCCCAAATGAAGTGCAGGTCAAACCTTGACAGTGACGACAAAT
CGTTGGGCGGGTCCAGGGCGAATTTTGCGACAACATGTCGAGGCTCAGCAGGAC
CGGCATGCAAGCTAGCTTACTAGTGATATTCTATAGTGTCACCTAAATCTGCGGC
